# A high-quality *Buxus austro-yunnanensis* (Buxales) genome provides new insights into karyotype evolution in early eudicots

**DOI:** 10.1186/s12915-022-01420-1

**Published:** 2022-10-04

**Authors:** Zhenyue Wang, Ying Li, Pengchuan Sun, Mingjia Zhu, Dandan Wang, Zhiqiang Lu, Hongyin Hu, Renping Xu, Jin Zhang, Jianxiang Ma, Jianquan Liu, Yongzhi Yang

**Affiliations:** 1 State Key Laboratory of Herbage Improvement and Grassland Agro-ecosystems, College of Ecology, Lanzhou University, Lanzhou, China; 2 Key Laboratory of Bio-Resource and Eco-Environment of Ministry of Education & State Key Laboratory of Hydraulics & Mountain River Engineering, College of Life Sciences, Sichuan University, Chengdu, China; 3 CAS Key Laboratory of Tropical Forest Ecology, Xishuangbanna Tropical Botanical Garden, Chinese Academy of Sciences, Mengla, 666303 Yunnan China; 4 Center of Plant Ecology, Core Botanical Gardens, Chinese Academy of Sciences, Mengla, 666303 Yunnan China

**Keywords:** Eudicots, Buxales, Phylogenomic, Polyploidization, Ancestral eudicot karyotype, Karyotype evolution

## Abstract

**Background:**

Eudicots are the most diverse group of flowering plants that compromise five well-defined lineages: core eudicots, Ranunculales, Proteales, Trochodendrales, and Buxales. However, the phylogenetic relationships between these five lineages and their chromosomal evolutions remain unclear, and a lack of high-quality genome analyses for Buxales has hindered many efforts to address this knowledge gap.

**Results:**

Here, we present a high-quality chromosome-level genome of *Buxus austro-yunnanensis* (Buxales). Our phylogenomic analyses revealed that Buxales and Trochodendrales are genetically similar and classified as sisters. Additionally, both are sisters to the core eudicots, while Ranunculales was found to be the first lineage to diverge from these groups. Incomplete lineage sorting and hybridization were identified as the main contributors to phylogenetic discordance (34.33%) between the lineages. In fact, *B. austro-yunnanensis* underwent only one whole-genome duplication event, and collinear gene phylogeny analyses suggested that separate independent polyploidizations occurred in the five eudicot lineages. Using representative genomes from these five lineages, we reconstructed the ancestral eudicot karyotype (AEK) and generated a nearly gapless karyotype projection for each eudicot species. Within core eudicots, we recovered one common chromosome fusion event in asterids and malvids, respectively. Further, we also found that the previously reported fused AEKs in *Aquilegia* (Ranunculales) and *Vitis* (core eudicots) have different fusion positions, which indicates that these two species have different karyotype evolution histories.

**Conclusions:**

Based on our phylogenomic and karyotype evolution analyses, we revealed the likely relationships and evolutionary histories of early eudicots. Ultimately, our study expands genomic resources for early-diverging eudicots.

**Supplementary Information:**

The online version contains supplementary material available at 10.1186/s12915-022-01420-1.

## Background

Eudicots are the most diverse and abundant group of flowering plants on earth and contain more than 280,000 species from approximately 44 orders. Together, they make up over 75% of all flowering plants [1–3] and occur in almost all terrestrial ecosystems from the equator to the Arctic [2, 4]. In addition to their widespread distribution, they play important roles in maintaining ecosystems and the production of several foods and medicines [5]. Historically, fossil evidence suggests that eudicots arose in the early Cretaceous period (150-120 Million years ago [Mya]) [6, 7], and extant eudicots can be divided into one core lineage and four early-diverging lineages that includes Ranunculales, Proteales, Trochodendrales, and Buxales [8–10].

Polyploidization events, such as tetraploidization (whole-genome duplication, WGD) and hexaploidization (whole-genome triplication, WGT), occur frequently in plants and are a major source of evolutionary change that enables rapid adaptation to different environments [11–15]. Previously, the analysis of core eudicot genomes has revealed a common WGT event that has been designated the γ event [16–18]. However, this γ event was not found in *Nelumbo nucifera* (Proteales) [19, 20] or *Tetracentron sinense* (Trochodendrales) [21, 22]. Interestingly, genomic comparisons between *Aquilegia* (Ranunculales) and *Vitis* (Vitales, core eudicots) revealed a similar genomic fusion that appears to have occurred in the ancestral eudicot karyotype [23]. From this information, some authors have speculated that these two lineages may share one WGD and that the WGT occurred later in ancestral core eudicots. However, this genomic fusion could be an example of parallel homoplasy, since it may have occurred independently on different phylogenetic and evolutionary timescales by each individual lineage [24]. A recent study has reconstructed the most recent common ancestors at the three early-diverging eudicot nodes and found that such fusion events have not occurred in all ancestors [25]. Still, this study does not consider how phenotypes of karyotype evolved over time. In order to understand these changes, a careful analysis of karyotype evolution based on representative genomes from all lineages is needed to test this possibility [26].

In addition to the uncertainty concerning this WGT, the phylogenetic relationships between the well-defined core eudicot lineage [3, 27, 28] and the other four lineages are disputed [20–22, 29–32]. For example, phylogenetic analyses based on whole plastomes or multiple chloroplast genes have consistently suggested that Ranunculales, Proteales, Trochodendrales, and Buxales are successive sisters to the core eudicots [3, 28]. However, transcriptomic and genomic analyses of nuclear genes suggest that Trochodendrales and Buxales together comprise a clade that are sisters to the core eudicots [27]. Further, there are significant inconsistencies in the reported phylogenetic relationships within the core eudicots lineage (Additional file 1: Fig. S1) [3, 27, 28, 33–53]. High-quality chromosome-level genomes have been reported for several species that represent four of the five eudicot lineages [19–22, 54]. However, there is a lack of high-quality genome sequence data for Buxales, which has hindered efforts to clarify the phylogenetic relationships and karyotype evolutions of the early-diverging eudicot lineages.

Here, we present a high-quality chromosome-level reference genome for *Buxus austro-yunnanensis* (2n = 28) [25] that was generated by combining Oxford Nanopore Technologies (ONT) long reads, Illumina short reads, and Hi-C sequencing technologies. Using this genome, we reconstructed the phylogenetic relationships between five eudicot lineages, including a total of 25 eudicot orders. Our results indicate that Buxales and Trochodendrales are sisters, and together, they are also sister to the core eudicots, whereas Ranunculales was the first lineage to diverge. Incomplete lineage sorting (ILS) and hybridization accounted for 34.33% of the combined phylogenetic incongruities. Additionally, our data confirmed the independent occurrence of WGDs or WGTs in *B. austro-yunnanensis* and representative species of the other four lineages. Using high-quality early-diverging and core eudicot genomes, we reconstructed an accurate ancestral eudicot karyotype (AEK) and generated nearly gapless karyotype projections for the chosen eudicot species. Specifically, we observed one chromosome fusion within asterids and another in malvids and also confirmed that *Aquilegia* (Ranunculales) and *Vitis* (eudicots) have completely different karyotype evolution histories with no shared chromosome fusions. Thus, our results provide considerably new insights on the genomic and karyotype evolution of early-diverging eudicots.

## Results

### Genome assembly and annotation of *B. austro-yunnanensis*

**Fig. 1:**
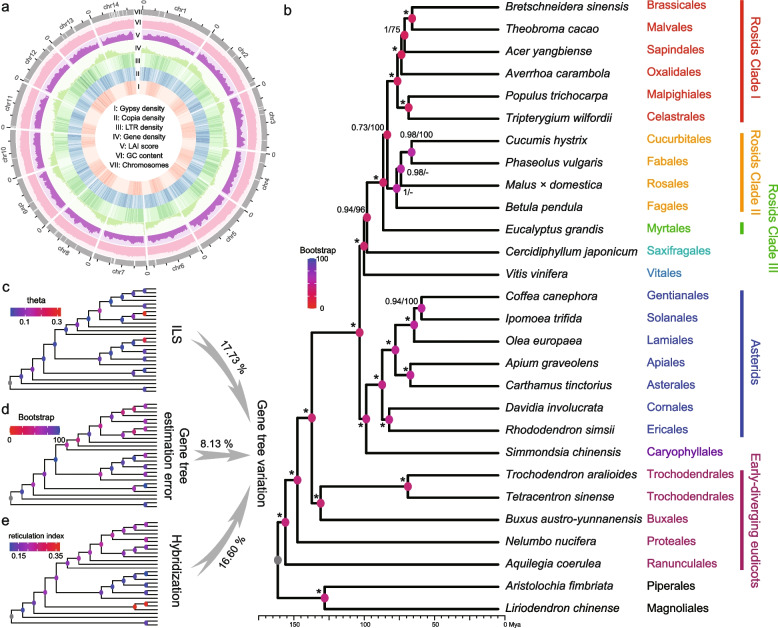
Features and phylogenetic analysis of the *B. austro-yunnanensis* genome. **a** Overview of the *B. austro-yunnanensis* genome. The outer layer of the circular, gray blocks represents the 14 chromosomes, and gaps within the chromosomes are shown in white. The various inner tracks represent the following genome features, calculated over 500 kb sliding windows: (I) *gypsy* density; (II) *copia* density; (III) LTR density; (IV) gene density; (V) LAI score; and (VI) GC content. **b** Phylogenetic tree of 28 species generated by coalescent analysis. Branch lengths represent divergence times. Posterior probabilities (PPs) and bootstrap support (BP) are indicated for each internal branch. Asterisks indicate 100 BP in concatenation analysis and 1.0 PP in coalescent analysis. Dots represent nodes with different topology. The nodal circles represent the gene tree variation calculated by the nodal recovery in the gene trees. **c** ILS. Nodes are colored by estimated theta values. **d** Gene tree estimation error. Nodes are colored by BP values that represent the percentage of nodes recovered from the simulation. **e** Hybridization. Nodes are colored by the Reticulation Index. Warmer colors indicate greater gene tree variation, higher ILS occurrence probabilities, higher gene tree estimation errors, and higher probabilities of hybridization in **b**, **c**, **d**, and **e**, respectively. Percentages of gene tree variation ascribed to ILS, estimation error, and gene flow are specified above the gray arrows

A total of 75.19 Gb raw ONT long reads were generated that produced ~113.26 × genome coverage based on the estimated genome size (663.90 Mb), which were used to construct the contig assembly with NextDenovo (Additional file 1: Fig. S2 and Table S1). After two rounds of polishing with the 31.88 Gb (48.02 ×) Illumina short reads, we obtained 112 final contigs with a total size of 637.31 Mb and an N50 size of 18.92 Mb (Additional file 1: Table S2). The continuity of this dataset exceeds previously published genomes of early-diverging eudicots (Additional file 1: Table S3). The 80.38 Gb (121.07 ×) of Hi-C data were then used to cluster and order the contigs into chromosomes, which lead to the successful construction of 14 chromosomes with lengths of 35.16 to 58.25 Mb. The total length of the chromosomes was 619.18 Mb, and they contained ~97.16% of the assembled sequences (Additional file 1: Fig. S3 and Table S4). We then assessed the quality of the *B. austro-yunnanensis* genome, which revealed that over 98.72% of the Illumina short reads could be mapped to the assembly. The GC content followed a Poisson distribution (Additional file 1: Fig. S4) and ~96.3% of the 2,121 BUSCO genes (eudicotyledons_odb10) were completely predicted, which is higher than with a previously published *B. sinica* genome (92.8%) and other early-diverging eudicot species (Additional file 1: Fig. S5). The LTR Assembly Index (LAI) was also calculated to assess the completeness of long-terminal repeat (LTR) retrotransposons. The *B. austro-yunnanensis* genome has a high LAI score of 12.64 (Fig. 1a), which is comparable to the scores reported for “reference” genomes [55]. Thus, the *B. austro-yunnanensis* genome shows high contiguity, completeness, and accuracy, which makes it suitable for further analysis.

Using a combination of homology and de novo approaches, we found that repeat sequences comprise 419.76 Mb (65.86%) of the *B. austro-yunnanensis* genome (Additional file 1: Table S5). Long terminal repeats (LTRs) were the most common repeat type that comprised 34.00% of the genome with intensive insertion occurring around ~0.39 Mya (Additional file 1: Fig. S6). We also predicted 25,542 protein-coding genes with average CDS lengths, exon lengths, and exon numbers of 1188.06 bp, 229.10 bp, and 5.19, respectively. These values are similar to those previously reported eudicots (Additional file 1: Fig. S7 and Table S6). Additionally, when the BUSCO assessment was applied to the predicted genes, 1946 (91.70%) of the BUSCO genes were found in *B. austro-yunnanensis*, which is more than the number found in *B. sinica* (Additional file 1: Table S7). Finally, almost 93.08% of the total predicted genes were assigned to entries in five functional databases by Blast searches (Additional file 1: Table S8). These results demonstrate that high quality gene annotation was achieved.

### Phylogenetic relationship between Buxales and other eudicot lineages

A total of 26 species from 25 eudicot orders and two magnoliids (*Aristolochia fimbriata* and *Liriodendron chinense* as outgroups) were selected for inclusion in our phylogenetic analyses (Additional file 1: Table S9). High-quality chromosome-level genomes are available for all of the chosen species. Of the 25 eudicot orders in this group, four represented early-diverging eudicot lineages, while the remaining 21 core eudicot orders were assigned to five major well-circumscribed clades: rosids (11 orders), asterids (7 orders), Saxifragales, Vitales, and Caryophyllales. Using the SonicParanoid algorithm, 1208 single-copy orthologous genes were identified in the 26 species. An unrooted tree was generated based on these nuclear genes using the coalescent-based method in ASTRAL. Most nodes in this tree had high posterior probabilities (≥ 0.94), but one had a posterior probability of only 0.73 (Fig. 1b and Additional file 1: Fig. S8).

The tree indicated that Ranunculales, Proteales, and Trochodendrales with Buxales are successively sister to the core eudicots, and that their divergence was dated to 156.26–136.83 Mya (Fig. 1b and Additional file 1: Fig. S8 and S9). Additionally, the diversification of core eudicots occurred at ~101.76 Mya. Within the core eudicots, asterids and Caryophyllales were identified as sister clades and together are sister to Rosids, Saxifragales and Vitales. We also found that Rosids and Saxifragales are sisters. Within rosids, neither malvids (Brassicales, Malvales, Sapindales and Myrtales) nor fabids (Oxalidales, Malpighiales, Celastrales, Cucurbitales, Fabales, Rosales and Fagales) clustered into a monophyletic clade. Instead, three clades were detected: clade I comprised only Myrtales, which is sister to all other rosids; clade II comprised four nitrogen-fixing orders (Fagales, (Rosales, (Fabales, Cucurbitales))); and clade III comprised the remaining six orders with Malpighiales and Celastrales, Oxalidales, and Sapindales successively labeled as sister to Brassicales and Malvales. Within the asterids, Lamiids (Solanales with Gentianales and Lamiales) are sister to Campanulids (Asterales and Apiales) and together are sister to Cornales and Ericales. The concatenation-based tree had a broadly similar topology but weakly supported a sister relationship between Rosales and Fagales within the rosid clade II (Additional file 1: Fig. S8).

Our results suggest that Caryophyllales are sister to asterids [3, 27, 28, 34–38, 56] but do not support previous findings that Caryophyllales are sister to other core eudicots [39–45]. Within rosids, the main conflicts with previous phylogenetic analyses related to the position of Myrtales and the phylogenetic relationships within clade II. Our results indicated that Myrtales are sister to other rosids, which is consistent with most analyses [37, 45–53, 56] but not with an analysis of 1000 transcriptomes that suggests that Myrtales are nested within other malvids [27]. Within rosid clade II, our results support the hypothesis that Fagales are sister to the other species and Cucurbitales are sister to Fabales, whereas previous studies have concluded that Fagales and Fabales cluster together and Cucurbitales and Rosales are sister clades [27, 43, 50]. For the five eudicot lineages, our results based on nuclear genes support a sister relationship between Buxales and Trochodendrales and indicate that they are together sister to all core eudicots. This result is similar with the 1000 plant transcriptomes analyses [27] and the recently published *B. sinica* genome research [25]. However, plastome evidence indicated that Buxales and core eudicots are sister clades (Additional file 1: Fig. S10).

It should be noted that more single copy genes were considered in this work than in most previous studies. Therefore, we took advantage of this large dataset to identify the main factors responsible for phylogenetic discordances within the generated trees. To this end, we compared the discordances between the gene trees and the species tree. Quartet scores were calculated for each internal branch and represent the support for three possible phylogenetic arrangements around the internal branch. Internal branches that exhibited incongruences with previous studies or the plastome tree also exhibited high discordance between the gene and species trees and had almost identical scores for all of the possible topologies (Additional file 1: Fig. S11). For example, 36%, 31%, and 33% of gene trees supported that Saxifragales and rosids, Vitales and rosids, and Vitales and Saxifragales are sister to each other. Additionally, 36%, 29%, and 35% of gene trees supported sister relationships between rosid clades I and II, clades II and III, and clades I and III, respectively. Finally, among the early-diverging eudicot lineages, 42%, 30%, and 27% of gene trees supported that Trochodendrales and Buxales, Buxales and core eudicots, and Trochodendrales and core eudicots are sister clades, respectively. These discordances were also displayed in a DensiTree analysis, which generated gene trees that clearly supported different topologies (Additional file 1: Fig. S12).

Although many factors could potentially be responsible for the incongruent topologies of the gene trees, we mainly focused on the relative contributions of three factors [30, 31, 57]: gene tree estimation error, incomplete lineage sorting, and hybridization. A relative importance decomposition analysis using the lmg algorithm showed that these three factors explained 42.45% of the total gene tree variation across the internal nodes (*R*^*2*^ = 0.4245) (Additional file 1: Fig. S13). Unlike the recent *B. sinica* paper that inferred ILS is the main cause for the gene tree discordances [25], we found both ILS and hybridization were the dominant factors that explain 17.73% and 16.60% of the total gene tree variation, respectively. Conversely, the gene tree estimation error explained only 8.13% of the observed variation. This could be due to the high orthology inference and relatively long sequence alignments (mean = 1483 bp) in our dataset. We further used the QuiBL method [58] to evaluate the hybridization with nine selected representative species (see method). In the species, 55.36% tested triplets showed significant evidence for hybridization (31 of 56, ΔBIC > 10) with an average ratio of the hybrid gene trees to be 16.38% (Additional file 1: Table S10 and S11), which showed a high hybridization occurrence in eudicots.

In addition to the overall assessment, we also compared three important nodes. We found the internal branches of the common ancestor of Buxales and Trochodendrales had the highest reticulation index and a lower theta value, which indicates that the hybridization should be the primary contributing factor that influences alternative positions for Buxales sister to Trochodendrales (Fig. 1c and e), while, for the discordance between Saxifragales, Vitales and rosids, and between the three rosid clades, hybridization and ILS may both act as the most important factors, since the internal branch of the ancestor of Saxifragales and rosids and the ancestor of rosid clade I and II both showed a high reticulation index and theta (Fig. 1c and e). In summary, our phylogeny results supported ILS and hybridization as dominant factors that influence gene tree topologies, and hybridization may contribute more to the relationships between the major lineages.

### Polyploidization histories in *B. austro-yunnanensis* and other eudicot orders

**Fig. 2:**
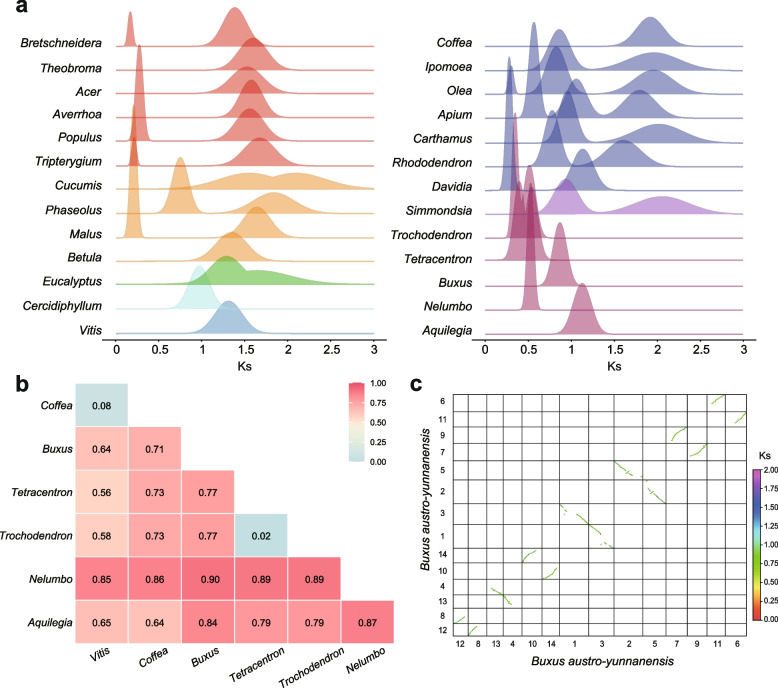
WGD analyses. **a** The *Ks* distributions of intragenomic synteny blocks. **b** The proportion of collinear gene trees supporting independent polyploidization between each species pair. **c** Synteny blocks of the *B. austro-yunnanensis* genome

We used multiple methods to explore the polyploidization histories of *B. austro-yunnanensis* and 25 other representative species from the 24 eudicot orders in the phylogenetic analyses. Homologous pairs from intragenomic and intergenomic syntenic blocks were identified and used to estimate the distributions of synonymous substitutions per synonymous site (*Ks*). Only one obvious polyploidization event was detected in *B. austro-yunnanensis* (*Ks* peak of ~0.87, Fig. 2a), consistent with the results of *B. sinica*, which indicates that this polyploidization event was shared by all *Buxus* species [25].

The intragenomic syntenic analysis of *B. austro-yunnanensis* showed that each of its chromosomes could be completely matched to another one without inter-chromosome variants (Fig. 2c). Intergenomic syntenic analyses between *B. austro-yunnanensis* and *Aristolochia fimbriata*, *Aquilegia coerulea*, *Nelumbo nucifera*, *Tetracentron sinense*, *Cercidiphyllum japonicum*, and *Vitis vinifera* yielded syntenic depth ratios of 2:1, 2:2, 2:2, 2:4 2:3, and 2:3, respectively, which confirms that only one WGD event occurred in the evolutionary history of *B. austro-yunnanensis* (Additional file 1: Fig. S14). We also clarified the WGD history of other selected representative species, most of which were consistent with previous reports. However, there were three notable exceptions (Additional file 1: Fig. S15). First, we identified an additional recent WGT event that occurred after the γ event in *Simmondsia chinensis* (Caryophyllales), even though no polyploidization was detected in this species in an earlier study [38]. Second, we found that the recent polyploidization of *Carthamus tinctorius* (Asterales) was a WGT rather than a WGD event [59]. Finally, we identified a WGT event in *Olea europaea* (Lamiales) that occurred between a previously reported WGD [60] and the ancient γ event.

We also performed phylogenetic analyses of collinear genes to determine whether WGDs that occurred in each early-diverging eudicot lineage are independent or shared with core eudicots. *Vitis vinifera* (Vitales) and *Coffea canephora* (asterids) were selected to represent core eudicots because neither of them showed evidence of any polyploidization event other than the γ event. We found that most gene trees supported independent WGD events that occur within each early-diverging eudicot order and with the core eudicots. Specifically, *Vitis vinifera* and *Coffea canephora* shared the γ event and two species of Trochodendrales (*Trochodendron aralioides* and *Tetracentron sinense*) shared two rounds of WGDs (Fig. 2b).

### Ancestral eudicot karyotype reconstruction and karyotype evolution

Synteny relationships between extant species are the basal information used to construct ancestral chromosomes; thus, high-quality chromosome-level genomes are important for inference [26]. Fission will disrupt synteny, although very rare [61], and fusion is the major inter-chromosome rearrangement type that also exists at a low frequency. Types of fusion include reciprocally translocated chromosome arms (RTA), end-end joining (EEJ), and nested chromosome fusion (NCF) [62–65]. Under such conditions, the ancestral chromosomes may be retained as independent chromosomes or entirely nested within the fused chromosomes among extant genomes. In our pairwise dot plots of the six representative species (five early-diverging eudicots: *Aquilegia*, *Nelumbo*, *Tetracentron*, *Trochodendron* and *Buxus*, and one core eudicot species *Vitis*), we observed many chromosome-scaled conserved synteny relationships (Additional file 1: Fig. S14 and S16). With these comparisons, the remaining challenge was to extract the ancestral chromosomes from such pairwise synteny relationships. To this end, we grouped all the chromosomes into seven clusters based on a *Z*-transformation from the percentage of collinear genes (Additional file 1: Fig. S17) that corresponded to the seven AEKs [26]. The most complete chromosome from each group (i.e., the one with the highest collinearity ratio) was selected as the reference AEK, and the synteny relationships of all seven reference AEKs were used to compare the chromosomes of the six representative species, which are shown covering all species chromosomes (Additional file 1: Figs. S18-24). The specific genes within the collinear blocks between the reference AEK and other chromosomes were then added to obtain the final AEK (see methods).

To test the performance of our reconstructed AEK, we mainly compared it with the previous AEK (henceforth referred to as the PAEK). The dot plots between AEK and PAEK showed that most of the chromosome variants were intra-chromosome variants, which supports high accuracy of conserved gene clustering. Only one inter-chromosome variant was detected where a piece of the PAEK 6 (corresponding to AEK 7) region was translocated into the head of AEK 6 (corresponding to PAEK 7) (Additional file 1: Fig. S25), which led to different chromosome boundaries and may cause incorrect inferences during karyotype evolution analysis. To solve this issue, we carefully checked which variant may be the most correct ancestral status. We found all five early-diverging species contain this translocation that was similar to AEK (Additional file 1: Fig. S26), which indicates that this translocation should be an ancestral state. Moreover, in the core eudicots, *Cercidiphyllum* and *Coffea* also contained this translocation, while *Vitis* did not (Additional file 1: Fig. S26 and S27). This comparison further indicated our reconstructed AEK could represent the most real ancestral status, and this error within PAEK may due to its representative species selection, which was mainly based on the *Vitis* genome [26]. During comparisons between AEK/PAEK and the extant species, we further found that mapping with our AEK yielded greater linearity and less gapped results than using the PAEK. Additionally, they also provided a more accurate gene order and more collinear gene information (Additional file 1: Figs. S26-S28).

**Fig. 3:**
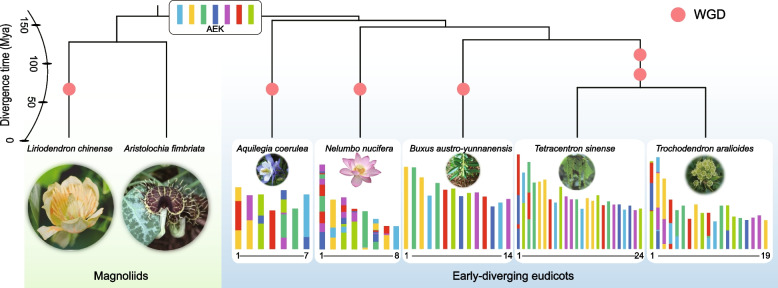
Karyotype projection of five early-diverging eudicot species. The topology is the same as in Fig. 1 and the branch length represent the divergence time (see detail in Additional file 1: Fig. S9). Polyploidization events are indicated by red dots (duplication)

**Fig. 4:**
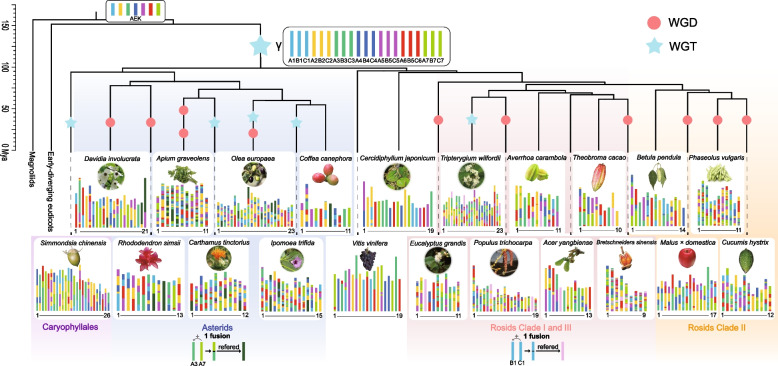
Karyotype projection of 21 core eudicot species. The topology is the same as in Fig. 1 and the branch length represent the divergence time (see detail in Additional file 1: Fig. S9). Different background colors represent different lineages corresponding to Fig. 1: light purple represents Caryophyllales, light blue represents asterids, light pink represents rosid clades I and III, and light yellow represent rosid clade II. Polyploidization events are indicated by red dots (duplication) and blue stars (triplication). Shared fusion events are marked below the corresponding lineages

The karyotype evolutionary history of eudicots was then inferred using the reconstructed AEK (Figs. 3 and 4 and Additional file 1: Figs. S29-S32). We found that most chromosomal variations between the orders are independent, especially in the basal eudicots where all variations that belong to each order are unique, even the chromosome rearrangements seen in the two species of Trochodendrales that occurred independently after their shared WGDs (Additional file 1: Fig. S33). This outcome is consistent with previous reports, since shared variations are usually considered to be “rare genome changes” that may reflect common ancestry [66].

After the γ event, there were three copies of each of the seven AEKs in the ancestor of the core eudicots. These ancestral chromosomes are designated A1-A7, B1-B7, and C1-C7, where the letters A, B, and C represent the duplicates. We observed one common chromosome fusion that occurred in all asterids where A3 fused with A7 via end-end joining (EEJ) (Fig. 4). This was confirmed by performing a pairwise synteny comparison among asterids (Additional file 1: Fig. S34). As the gene loss and gain in homolog chromosomes can be formed by the recent polyploidization after the γ event, not all fusion positions can be totally retained in each species. Therefore, we used *Davidia involucrate* as a reference because it retained two fusion positions that corresponded to its recent WGD events and has less chromosome changing. In synteny comparison, if there is one identical fusion position detected in other species, and the continuous intact collinear gene blocks in each other asterids species were detected, then we consider that these species shared the EEJ event, (Additional file 1: Fig. S34). It should be noted that a similar fusion from A3 and A7 also exists in *Simmondsia* (Caryophyllales), but the fusion position differs from that in the asterids. Specially, the collinearity near the dash line was discontinuous, which indicates that the occurrence of the fusion in these two clades is an example of parallel evolution (Additional file 1: Fig. S34). Conversely, only one shared fusion pattern was detected between rosid clades I and III, which was the B1 and C1 connected via EEJ (Additional file 1: Fig. S35). Using the *Tripterygium wilfordii* as a reference, we found all other rosid clade I and III species shared the same fusion position. However, careful analysis of all studied species within rosid clade II (i.e., *Malus*, *Cucumis*, and *Phaseolus*) revealed a complete absence of this fusion. A fusion of B1 and C1 was also detected in *Betula*, but the fusion position was different, which indicates that this fusion event was independent compared to the events in rosid clades I and III. It should also be noted that species in rosid clades I and III were previously considered to cluster together in the malvids clade [3, 27, 28], but they are separated in our phylogenetic analyses (Fig. 1b). Based on a combined analysis of their phylogeny and karyotype evolutionary histories, we suggest that malvids species should still cluster together, and karyotype analyses may reflect their true relationship better than gene-based phylogenetic analysis alone [26, 67].

### The polyploidization histories of *Aquilegia* and core eudicots

**Fig. 5:**
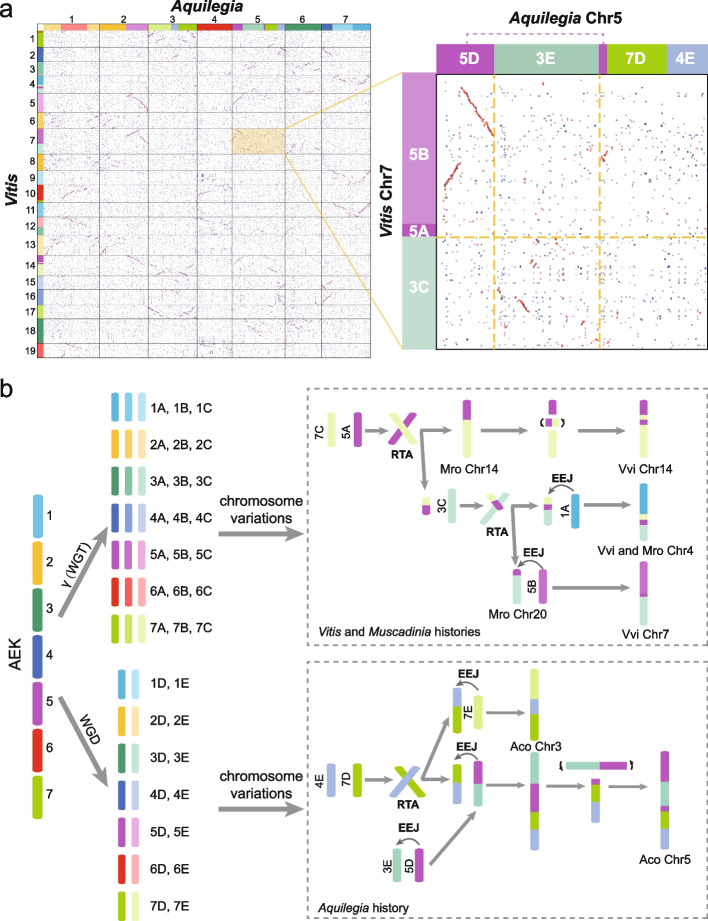
Karyotype evolution of *Aquilegia*, *Muscadinia*, and *Vitis*. **a** Collinear gene dot plots and karyotype projections between *Aquilegia* and *Vitis*. The enlarged, inset part indicates that there is no sharing variation event between *Aquilegia* chr 5 and *Vitis* chr 7. **b** Possible karyotype evolution histories of *Aquilegia*, *Vitis*, and *Muscadinia*. Aco, Mro, and Vvi are the abbreviations for *Aquilegia*, *Muscadinia*, and *Vitis*, respectively. RTA and EEJ are the abbreviations for reciprocally translocated chromosome arms and end-end joining

Although our collinear gene tree analyses suggested that independent WGD events occurred in the early-diverging eudicot lineages and core eudicots, an earlier study suggested a shared WGD between *Aquilegia* and *Vitis* based on the occurrence of a similar fusion event in both orders [23]. To directly resolve this disagreement, we carefully investigated the polyploidization history of all early-diverging eudicot lineages and two core eudicots (*Vitis* and *Cercidiphyllum*). Our karyotype analysis revealed a similar fusion of AEK 3 (green) and 5 (purple) in *Aquilegia* Chr 5 and *Vitis* Chr 7 that is absent in other early-diverging eudicots (*Nelumbo*, *Trochodendron*, *Tetracentron*, and *Buxus*) and core eudicot *Cercidiphyllum* (Figs. 3 and 4). However, the fusion position in *Aquilegia* was completely different to that in *Vitis*, which indicates that the fusions occurred in separate events (Fig. 5a). This conclusion was supported by the karyotype evolution histories of the two orders. Within *Aquilegia*, AEK 3 and 5 were connected via EEJ, and they further connected to another chromosome, which formed by an RTA (Reciprocally translocated chromosome arms) event between AEK 4 and 7 (Fig. 5b). A subsequent intra-chromosome inversion then created the current *Aquilegia* Chr 5 (Fig. 5b). The connection of AEK 3 and 5 in *Vitis* is more complex where two RTA events occurred in AEK 3, 5 and 7. Following, another copy of AEK 5 was joined through EEJ to form the *Vitis* Chr 7. This evolutionary history was confirmed by analyzing another Vitaceae species, *Muscadinia*, which has one complete chromosome (Chr 20) that was formed by the two RTA events involving AEK 3, 5, and 7, but without the subsequent EEJ (Fig. 5b). Thus, *Aquilegia* and *Vitis* have very different karyotype evolutionary histories that involve different types of fusion (RTA and EEJ) and different fusion positions. This data strongly supports the occurrence of independent WGDs in early-diverging eudicots and core eudicots. Overall, our results show that only considering the phenotype of ancestral chromosome fusions without investigating the details of the associated chromosome rearrangements may lead to unreliable results and conclusions.

## Discussion

Our assembled *Buxus austro-yunnanensis* genome displayed high quality of continuity, completeness, and accuracy when compared to many other previous studies on early-diverging eudicot species [19, 21, 25, 54, 68]. Such a high-quality genome ensured all the following analyses and revealed the evolutionary history of Buxales with other eudicots under the assistance of phylogeny and karyotype analyses.

Polyploidization is a common phenomenon in plants and is considered a major force that causes rapid genome evolution and enhances adaptations thus increases biodiversifications [69, 70]. As a concerning problem, many methods have been proposed to detect polyploidization in plants, including the determination of the *Ks* distribution of synteny genes, which is one of the most common ways. The peak of a *Ks* distribution within one species usually indicates one polyploidization event, but this characteristic is not able to distinguish how many times the genome multiplies in an event. Further, it is also difficult to determine whether a duplication event is shared or not between two species as evolutionary rates vary and cause the *Ks* to differ greatly [30, 71]. Another method uses dot plots to display the presence of large syntenic regions within a genome for a more straightforward comparison that circumvents the shortcoming of *Ks* analysis in regards with determining ploidy. However, it often depends on the well assembled genomes, especially at the chromosome-level, to get accurate results [72, 73]. In addition to these methods, phylogenetic analysis can also be applied to estimate polyploidizations through gene count data where the number of gene copies in various gene families across a group of taxa along the phylogeny are counted with consideration of gene birth and death rates [12, 74]. Since it normally only uses the orthologous genes to infer phylogenetic relationships in each gene family, this method relies on the annotation quality of the genes, and less on the quality of the genome and gene order, which makes it easy to apply when transcriptome analyses are combined [75, 76]. Still, the major challenges with this method concern how orthologous genes are obtained and the accuracy of inferred gene birth and death rates. A simplified way to realize this problem uses synteny genes between two species to directly calculate the number of gene trees that support shared or independent polyploidizations [31]. With this method, only the accuracy of collinear genes should be assigned and the computational resources required are relatively high. Here, we used the combination of these methods to achieve better genome analysis with a satisfactory outcome that is highly consistent. Additionally, this combined method also increased accuracy, which corrected several inappropriate determinations of duplications, such as the polyploidization histories of *S. chinensis*, *C. tinctorius*, and *O. europaea* (Additional file 1: Fig. S15). Therefore, the analyses of polyploidizations need to be considered more thoroughly in the way of being validated by multiple methods to achieve accurate results.

Previously, it has also been found that using orthologous gene-based phylogeny analyses to reveal evolutionary relationships of eudicots is a challenge because of the complicated polyploidizations that majorly reduce the detection of single-copy orthologous genes (SCOGs) [30]. To circumvent this issue, we utilized a reciprocal blast method by SonicParanoid to identify more SCOGs and improve their accuracy for phylogenomic analyses. Our analysis yielded 1,208 SCOGs, which is much larger than other previous eudicot phylogeny studies [27, 36]. Based on this dataset, we have revealed that ILS and hybridization were the two dominant factors that contribute to the total gene tree variations (Fig. 1 and Additional file 1: Fig. S13). Under these influences, some nodes showed low support and need more evidence to declare their relationships (Fig. 1 and Additional file 1: Fig. S8). To help define these relationships, ancestral karyotypes could also be used as an assist. Ancestral karyotypes identify common genes and their order in different species, which provides powerful evidence to reflect the evolutionary history of plant, and is a useful tool to detect common evolutionary histories among species [26, 77]. Currently, present methods for ancestral karyotype construction mainly depend on common collinear genes in several species [26, 78, 79]. The genes obtained under these conditions usually produce low amounts, which often cause gaps during projections. These types of projections often reveal limited karyotype information, especially for species that underwent frequent chromosome variation. Ultimately, these issues create misleading information that assume that similar karyotype fragments are shared, and is not able to infer accurate evolutionary histories, which was seen with previous research in *Aquilegia* [23]. In our research, we used a new method to reconstruct the ancestor chromosomes of the eudicots based on the concept that intact ancestral chromosomes are still nested in present species although after many variations. Among the chromosome changing, fission is very rare in animals [61] or in plants that are overserved in the selected six eudicots (Additional file 1: Fig. S16). Therefore, major inter-chromosome rearrangement types seem to rely more on fusion, which ensure us to detect the intact ancestral chromosomes in the extant genomes with our comparisons (Additional file 1: Figs. S18-24).

Based on this concept, we clustered chromosomes in the six selected eudicots and successfully obtained seven groups. After additional processes that added specific genes into each picked chromosome, the final reconstructed AEK displayed more precise and detailed results, which can be reflected in the comparison of karyotype projections by PAEK and AEK (Additional file 1: Fig. S27), and the rectification of improper translocation phenomenon in PAEK (Additional file 1: Figs. S25-27). It also provides powerful evidence for dissolving ambiguous phylogeny relationships, which is reflected in our analyses of malvids and Myrtales, Asterids and Caryophyllales, and with *Aquilegia* and *Vitis* (Fig. 5 and Additional file 1: Fig. S34 and S35). With this new dataset, more comprehensive karyotypes can be obtained. Ultimately, the method we used for construction can be applied universally towards a variety of species, which will undoubtedly facilitate more comparisons and inferences in the evolutionary histories among species.

These analyses also emphasized the importance of considering continuity of collinearity near transition points of different fragments. It is because only collinearity pass though those points continuously can this variation of karyotype be regarded that was happened before speciation and therefore shared by those species. Conversely, karyotypes that show similar patterns, but have discontinuous collinearity, may just be coincidence. Although this standard puts less effort on the possibility that shared chromosome variation may undergo further changes, it can avoid many false positive identifications and become more reliable. Additionally, inference of phylogeny solely based on sequence is often affected by interference, including the phylogenetic discordances in our analyses. Still, according to this AEK dataset, it is clear which linages should be grouped together by analyzing their karyotype features (i.e., the relationship of Myrtales and rosid clade I). Since further karyotype construction of more species can be carried out, it will be more advantageous and more reliable to analyze phylogeny considering evidence from both sequences and karyotype features.

## Conclusions

In this work, high-quality chromosome-level genome sequences of *Buxus austro-yunnanensis* were obtained that enrich the genomic resources available for the early-diverging eudicot order Buxales. By analyzing this genome together with representative genomes of early-diverging eudicots and other core eudicot lineages, we inferred their phylogenetic relationships and showed the relative contribution of gene tree estimation errors, ILS, and hybridization to observed phylogenetic discordance. Further, we reconstructed a better ancient eudicot karyotype (AEK) using high-quality genomic information on early-diverging eudicots and other core eudicots, and clarified the karyotype evolution of various eudicot orders, which revealed one common fusion in asterids and another in malvids. We also reconstructed and confirmed the different karyotype evolution histories of *Aquilegia* and *Vitis* based on the AEK. Together, this information suggests that high resolution reconstruction of ancestral chromosomes provides significant insights into karyotype evolutionary processes that are not accessible by other means. When judging common chromosome variants, it is essential to fully consider the karyotype evolution process to avoid misinterpreting outcomes that result from parallel evolution. These findings provide new insights into the evolution of eudicots and will inspire more karyotype analysis and genomic research into these species, as well as others in the future.

## Methods

### Sample collection and sequencing

The sampled *B. austro-yunnanensis* individual was planted in the Xishuangbanna Tropical Botanical Garden at the Chinese Academy of Sciences, Mengla 666303, Yunnan, China; stored samples were assigned the accession number 0020010744. Fresh leaves were collected, immediately frozen in liquid nitrogen, and sent to Grandomics (Wuhan, China) for genome sequencing. Total genomic DNA was extracted using the CTAB method and purified with the QIAGEN® Genomic kit (Cat#13343, QIAGEN). For the ONT long reads, a library of large DNA fragments (> 20 kb) was constructed with the Ligation Sequencing Kit 1D (SQK-LSK108) and then sequenced on the PromethION platform. Raw long reads were further filtered by removing adaptors and low-quality nucleotides (mean quality score < 7). For Illumina short reads, a paired sequencing library with an insertion size of 350 bp was constructed and sequenced using the Illumina HiSeq 4000 platform. The raw Illumina reads were then filtered with fastp v0.20.1 [80] using the default parameters. We also performed Hi-C (high-throughput chromosome conformation capture) sequencing after fixing fresh leaves in formaldehyde solution (1%), cross-linking the chromatin, and digesting it with the restriction enzyme Dpn II. Finally, a library was constructed and sequenced using the Illumina HiSeq 4000 platform.

### Genome assembly

The genome size was first estimated using GCE v1.0.0 [81] with K-mer size of 19 bp and cleaned short reads. Nextdenovo v2.3.0 (https://github.com/Nextomics/NextDenovo) was then used to correct the ONT long reads and construct the preliminary contig assembly with the following parameters “read_cutoff=7k, seed_cutoff=37072, blocksize=8g.” The assembly was then subjected to two rounds of polishing with Nextpolish v1.2.4 [82] using the corrected ONT long reads and the cleaned short reads to generate the final contig assembly. The GC content, short reads coverage, and BUSCO v3.0.2 [83] (embryophyta_odb10) analyses were used to evaluate the genome assembly’s quality. To obtain the chromosome-level assembly, the raw clean Hi-C data were filtered and mapped to contigs with HiC-Pro [84], after which the 3D-DNA pipeline was used to cluster, sort, and orientate contigs into chromosomes based on interaction relationships [85].

### Genome annotation

Repetitive elements were first annotated using a combination of evidence-based and *de novo* approaches. TRF v4.09 [86] was used to identify tandem repeats. RepeatMasker v4.1.0 [87] and RepeatproteinMask (a package within RepeatMasker) were used to search repeat elements in our assembly against a known repeat database (Repbase v21.01). For de novo annotation, we combined repeat libraries generated with LTR_retriever v2.9.0 [88] and RepeatModeler v2.0 (http://www.repeatmasker.org/RepeatModeler.html) and then used RepeatMasker to search the repeat sequences against the combined library. The insertion times of complete LTRs were inferred using LTR_retriever. To predict protein coding genes, we first used GeMoMa v1.6.1 [89, 90] to perform homology-based gene searching using the following reference species: *Nymphaea colorata*, *Nymphaea thermarum*, *Aquilegia coerulea*, *Tetracentron sinense*, *Trochodendron aralioides*, *Vitis vinifera*, and *Arabidopsis thaliana*. Three programs (AUGUSTUS v3.3.3 [91], GENSCAN [92], GlimmerHMM [93]) were used for de novo prediction. The GENSCAN and GlimmerHMM predictions were based on an *Arabidopsis thaliana* training set, while a *B. austro-yunnanensis* training set generated during the BUSCO analyses was used with Augustus. The Buxus sempervirens transcript (SRR9304495) assembled with trinity [94] was also aligned to the genome using PASA to obtain transcriptomic evidence [95]. Finally, EvidenceModeler v1.1.1 [96] was used to integrate the genes predicted by the homology and de novo approaches and obtain a consensus gene set. The final gene set was produced by removing low-quality genes with premature termination. Gene functions were assigned by BLASTP searching (e value ≤ 1e−5) against the NCBI NR, SwissProt, and TrEMBL protein databases. Motifs, domains and Gene Ontology (GO) information were extracted using InterProScan v5.52-86.0 [97]. Metabolic pathways were annotated with KAAS [98], using a bi-directional best-hit strategy to assign KEGG orthology terms to each gene.

### Phylogeny analyses

Phylogenetic analysis was performed using two magnoliids (*Aristolochia fimbriata* and *Liriodendron chinense*) as outgroups together with 26 species representing 25 eudicot orders (Additional file 1: Table S9). The eudicot orders represented in the analysis were Apiales (*Apium graveolens*), Asterales (*Carthamus tinctorius*), Brassicales (*Bretschneidera sinensis*), Buxales (*B. austro-yunnanensis*), Caryophyllales (*Simmondsia chinensis*), Celastrales (*Tripterygium wilfordii*), Cornales (*Davidia involucrata*), Cucurbitales (*Cucumis hystrix*), Ericales (*Rhododendron simsii*), Fabales (*Phaseolus vulgaris*), Fagales (*Betula pendula*), Gentianales (*Coffea canephora*), Lamiales (*Olea europaea*), Malpighiales (*Populus trichocarpa*), Malvales (*Theobroma cacao*), Myrtales (*Eucalyptus grandis*), Oxalidales (*Averrhoa carambola*), Proteales (*Nelumbo nucifera*), Ranunculales (*Aquilegia coerulea*), Rosales (*Malus × domestica*), Sapindales (*Acer yangbiense*), Saxifragales (*Cercidiphyllum japonicum*), Solanales (*Ipomoea trifida*), Trochodendrales (*Tetracentron sinense* and *Trochodendron aralioides*), and Vitales (*Vitis vinifera*). A total of 1208 single copy gene families were identified among the 28 species by SonicParanoid v1.0 [99], and amino acid sequences for each gene were aligned using MAFFT v7.453 [100]. The DNA sequences were then aligned based on the corresponding amino acid alignments using PAL2NAL v14 [101]. All of the aligned sequences were concatenated and used to build a maximum likelihood (ML) tree using IQ-TREE v2.1.3 [102] with the automatically selected best-fit substitution model (-m MFP) and the 1000 ultrafast bootstrap approximation (-B 1,000). Gene trees were also constructed with IQ-TREE and then imported into ASTRAL v5.7.3 [103] for coalescent-based species tree inference with quartet scores and posterior probabilities. A density tree of all gene trees was generated using the DensiTree function within ggtree v3.2.1 [104–106] to clearly reveal topological discordances. Divergence times for single copy genes were estimated using MCMCTree from the PAML package [107]. We set the burn-in value to 1,000,000 iterations, and the MCMC process was performed 20,000 times with a sampling frequency of 200. Two fossil constraints were selected to calibrate our estimates: one at 160 (115-308) Mya for the divergence of magnoliids and eudicots (from the TimeTree website: http://www.timetree.org), and 94 Mya as the lower boundary for the *Vitis*-Eurosid split [108].

We also assembled the chloroplast genome of *B. austro-yunnanensis* using GetOrganelle v1.7.2a [109] with cleaned Illumina sequencing reads and obtained annotations using PGA [110]. The chloroplast genes of *B. austro-yunnanensis* and the other published chloroplast genomes were aligned as described above and then concatenated to construct the ML tree with IQ-TREE using the settings “-B 1000 –m MFP”.

### Phylogenetic discordance analyses

Many factors could give rise to incongruent tree topologies among nuclear genes or between nuclear and plastome genes. Here, we used a recently published method [57] (https://github.com/lmcai/Coalescent_simulation_and_gene_flow_detection) to assess the contributions of three factors to gene tree variations: gene tree estimation error, incomplete lineage sorting (ILS), and hybridization (gene flow). In brief, we first calculated bootstrap values for the species tree with all gene trees and used these values to represent the gene tree variation; higher bootstrap values represent lower gene tree variation. For the gene tree estimation error, because there were two species with different topologies in the concatenated and coalescent trees, we first reassessed the branch length with the fixed coalescent topology using IQ-TREE. Next, we used Seq-Gen v1.3.4 [111] to simulate 200 alignments under the GTR model with the rephrased tree and a sequence length of 1500 bp, which is similar to the mean alignment length of our real single copy gene dataset. The parameters of the substitution matrix, base frequency, and gamma rate distribution were extracted from the above IQ-TREE analysis. The simulated alignments were then used to construct gene trees with IQ-TREE as described above, and the gene tree estimation error of each node was quantified in terms of the bootstrap values for the species tree and the simulated gene tree. For ILS, we used the parameter “theta” to represent the probability of ILS in each node; high theta values indicate a large ancestor population size and thus a high ILS level [31, 57]. Theta was calculated on the basis of mutation units inferred by IQ-TREE and coalescent units inferred by ASTRAL. For a rooted three-taxon species tree, there are three possible topologies or triplets: ((A,B),C), ((A,C),B) and ((B,C),A). Two minor discordant triplets will occur at equal frequency under the ILS condition, whereas hybridization will cause their frequencies to differ. Therefore, by applying the chi-squared test to the two minor triplet frequencies in the simulated gene trees and observed trees, one can identify nodes affected by hybridization. We simulated gene trees under the ILS condition with Phybase [112], using the multi-species coalescent model with the coalescent species tree as the input. The reticulation index was then calculated from the frequency of the asymmetrical triplets in all combinations for each node to reflect the hybridization level. Finally, the relative contributions of ILS, estimation error, and gene flow to explaining the gene tree variation were evaluated using linear regression methods as implemented in the R package relaimpo [113]. Besides the above method, we also used the QuIBL to evaluate the hybridization occurrence, which is based on branch length distributions across gene trees to infer putative introgression patterns [58]. To reduce the running time, nine representative species (*Aristolochia*, *Aquilegia*, *Nelumbo*, *Buxus*, *Trochodendron*, *Simmondsia*, *Davidia*, *Cercidiphyllum* and *Vitis*) were selected with *Aristolochia* as the outgroup of the total analysis and the default parameters of QuIBL were used. To distinguish an ILS-only model and a hybridization model, we used the Bayesian Information Criterion (BIC) test with a strict cutoff of ΔBIC > 10.

### WGD analyses

All of the previously mentioned 26 eudicot species were included in the WGD analyses. Synteny blocks and collinear genes within each species and between species were identified using WGDI [114] with the “-icl” parameter setting. Synonymous substitutions per synonymous site (*Ks*) between collinear genes were estimated using the Nei–Gojobori approach as implemented in the PAML package v.4.9h [107]. The median *Ks* values were used to represent each syntenic block, and *Ks* peak fitting was performed with WGDI using “-pf” option. Dot plots of collinear genes and synteny blocks were used to determine syntenic ratios between different species to confirm their polyploidy levels. We also used the collinear genes to perform phylogenetic analyses to determine whether WGD had occurred independently within early-diverging eudicots and core eudicots. Collinear genes between all species pairs from the seven selected species (*Aquilegia*, *Nelumbo*, *Tetracentron*, *Trochodendron*, *Buxus*, *Coffea* and *Vitis*) were extracted using WGDI with the “-at” option, and IQ-TREE was used to construct gene trees as described above. For each gene tree, we randomly rooted a collinear gene from one species using nw_reroot from the Newick utilities v1.6 [115] and then checked to see if retained collinear genes from the other species were clustered as a monophyletic clade using nw_clade (-m), supporting the independent occurrence of WGD in these two species. Finally, we calculated the frequency of gene trees supporting independent WGDs in each species.

### AEK construction and karyotype projection

Six species (*Vitis*, *Trochodendron*, *Tetracentron*, *Buxus*, *Nelumbo* and *Aquilegia*) were selected to represent early-diverging and core eudicots to construct the AEK. WGDI was used to detect collinear genes/blocks between all chromosome pairs among all six species. We firstly generated the dot plots between the all chromosomes and detected many chromosomes showed a nearly intact synteny relationships with others. Then, we used the following method to group these chromosomes and complete the ancestral chromosomes reconstructing. For a pair of chromosomes i and j, we defined the collinearity ratio as ${x}_{ij}=\frac{CN_{\textrm{ij}}\times 2}{N_{\textrm{i}}+{\textrm{N}}_{\textrm{j}}}$, where CN represents the number of collinear genes and N represent the total gene number of the chromosome. The collinearity ratios were then normalized by *Z*-transformation: $Z{x}_{ij}=\frac{x_{ij}-{\mu}_i\ }{\sigma_i}$, where *μ*_*i*_ and *σ*_*i*_ represent the average values and standard deviation of the collinearity ratios between chromosome i and the other chromosomes. The normalized collinearity ratios were used to cluster the chromosomes, which were then displayed using Pheatmap in R. Seven clusters were generated, and we picked the most complete chromosome from each cluster (i.e., the chromosome with the highest collinearity ratio) as a reference. Each reference chromosome was then augmented as follows: if there were no more than five genes in another chromosome between two adjacent collinear genes (located in a single collinear block), we inserted these genes into the reference chromosome between the two collinear genes. After adding all the specific genes from other chromosomes in this way, we obtained the final AEK.

We then used the seven final AEKs to obtain karyotype projections for each eudicot included in the study. As distinguishing the different copies of ancestral chromosomes produced by polyploidization is a difficult and controversial issue, here, we just used the completeness of AEK projection to simply distinguish the different copies and only used it to better display the karyotype evolutionary history. The first, second, and third best completeness copies were marked as A, B, and C, respectively in core eudicots and D and E in *Aquilegia*. The karyotype of core eudicot *Cercidiphyllum* was firstly determined as this species had the simplest karyotype changing (Fig. 4). For other core eudicots, we used *Cercidiphyllum* as reference and if those species having extra duplication events the same method were used to distinguish them.

## Supplementary Information

**Additional file 1: Fig. S1.** The previously reported topologies within eudicots. **Fig. S2.** 19-Kmer-based analysis to estimate the genome size of *Buxus austro-yunnanensis*. **Fig. S3.** Interaction frequency distribution of Hi-C links among chromosomes. **Fig. S4.** GC contents of five early-diverging eudicot species. **Fig. S5.** BUSCO results for six eudicots. **Fig. S6.** LTR insertion time of *Buxus austro-yunnanensis*. **Fig. S7.** Gene structures of *Aquilegia*, *Buxus*, *Nelumbo*, *Trochodendron* and *Tetracentron*. **Fig. S8.** The phylogenetic trees of the nuclear sequences with concatenated and coalescence-based methods. **Fig. S9.** Divergence times of the 28 species. **Fig. S10.** The phylogenetic tree of the chloroplast dataset. **Fig. S11.** Quartet score of each node based on the nuclear gene trees. **Fig. S12.** Superimposed ultrametric gene trees in a consensus DensiTree plot. **Fig. S13.** Relative importance of incomplete lineage sorting (ILS), gene tree estimation error (Est. error), and hybridization in generating gene tree variation. **Fig. S14.** Collinear gene dot plots between *Buxus austro-yunnanensis* and *Aristolochia*, *Aquilegia*, *Nelumbo*, *Tetracentron*, *Cercidiphyllum*, *Vitis*. **Fig. S15.** Collinear gene dot plots between *Cercidiphyllum*, *Vitis* and *Simmondsia*, *Carthamus*, *Olea*. **Fig. S16.** Merged dotplot of *Aquilegia*, *Buxus*, *Nelumbo*, *Trochodendron*, *Tetracentron* and *Vitis*. **Fig. S17.** Heatmap of cluster of collinearity relationships in *Aquilegia* (aco), *Buxus austro-yunnanensis* (byu), *Nelumbo* (nnu), *Trochodendron* (tar), *Tetracentron* (tsi) and *Vitis* (vvi). **Fig. S18.** Demonstration of pieces of AEK 1 in *Aquilegia* (aco), *Buxus austro-yunnanensis* (byu), *Nelumbo* (nnu), *Trochodendron* (tar), *Tetracentron* (tsi) and *Vitis* (vvi). **Fig. S19.** Demonstration of pieces of AEK 2 in *Aquilegia* (aco), *Buxus austro-yunnanensis* (byu), *Nelumbo* (nnu), *Trochodendron* (tar), *Tetracentron* (tsi) and *Vitis* (vvi). **Fig. S20.** Demonstration of pieces of AEK 3 in *Aquilegia* (aco), *Buxus austro-yunnanensis* (byu), *Nelumbo* (nnu), *Trochodendron* (tar), *Tetracentron* (tsi) and *Vitis* (vvi). **Fig. S21.** Demonstration of pieces of AEK 4 in *Aquilegia* (aco), *Buxus austro-yunnanensis* (byu), *Nelumbo* (nnu), *Trochodendron* (tar), *Tetracentron* (tsi) and *Vitis* (vvi). **Fig. S22.** Demonstration of pieces of AEK 5 in *Aquilegia* (aco), *Buxus austro-yunnanensis* (byu), *Nelumbo* (nnu), *Trochodendron* (tar), *Tetracentron* (tsi) and *Vitis* (vvi). **Fig. S23.** Demonstration of pieces of AEK 6 in *Aquilegia* (aco), *Buxus austro-yunnanensis* (byu), *Nelumbo* (nnu), *Trochodendron* (tar), *Tetracentron* (tsi) and *Vitis* (vvi). **Fig. S24.** Demonstration of pieces of AEK 7 in *Aquilegia* (aco), *Buxus austro-yunnanensis* (byu), *Nelumbo* (nnu), *Trochodendron* (tar), *Tetracentron* (tsi) and *Vitis* (vvi). **Fig. S25.** Comparison between the previous AEK (PAEK) and our constructed AEK. **Fig. S26.** Karyotype projection of *Aquilegia*, *Nelumbo*, *Buxus austro-yunnanensis*, *Tetracentron*, *Trochodendron* and *Coffea* genome based on PAEK. **Fig. S27.** Karyotype projection of *Vitis* and *Cercidiphyllum* based on PAEK and AEK. **Fig. S28.** Karyotype projection of *Aquilegia*, *Nelumbo*, *Buxus austro-yunnanensis*, *Tetracentron*, *Trochodendron* and *Coffea* genome based on AEK. **Fig. S29.** Collinear gene dot plots and karyotype projection between *Cercidiphyllum* and *Bretschneidera*, *Theobroma*, *Acer*, *Averrhoa*, *Populus*, *Tripterygium*. **Fig. S30.** Collinear gene dot plots and karyotype projection between *Cercidiphyllum* and *Cucumis*, *Phaseolus*, *Malus*, *Betula*, *Eucalyptus*, *Vitis*. **Fig. S31.** Collinear gene dot plots and karyotype projection between *Cercidiphyllum* and *Coffea*, *Ipomoea*, *Olea*, *Apium*, *Carthamus*, *Davidia*, *Rhododendron*, *Simmondsia*. **Fig. S32.** Collinear gene dot plots and karyotype projection between *Coptis* and *Aquilegia*, *Cercidiphyllum* and *Muscadinia*, *Vitis* and *Muscadinia*. **Fig. S33.** Collinear gene dot plots and karyotype projection between *Tetracentron* and *Trochodendron*. **Fig. S34.** Synteny comparison among asterids species with *Davidia* as reference. **Fig. S35.** Synteny comparison among rosids species with *Tripterygium* as reference. **Table S1.** The detail sequencing information of *Buxus austro-yunnanensis*. **Table S2.** Summary of genome assembly. **Table S3.** Contig N50 values of the early-diverging eudicots. **Table S4.** Summary of chromosome level assembly. **Table S5.** Statistic of repetitive elements in the assembled genome. **Table S6.** Comparison of gene space of *Buxus austro-yunnanensis* with other genomes. **Table S7.** Assessment of the predicted genes by BUSCO (database: eudicotyledons_odb10). **Table S8.** Functional annotation of the predicted genes. **Table S9.** Summary of the 28 species genome that used in this study. **Table S10.** Average total introgression proportion per species pair in QuIBL analysis. **Table S11.** The QuIBL analysis result.

## Data Availability

All raw sequence reads used in this study have been deposited at NCBI under the BioProject accession number PRJNA808839 [116]. We also uploaded the assembly and annotation files in the Genome Warehouse in BIG Data Center under the BioProject accession number PRJCA008395 [117]. The reconstructed AEK files and projection files of eudicots are available at FigShare (10.6084/m9.figshare.19243605.v1 for AEK [118], 10.6084/m9.figshare.21091702.v1 for projection files [119]).
