# Supplementary material for: A high-quality Buxus austro-yunnanensis (Buxales) genome provides new insights into karyotype evolution in early eudicots
Source: BMC Biol. 2022 Oct 4;20:216. doi: 10.1186/s12915-022-01420-1 (PMC9533543; doi:10.1186/s12915-022-01420-1)
Supplement: Supplementary file 1 — Additional file 1: Fig. S1. The previously reported topologies within eudicots. Fig. S2. 19-Kmer-based analysis to estimate the genome size of Buxus austro-yunnanensis. Fig. S3. Interaction frequency distribution of Hi-C links among chromosomes. Fig. S4. GC contents of five early-diverging eudicot species. Fig. S5. BUSCO results for six eudicots. Fig. S6. LTR insertion time of Buxus austro-yunnanensis. Fig. S7. Gene structures of Aquilegia, Buxus, Nelumbo, Trochodendron and Tetracentron. Fig. S8. The phylogenetic trees of the nuclear sequences with concatenated and coalescence-based methods. Fig. S9. Divergence times of the 28 species. Fig. S10. The phylogenetic tree of the chloroplast dataset. Fig. S11. Quartet score of each node based on the nuclear gene trees. Fig. S12. Superimposed ultrametric gene trees in a consensus DensiTree plot. Fig. S13. Relative importance of incomplete lineage sorting (ILS), gene tree estimation error (Est. error), and hybridization in generating gene tree variation. Fig. S14. Collinear gene dot plots between Buxus austro-yunnanensis and Aristolochia, Aquilegia, Nelumbo, Tetracentron, Cercidiphyllum, Vitis. Fig. S15. Collinear gene dot plots between Cercidiphyllum, Vitis and Simmondsia, Carthamus, Olea. Fig. S16. Merged dotplot of Aquilegia, Buxus, Nelumbo, Trochodendron, Tetracentron and Vitis. Fig. S17. Heatmap of cluster of collinearity relationships in Aquilegia (aco), Buxus austro-yunnanensis (byu), Nelumbo (nnu), Trochodendron (tar), Tetracentron (tsi) and Vitis (vvi). Fig. S18. Demonstration of pieces of AEK 1 in Aquilegia (aco), Buxus austro-yunnanensis (byu), Nelumbo (nnu), Trochodendron (tar), Tetracentron (tsi) and Vitis (vvi). Fig. S19. Demonstration of pieces of AEK 2 in Aquilegia (aco), Buxus austro-yunnanensis (byu), Nelumbo (nnu), Trochodendron (tar), Tetracentron (tsi) and Vitis (vvi). Fig. S20. Demonstration of pieces of AEK 3 in Aquilegia (aco), Buxus austro-yunnanensis (byu), Nelumbo (nnu), Trochodendron (tar), Tetra [file 12915_2022_1420_MOESM1_ESM.docx]

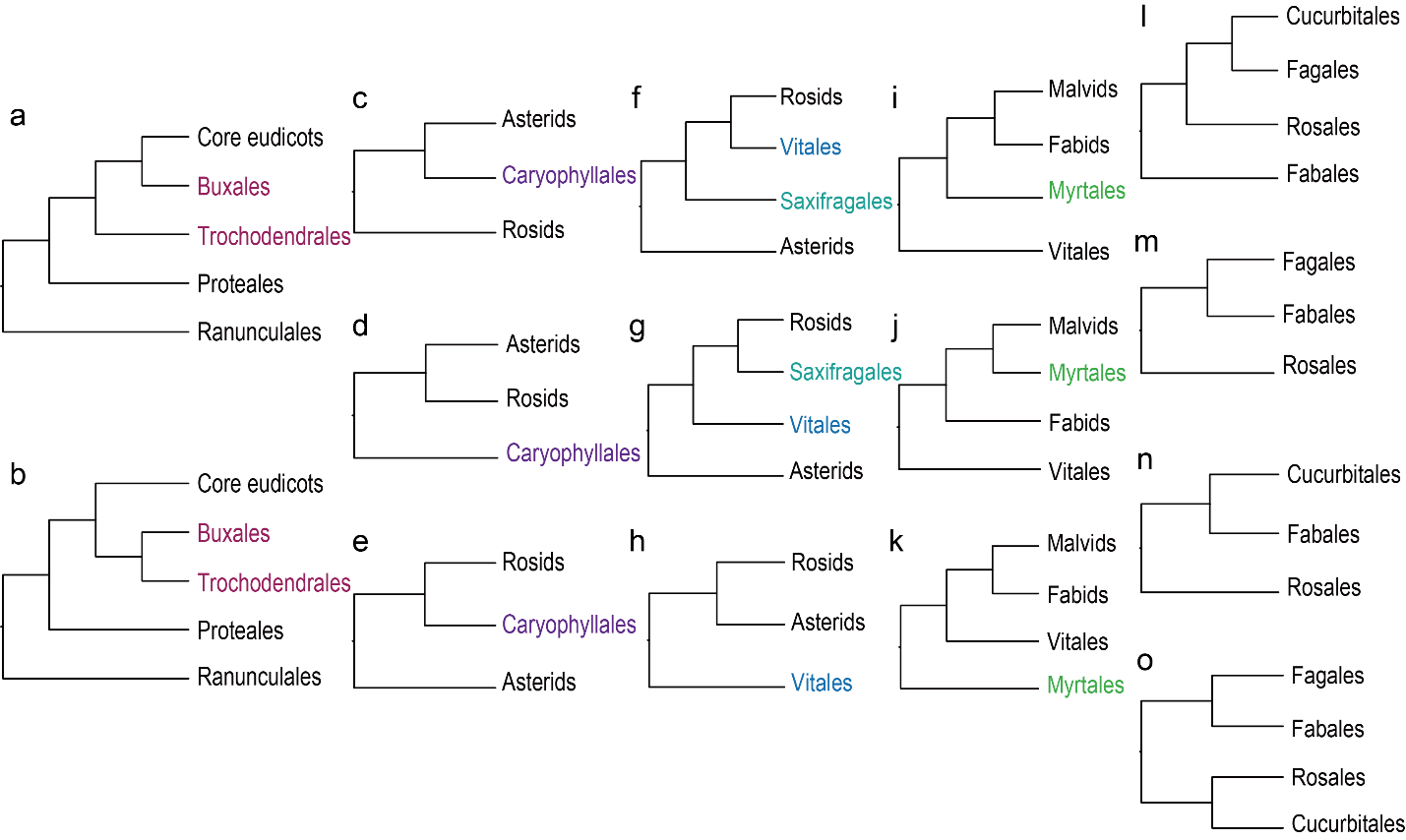


## Fig. S1. The previously reported topologies within eudicots. (a) refs [3, 28]; (b) ref [27]; (c) refs [3, 27, 28, 33–38]; (d) refs [39–45]; (e) ref [46]; (f) refs [3, 28, 33]; (g) refs [27, 36, 37]; (h) ref [47]; (i) refs [33, 37, 45–53]; (j) refs [3, 27, 28]; (k) ref [43]; (l) refs [3, 28]; (m) ref [27]; (n) ref [46]; (o) refs [43, 50].


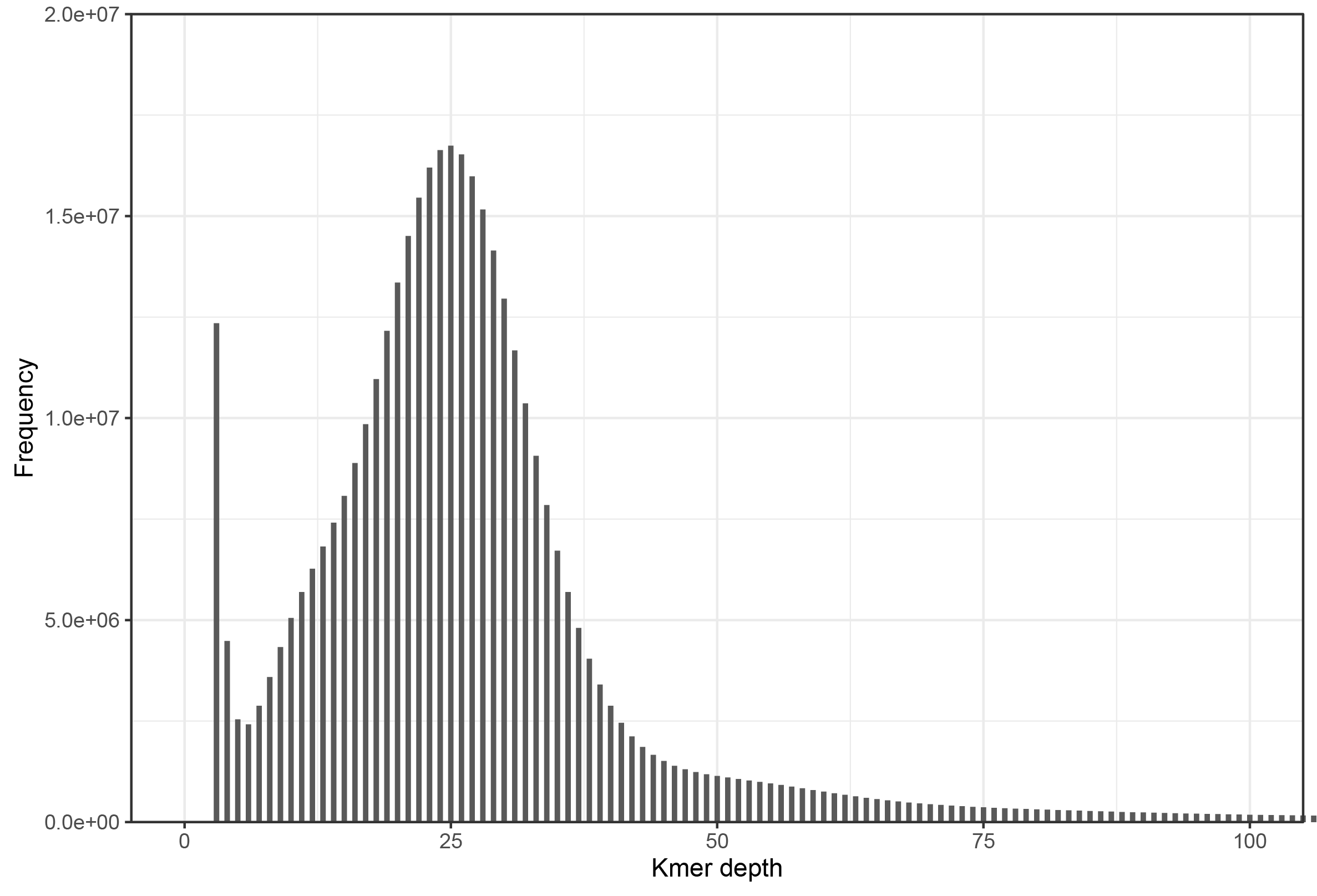


## Fig. S2. 19-Kmer-based analysis to estimate the genome size of *Buxus austro-yunnanensis*.


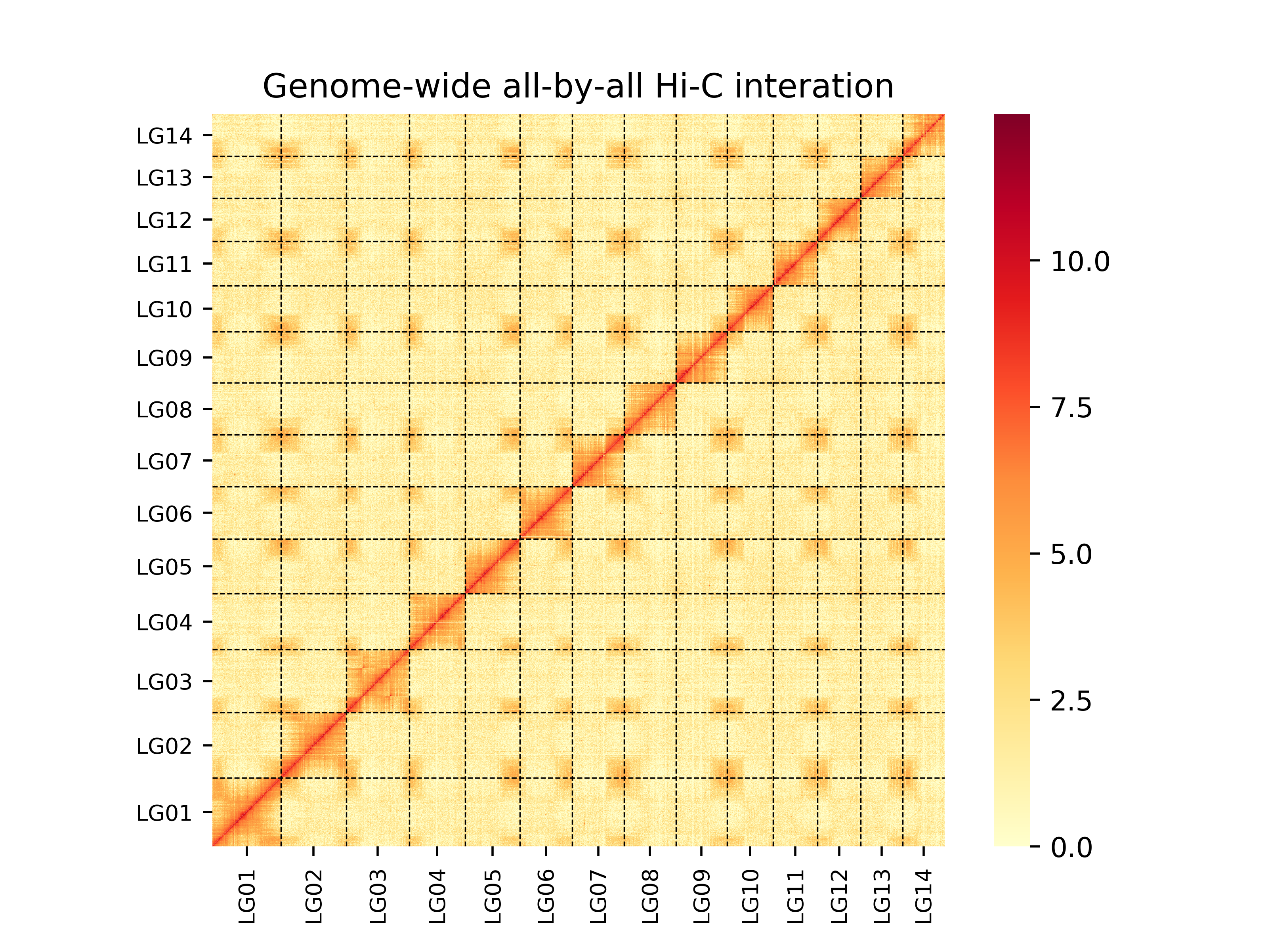


## Fig. S3. Interaction frequency distribution of Hi-C links among chromosomes.


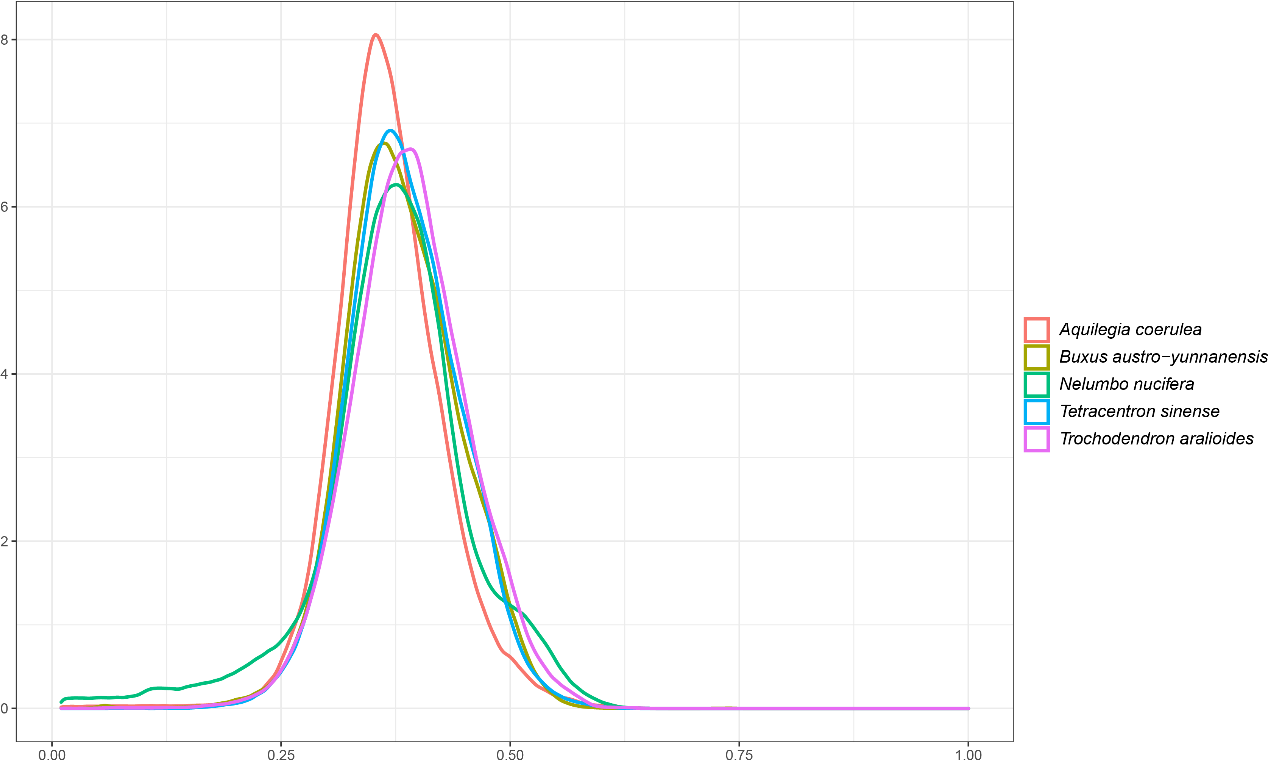


## Fig. S4. GC contents of five early-diverging eudicot species.


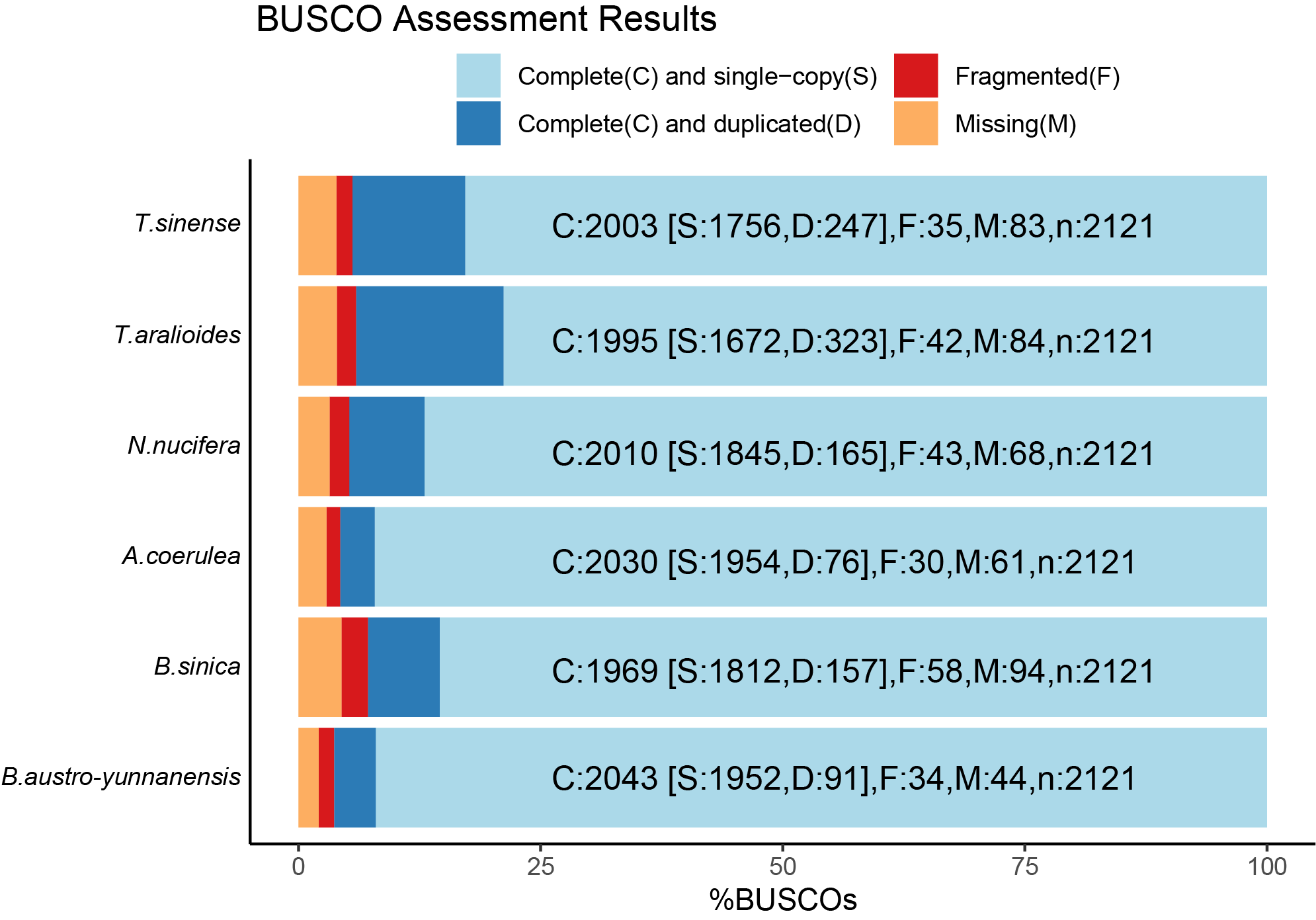


## **Fig. S5. BUSCO results for six eudicots.** The BUSCO dataset Eudicotyledons v10 was selected to evaluate all genomes.


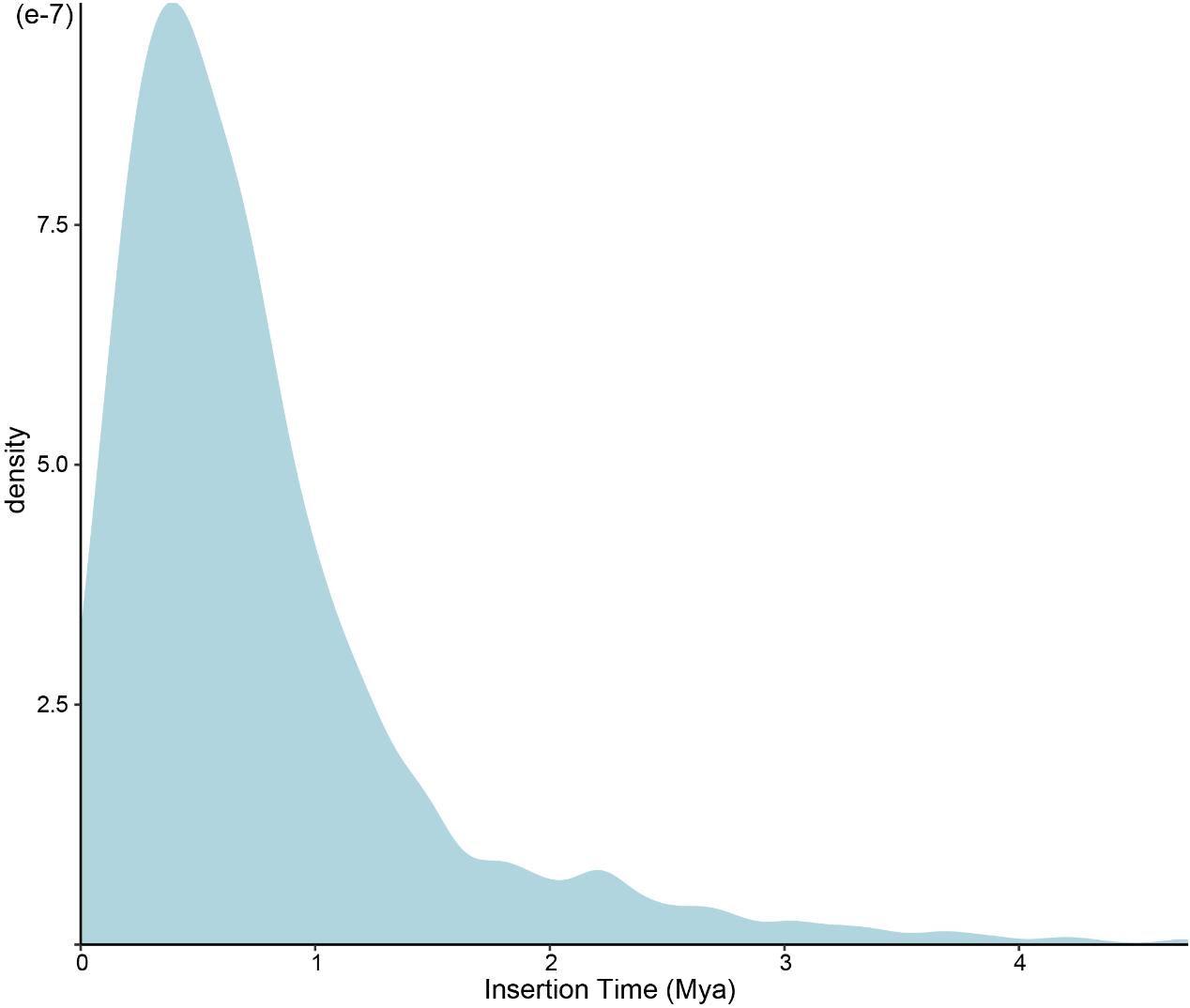


## Fig. S6. LTR insertion time of *Buxus austro-yunnanensis*.


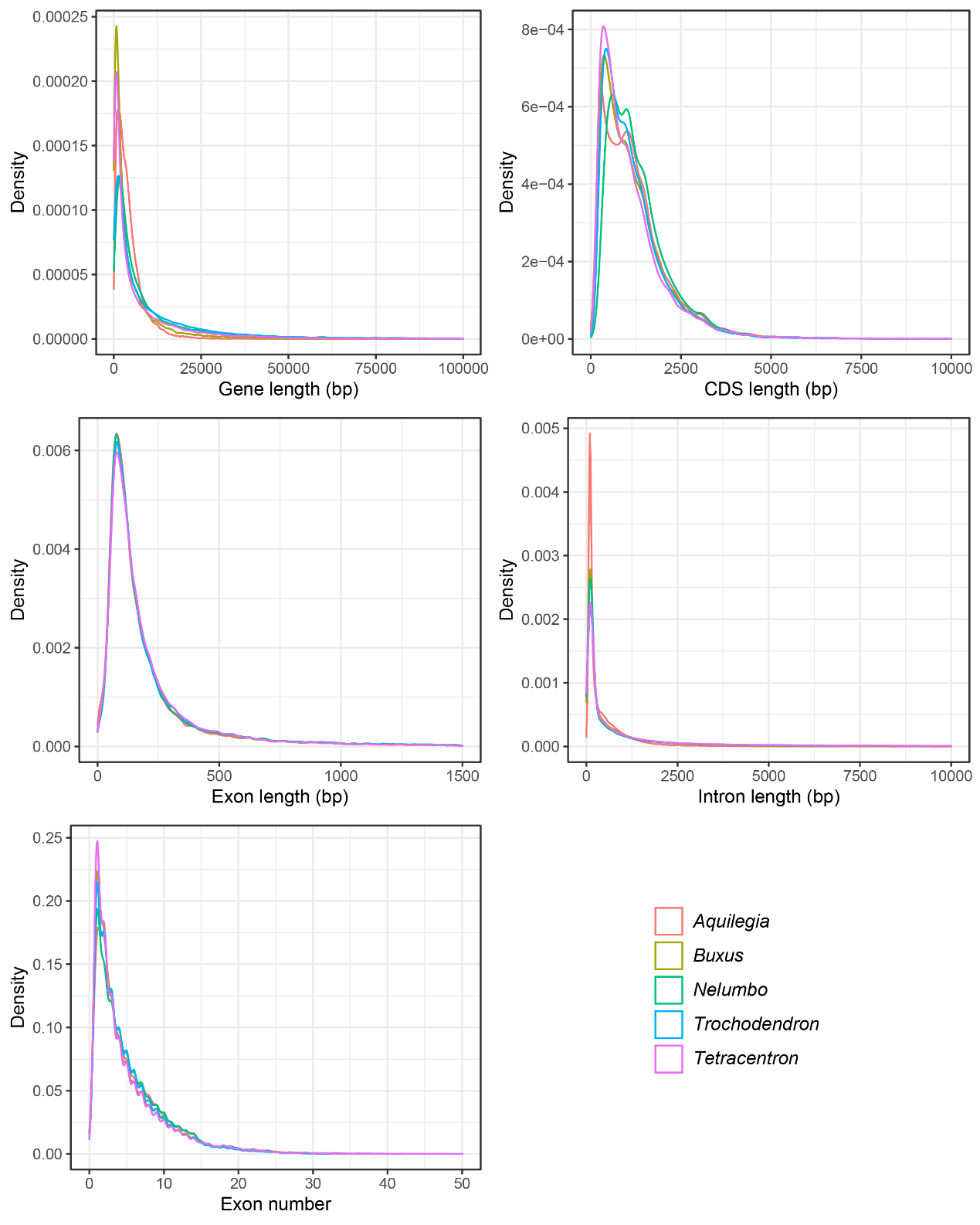


## Fig. S7. Gene structures of *Aquilegia, Buxus, Nelumbo, Trochodendron* and *Tetracentron*.


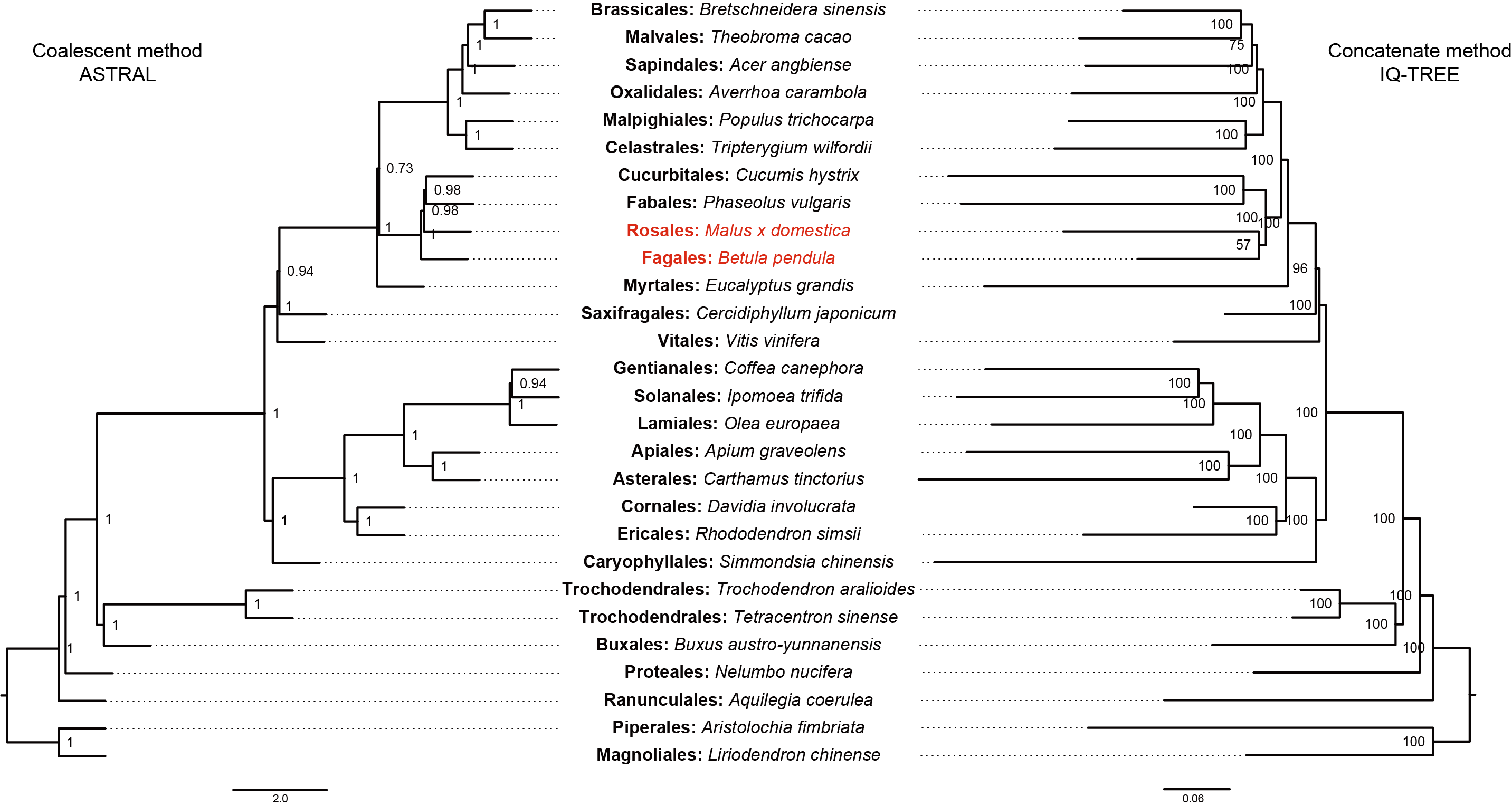


## Fig. S8. The phylogenetic trees of the nuclear sequences with concatenated and coalescence-based methods.


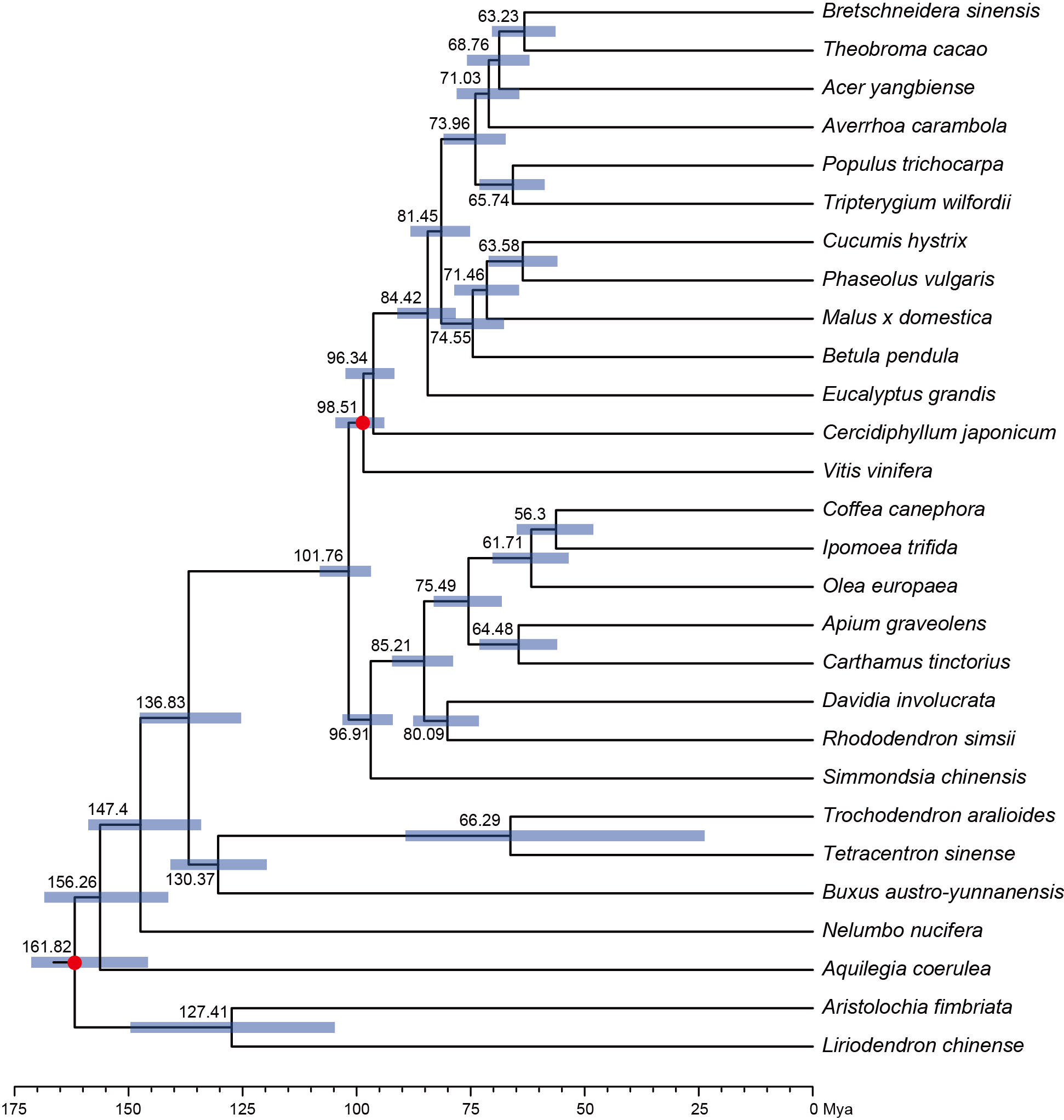


## Fig. S9. Divergence times of the 28 species. Divergence time (Mya, million years ago) were indicated above nodes and the blue nodal bars show 95% confidence intervals. The red dots correspond to calibration points as described in the methods section.


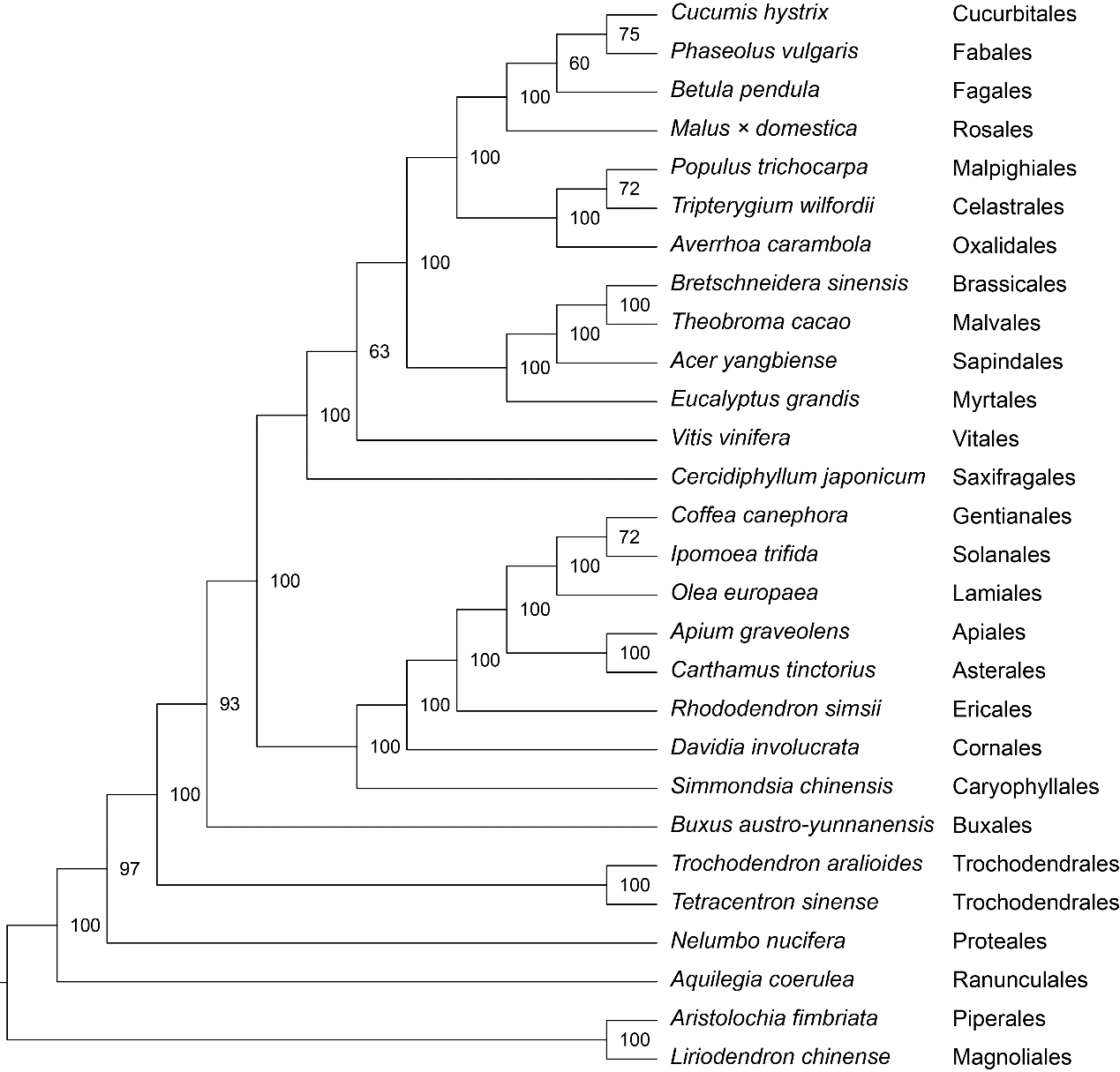


## Fig. S10. The phylogenetic tree of the chloroplast dataset.


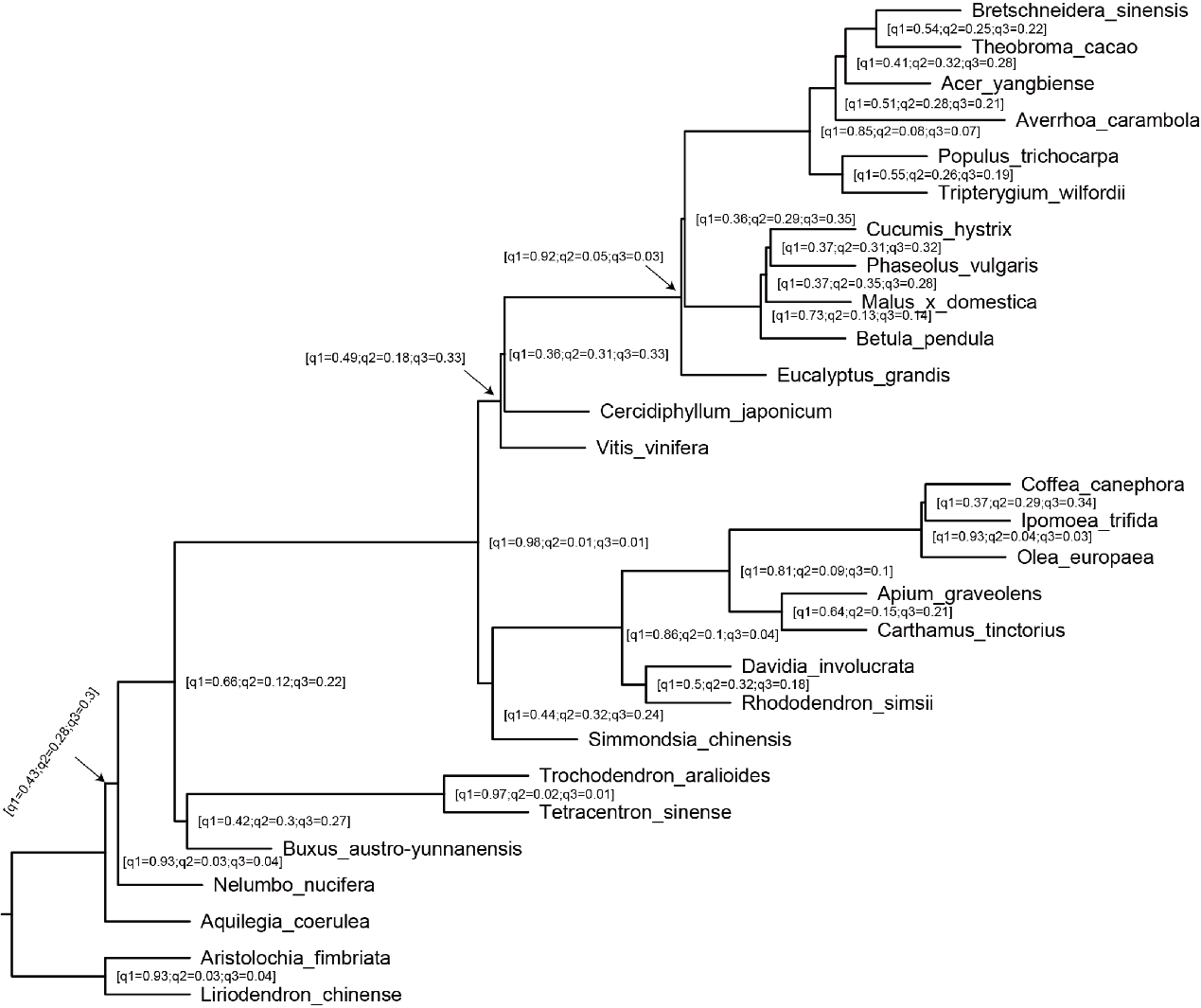


## Fig. S11. Quartet score of each node based on the nuclear gene trees.


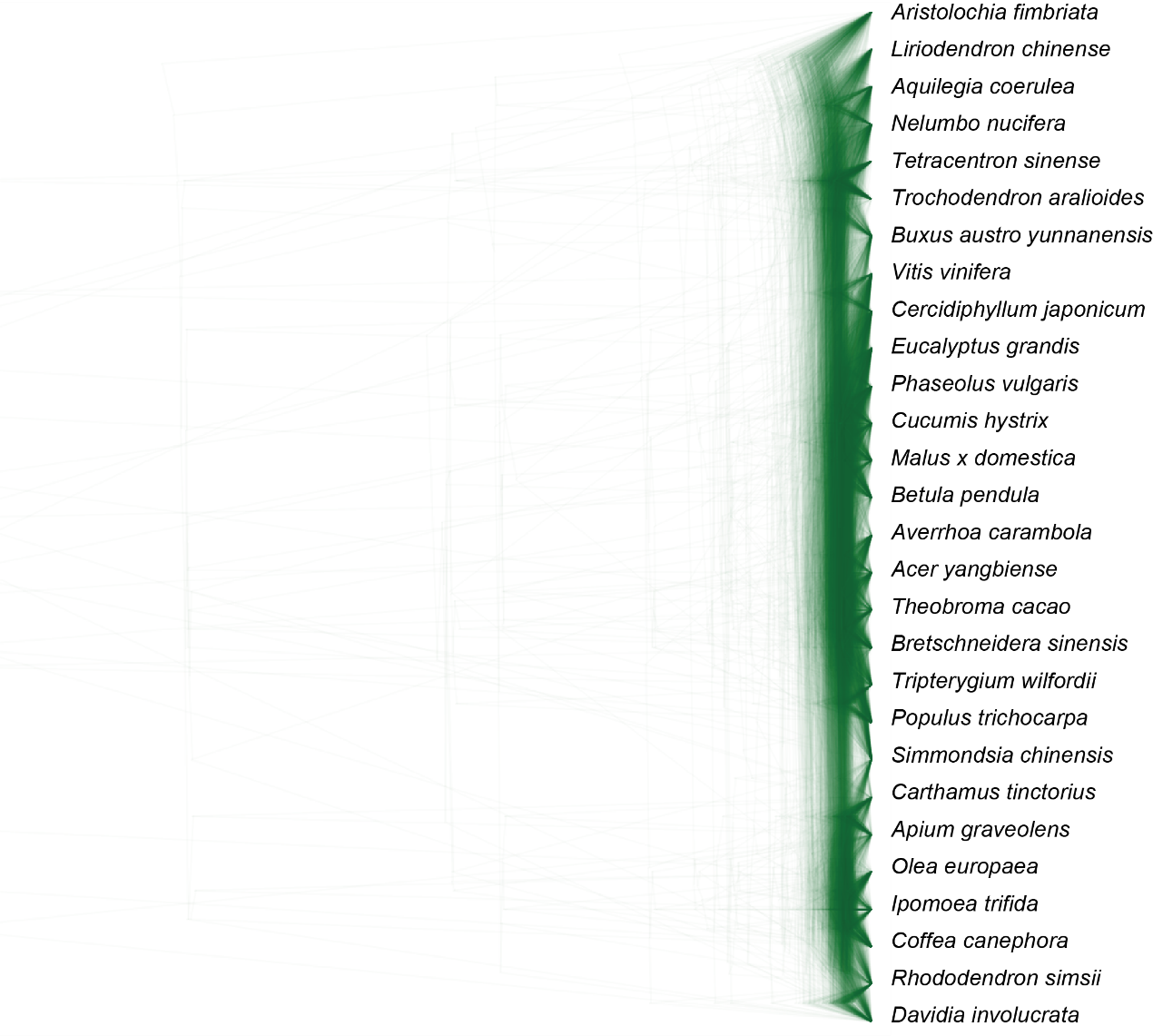


## Fig. S12. Superimposed ultrametric gene trees in a consensus DensiTree plot.

##
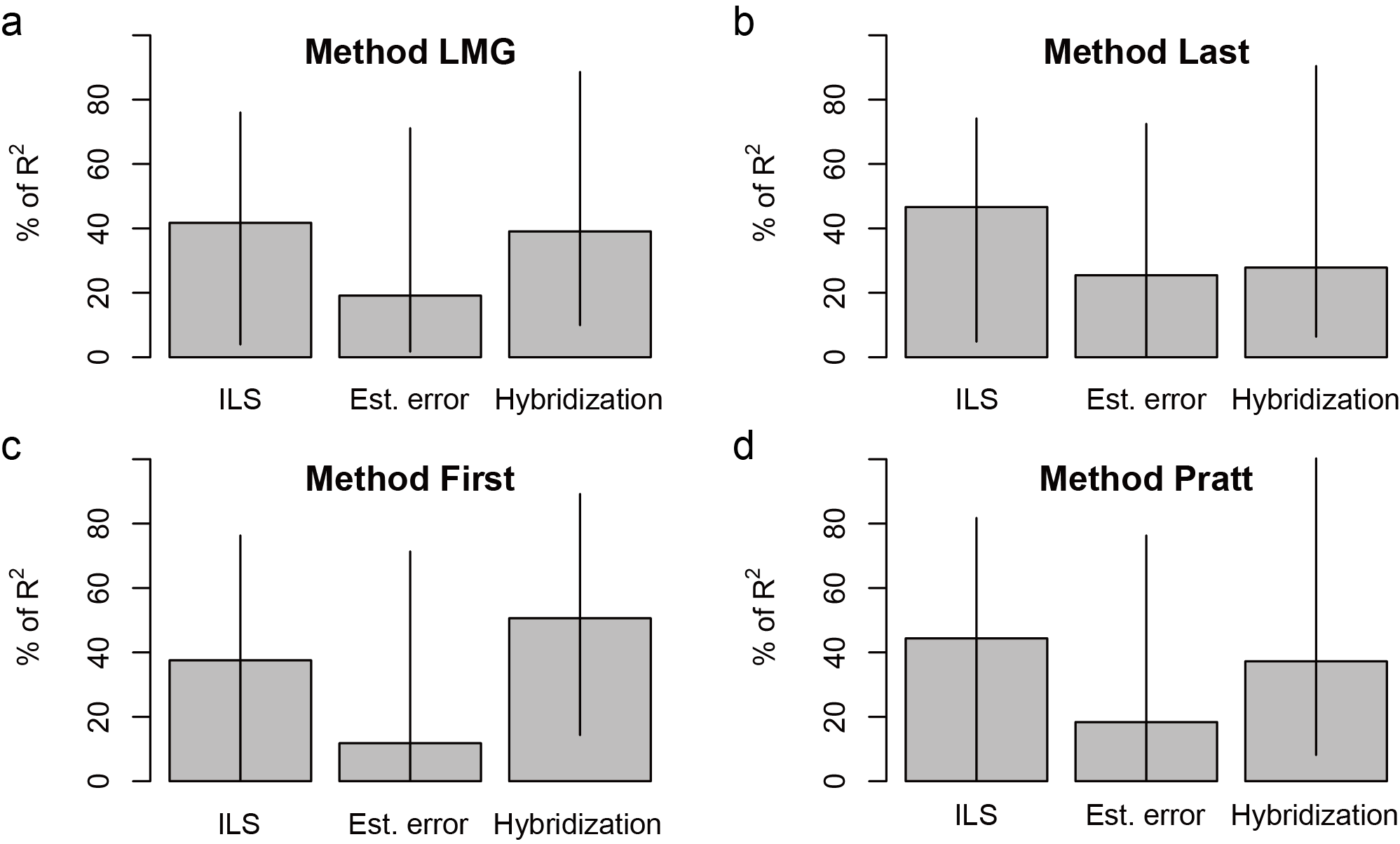
Fig. S13. Relative importance of incomplete lineage sorting (ILS), gene tree estimation error (Est. error), and hybridization in generating gene tree variation. The percentages are estimated based on four regression methods (LMG, Last, First, and Pratt) implemented in the R package relaimpo. 95% confidence intervals are represented by bars. The total R2=42.45% and the metrics are normalized to sum 100%.


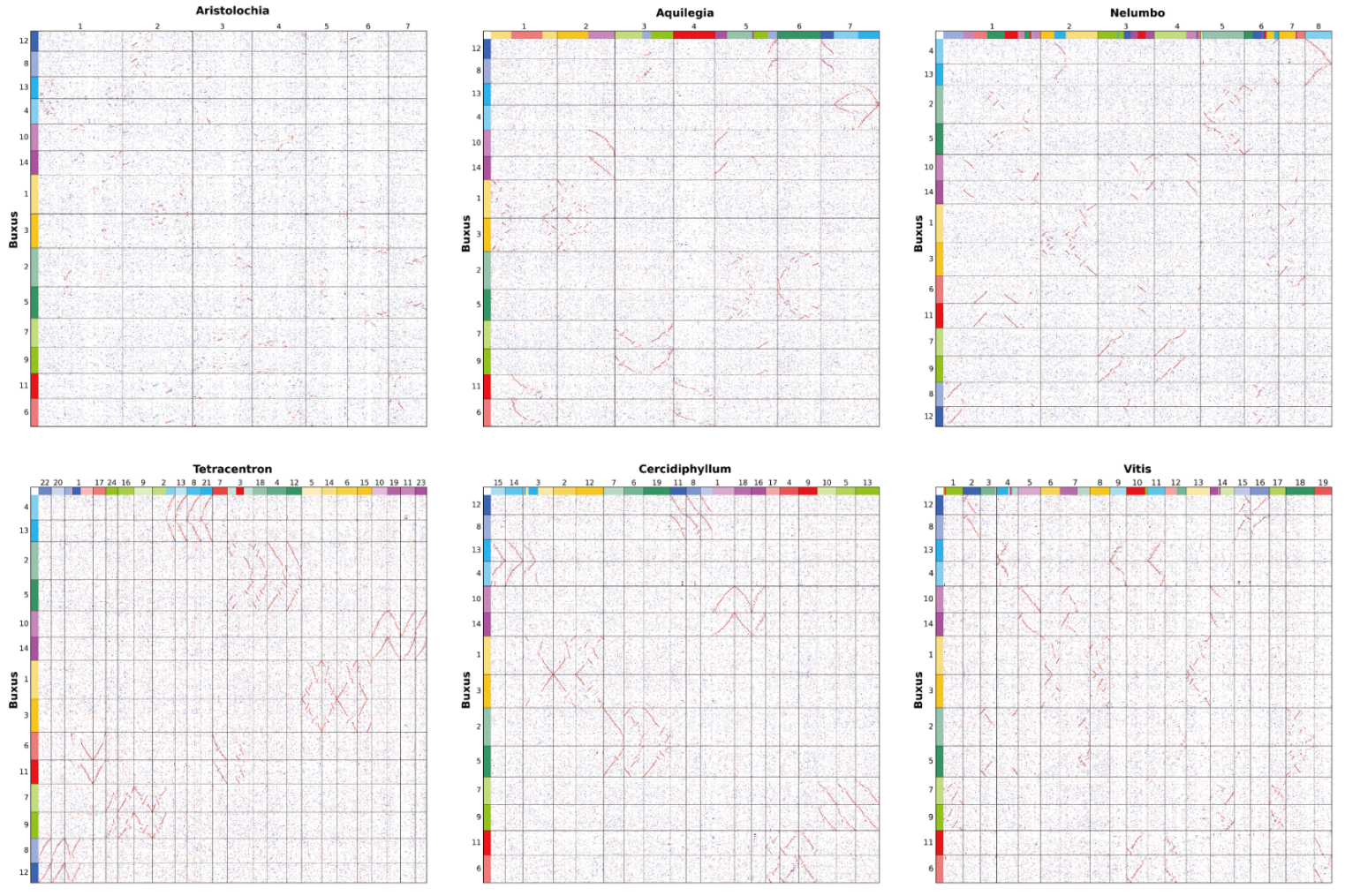


## Fig. S14. Collinear gene dot plots between *Buxus austro-yunnanensis* and *Aristolochia, Aquilegia, Nelumbo, Tetracentron, Cercidiphyllum, Vitis*.

##
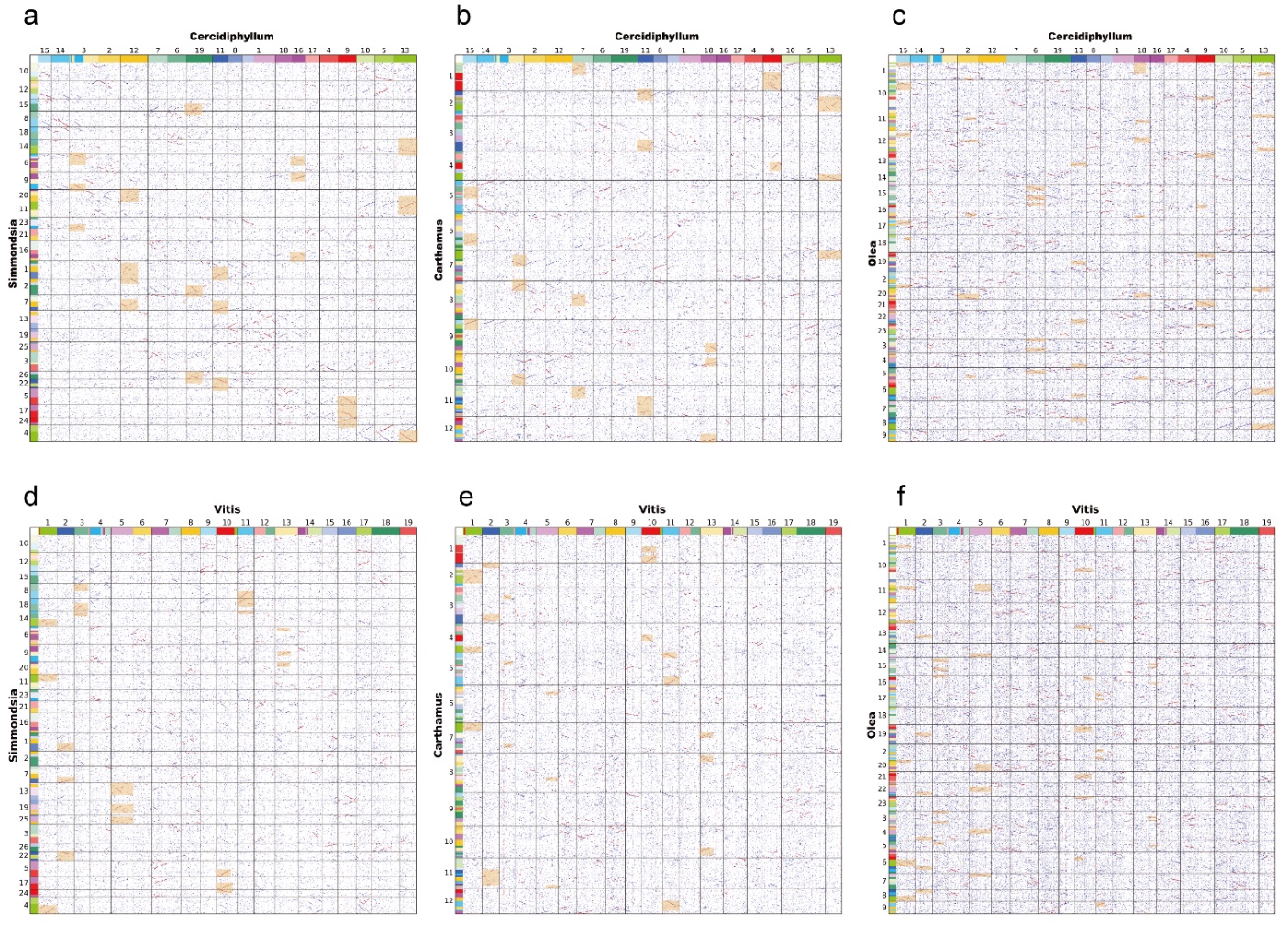
Fig. S15. Collinear gene dot plots between *Cercidiphyllum*, *Vitis* and *Simmondsia, Carthamus*, *Olea*. Yellow panes highlighted collinearity of the best match of the chromosomes in *Cercidiphyllum* and *Vitis* with clearly ratio of 3:1, 3:1 and 6:1 (consider only one copy in *Cercidiphyllum* and *Vitis*).

**
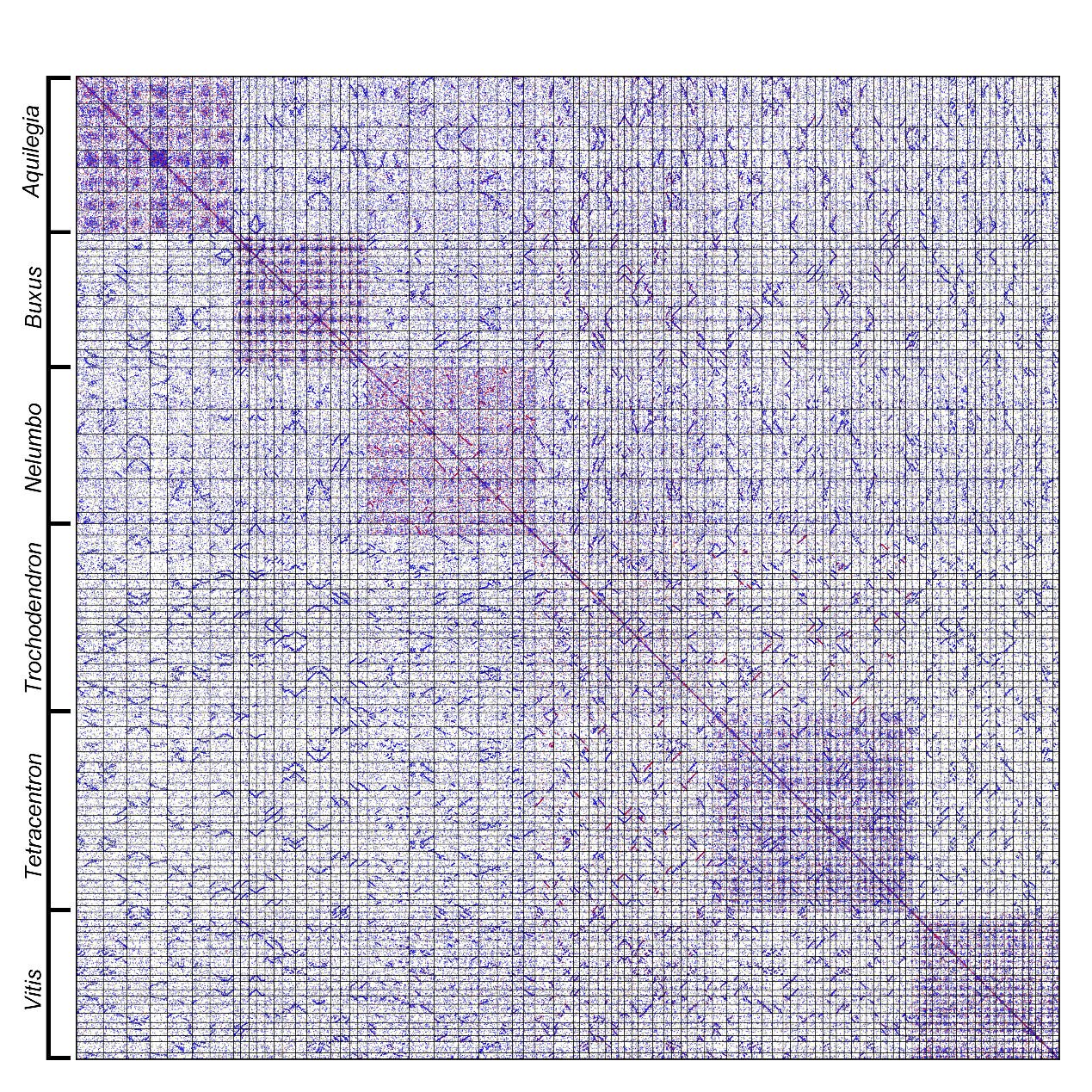
**

**Fig. S16. Merged dotplot of *Aquilegia*, *Buxus*, *Nelumbo*, *Trochodendron*, *Tetracentron* and *Vitis.***

**
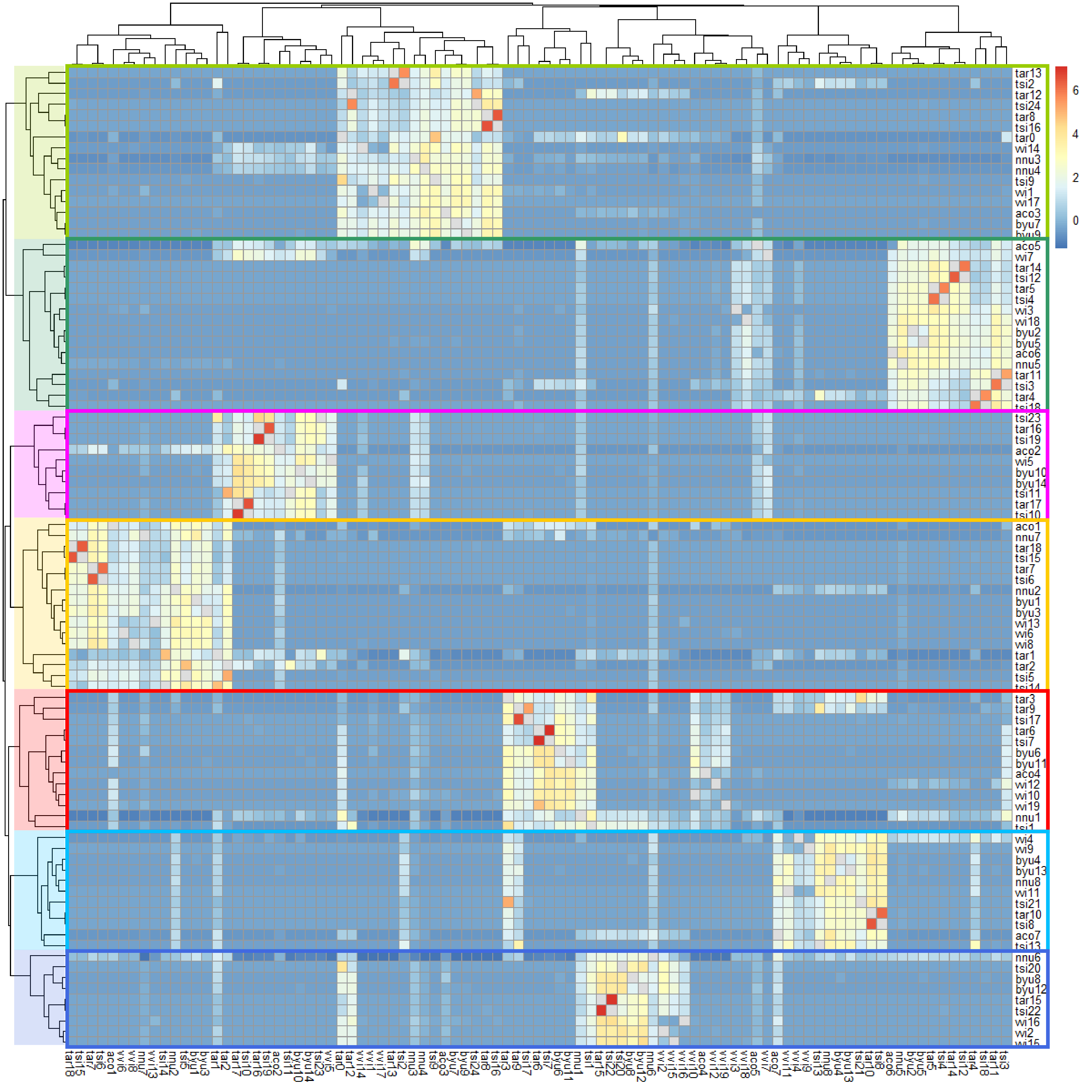
**

## Fig. S17. Heatmap of cluster of collinearity relationships in *Aquilegia* (aco), *Buxus austro-yunnanensis* (byu), *Nelumbo* (nnu)*, Trochodendron* (tar)*, Tetracentron* (tsi) and *Vitis* (vvi).

**
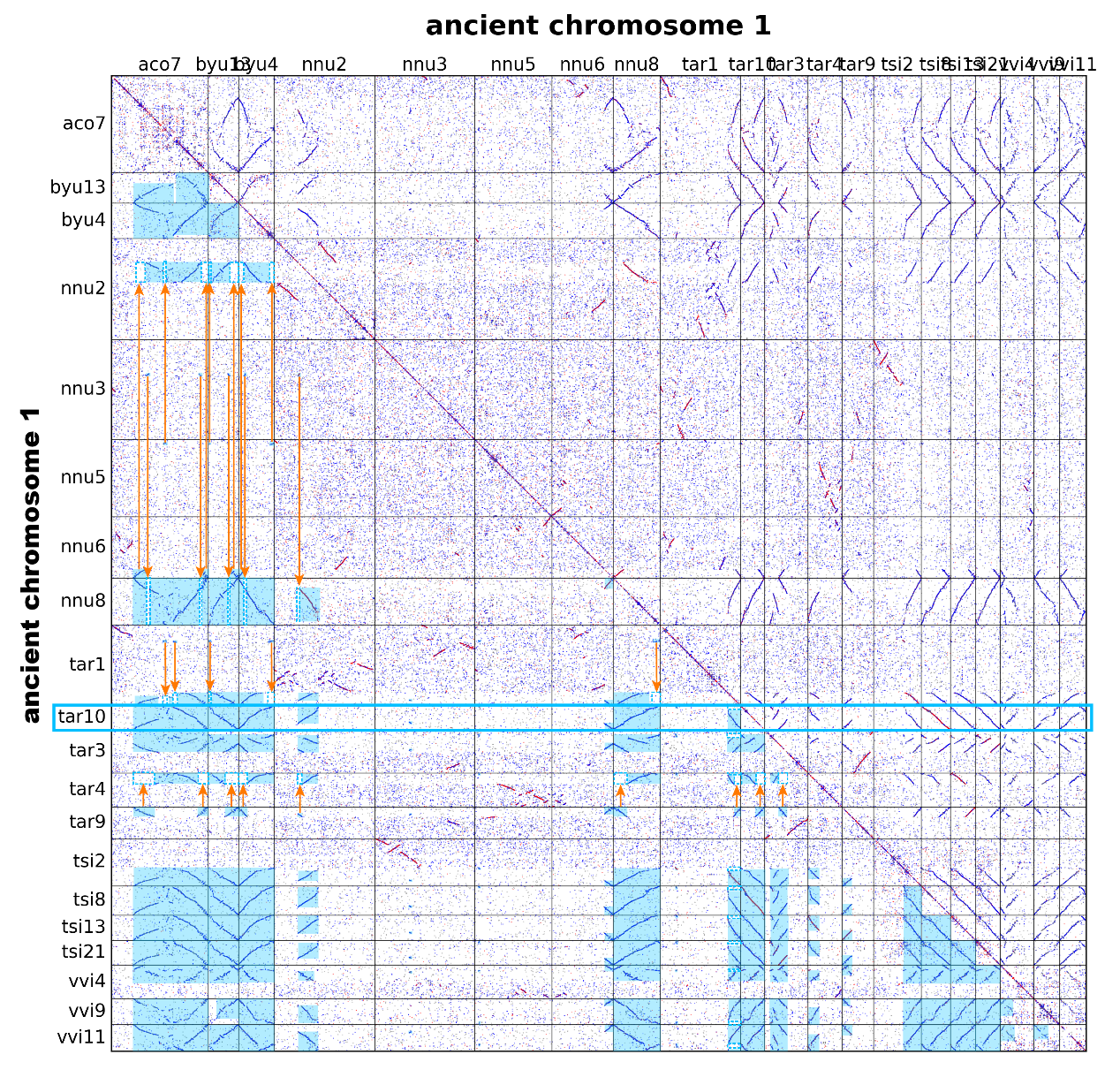
**

## Fig. S18. Demonstration of pieces of AEK 1 in *Aquilegia* (aco), *Buxus austro-yunnanensis* (byu), *Nelumbo* (nnu), *Trochodendron* (tar), *Tetracentron* (tsi) and *Vitis* (vvi). Arrows mean those two pieces belong to one ancient chromosome; chromosome highlighted by box was used as reference chromosome in AEK construction.

**
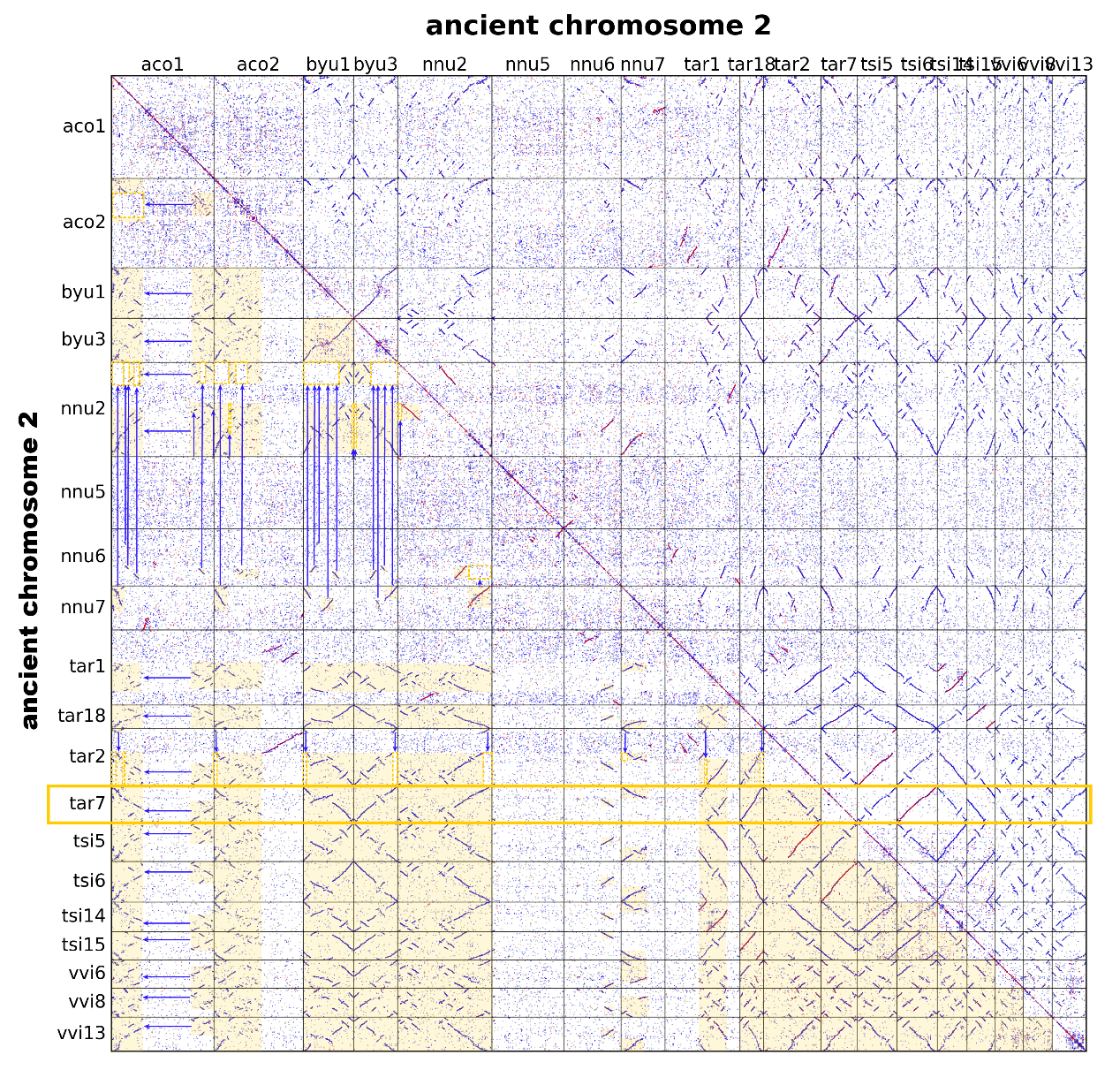
**

## Fig. S19. Demonstration of pieces of AEK 2 in *Aquilegia* (aco), *Buxus austro-yunnanensis* (byu), *Nelumbo* (nnu), *Trochodendron* (tar), *Tetracentron* (tsi) and *Vitis* (vvi). Arrows mean those two pieces belong to one ancient chromosome; chromosome highlighted by box was used as reference chromosome in AEK construction.

**
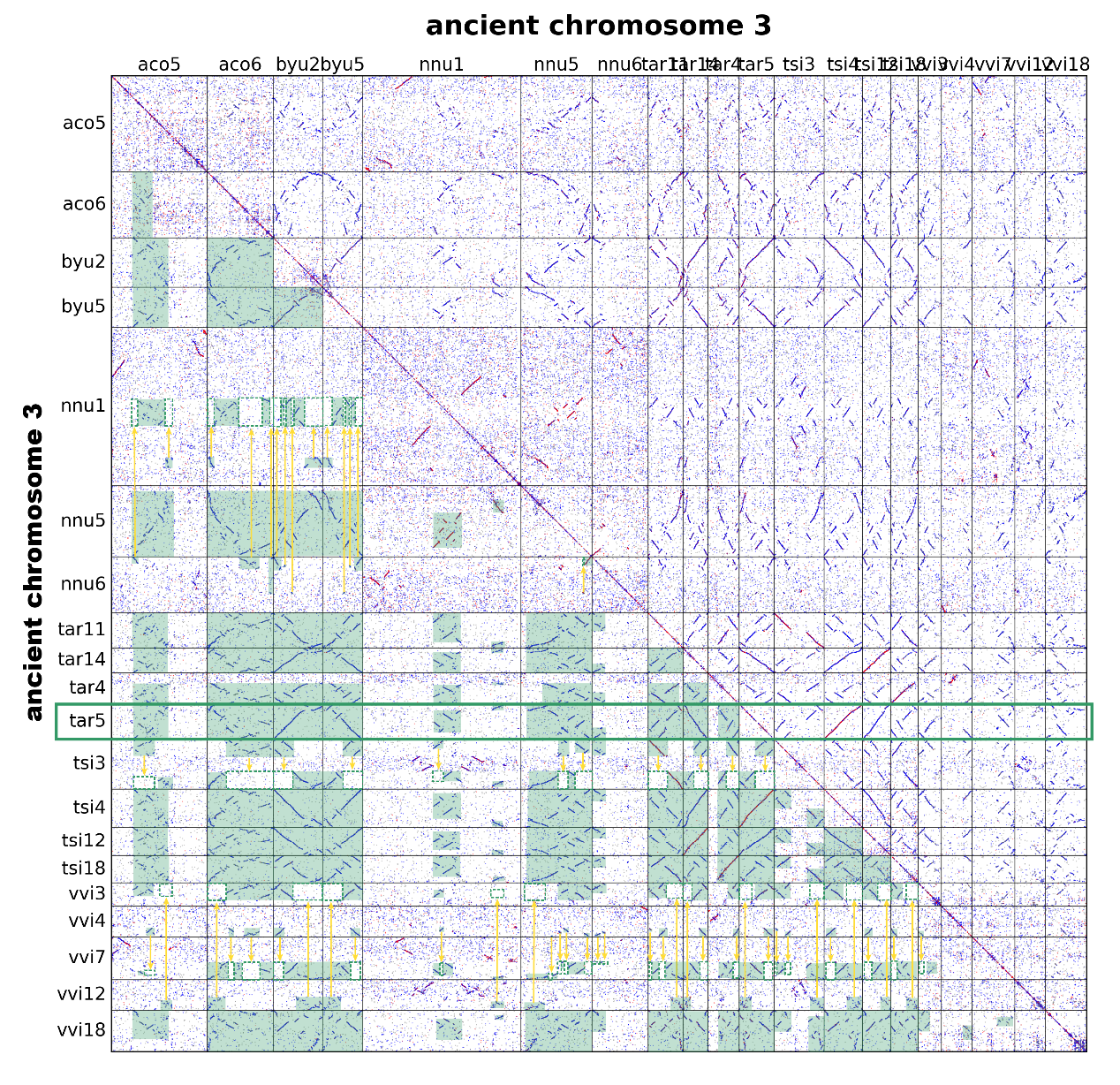
**

## Fig. S20. Demonstration of pieces of AEK 3 in *Aquilegia* (aco), *Buxus austro-yunnanensis* (byu), *Nelumbo* (nnu), *Trochodendron* (tar), *Tetracentron* (tsi) and *Vitis* (vvi). Arrows mean those two pieces belong to one ancient chromosome; chromosome highlighted by box was used as reference chromosome in AEK construction.

**
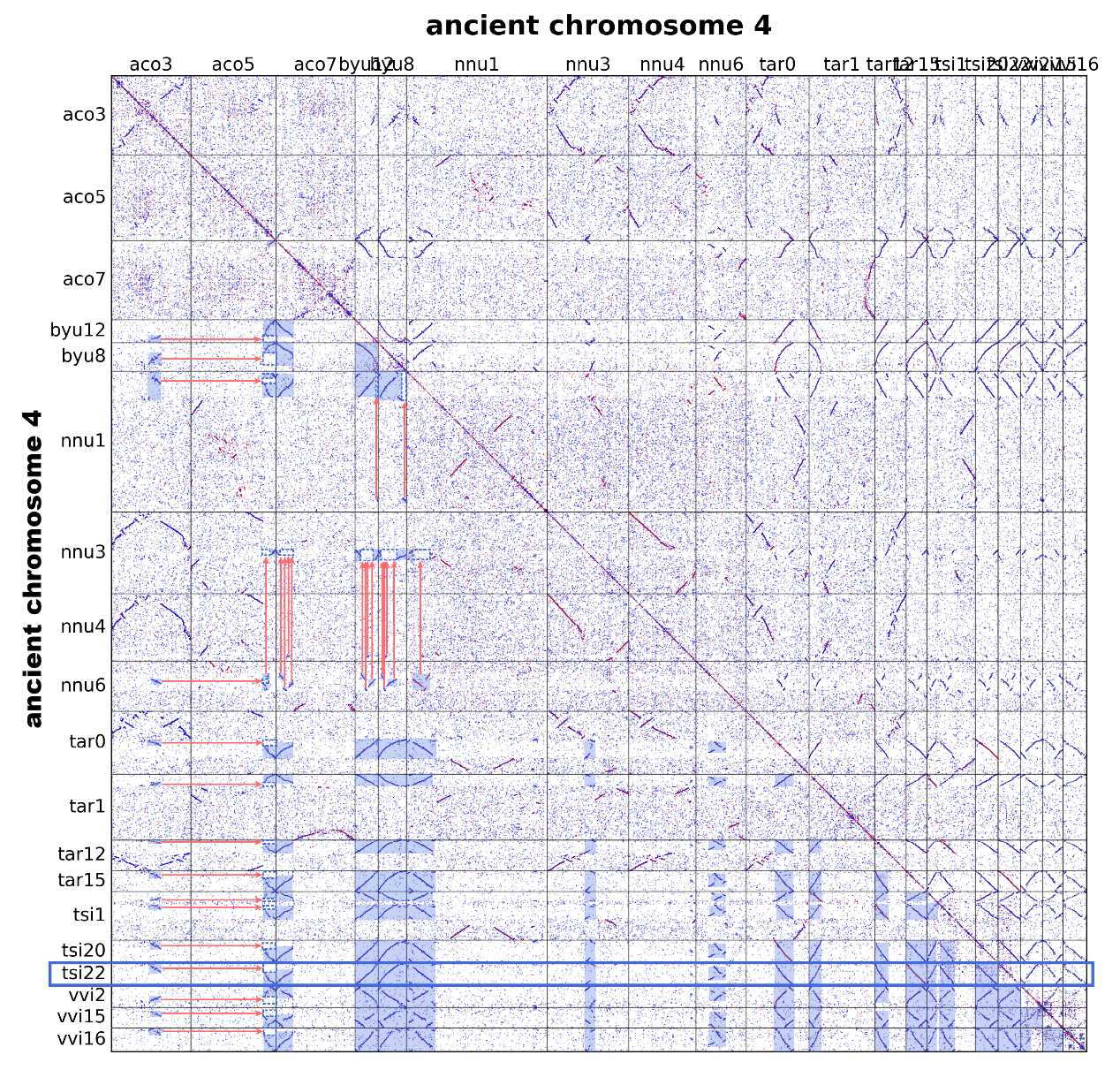
**

## Fig. S21. Demonstration of pieces of AEK 4 in *Aquilegia* (aco), *Buxus austro-yunnanensis* (byu), *Nelumbo* (nnu), *Trochodendron* (tar), *Tetracentron* (tsi) and *Vitis* (vvi). Arrows mean those two pieces belong to one ancient chromosome; chromosome highlighted by box was used as reference chromosome in AEK construction.

**
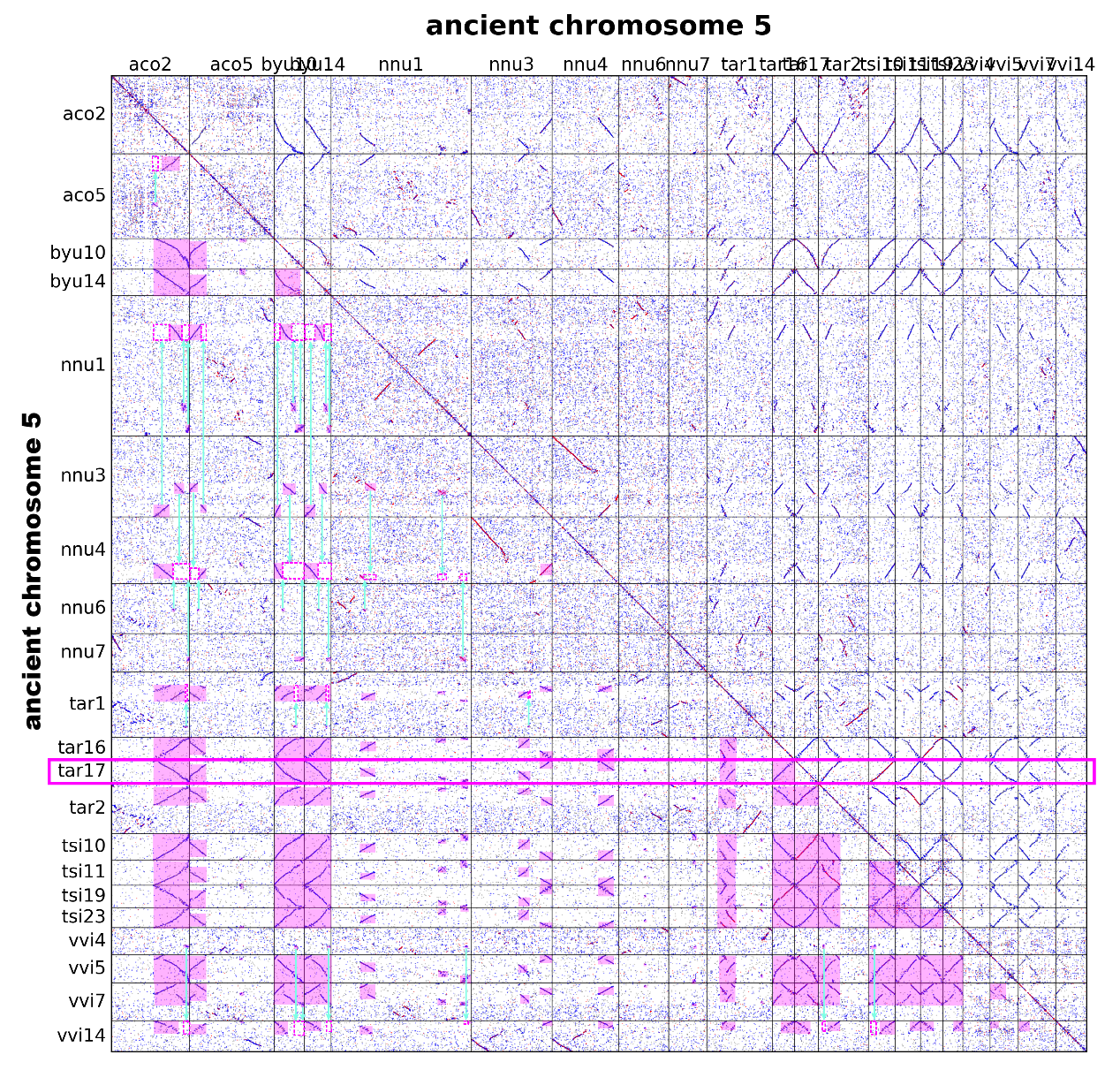
**

## Fig. S22. Demonstration of pieces of AEK 5 in *Aquilegia* (aco), *Buxus austro-yunnanensis* (byu), *Nelumbo* (nnu), *Trochodendron* (tar), *Tetracentron* (tsi) and *Vitis* (vvi). Arrows mean those two pieces belong to one ancient chromosome; chromosome highlighted by box was used as reference chromosome in AEK construction.

**
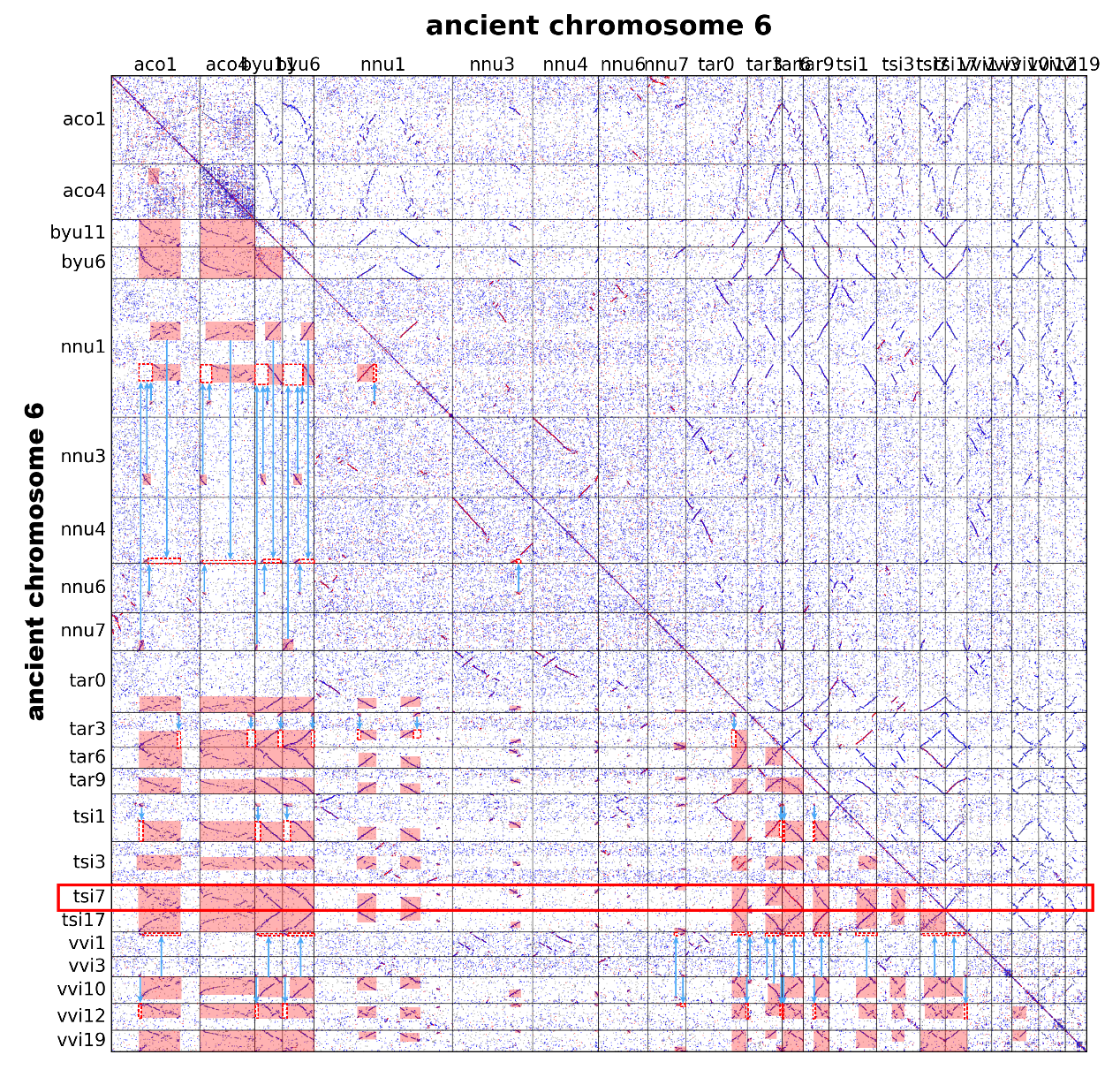
**

## Fig. S23. Demonstration of pieces of AEK 6 in *Aquilegia* (aco), *Buxus austro-yunnanensis* (byu), *Nelumbo* (nnu), *Trochodendron* (tar), *Tetracentron* (tsi) and *Vitis* (vvi). Arrows mean those two pieces belong to one ancient chromosome; chromosome highlighted by box was used as reference chromosome in AEK construction.

**
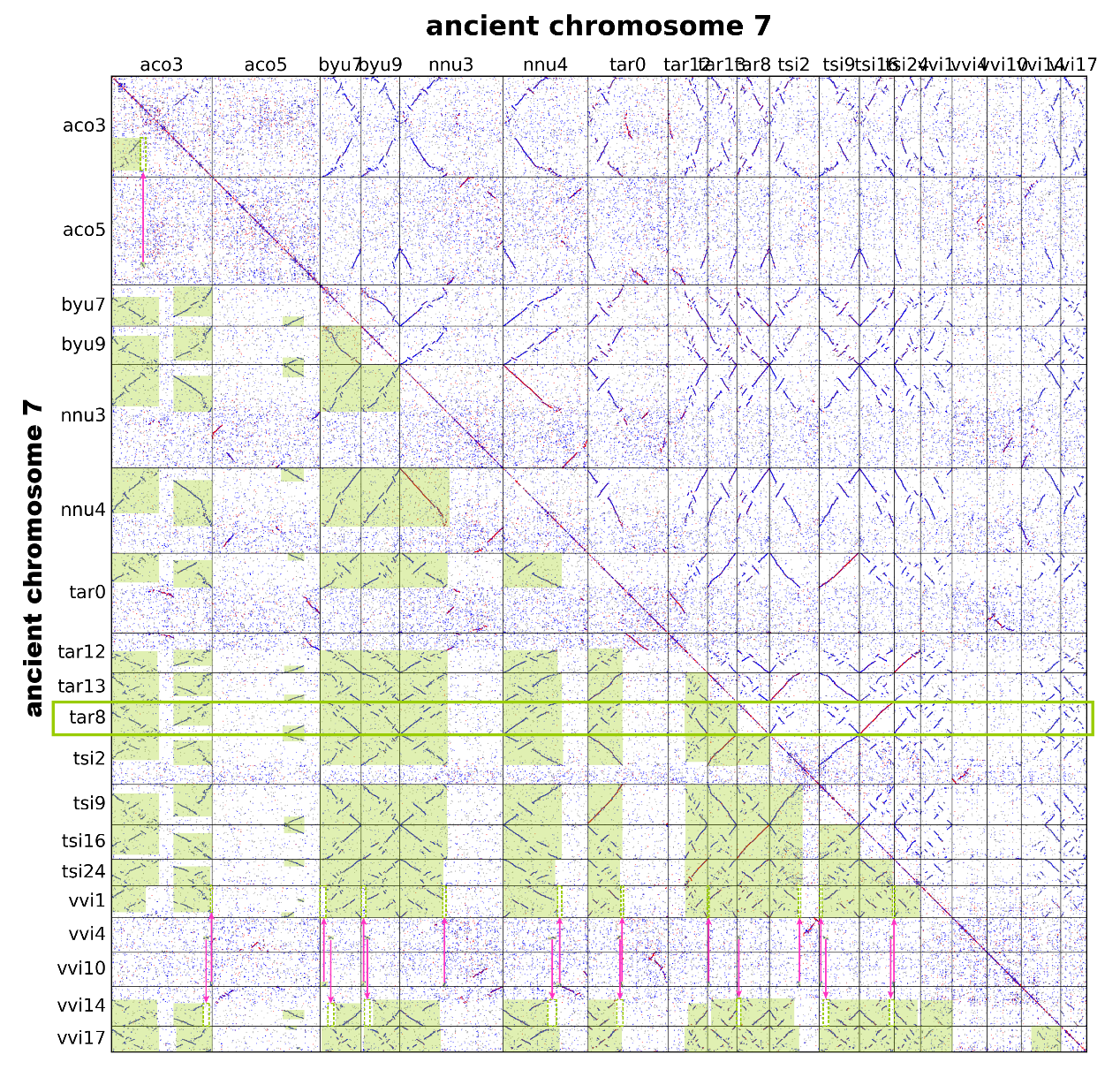
**

## Fig. S24. Demonstration of pieces of AEK 7 in *Aquilegia* (aco), *Buxus austro-yunnanensis* (byu), *Nelumbo* (nnu), *Trochodendron* (tar), *Tetracentron* (tsi) and *Vitis* (vvi). Arrows mean those two pieces belong to one ancient chromosome; chromosome highlighted by box was used as reference chromosome in AEK construction.

**
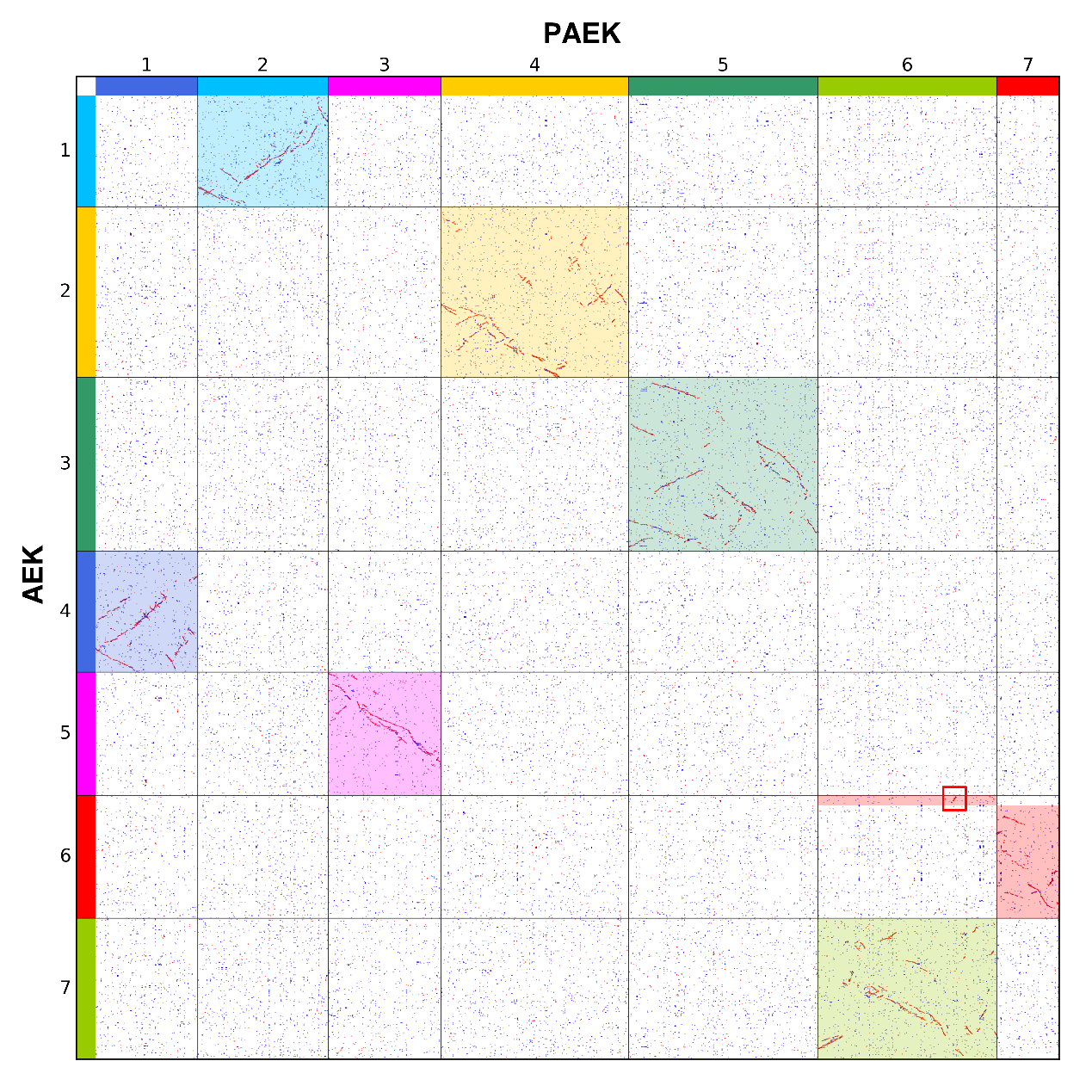
**

## Fig. S25. Comparison between the previous AEK (PAEK) and our constructed AEK. Almost all chromosomes have one-on-one relationship (the collinearity concentrate on corresponding colored square), while only the chromosome 6 of our AEK (shown by red) have a bit collinearity with the chromosome 6 of PAEK (shown by grass green), with the rest part have reasonable collinearity with the chromosome 7 of PAEK (shown by red), which is a signal of interchromosome translocation.

**
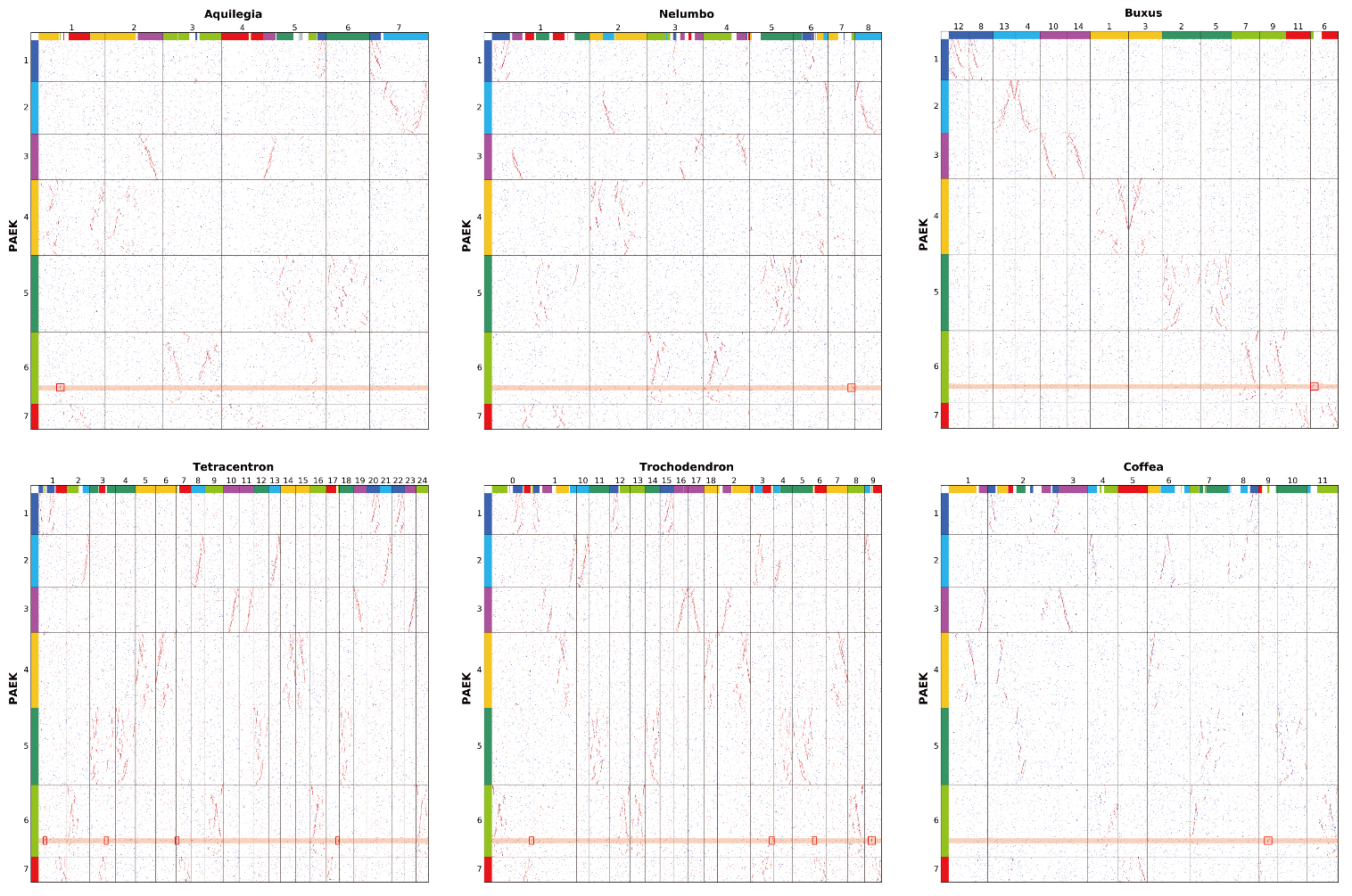
**

## Fig. S26. Karyotype projection of *Aquilegia, Nelumbo*, *Buxus austro-yunnanensis, Tetracentron, Trochodendron* and *Coffea* genome based on PAEK. The corresponding area in PAEK chromosome 6 that shows translocation signal also shows the similar pattern in other early-diverging eudicots (highlighted by red boxes).


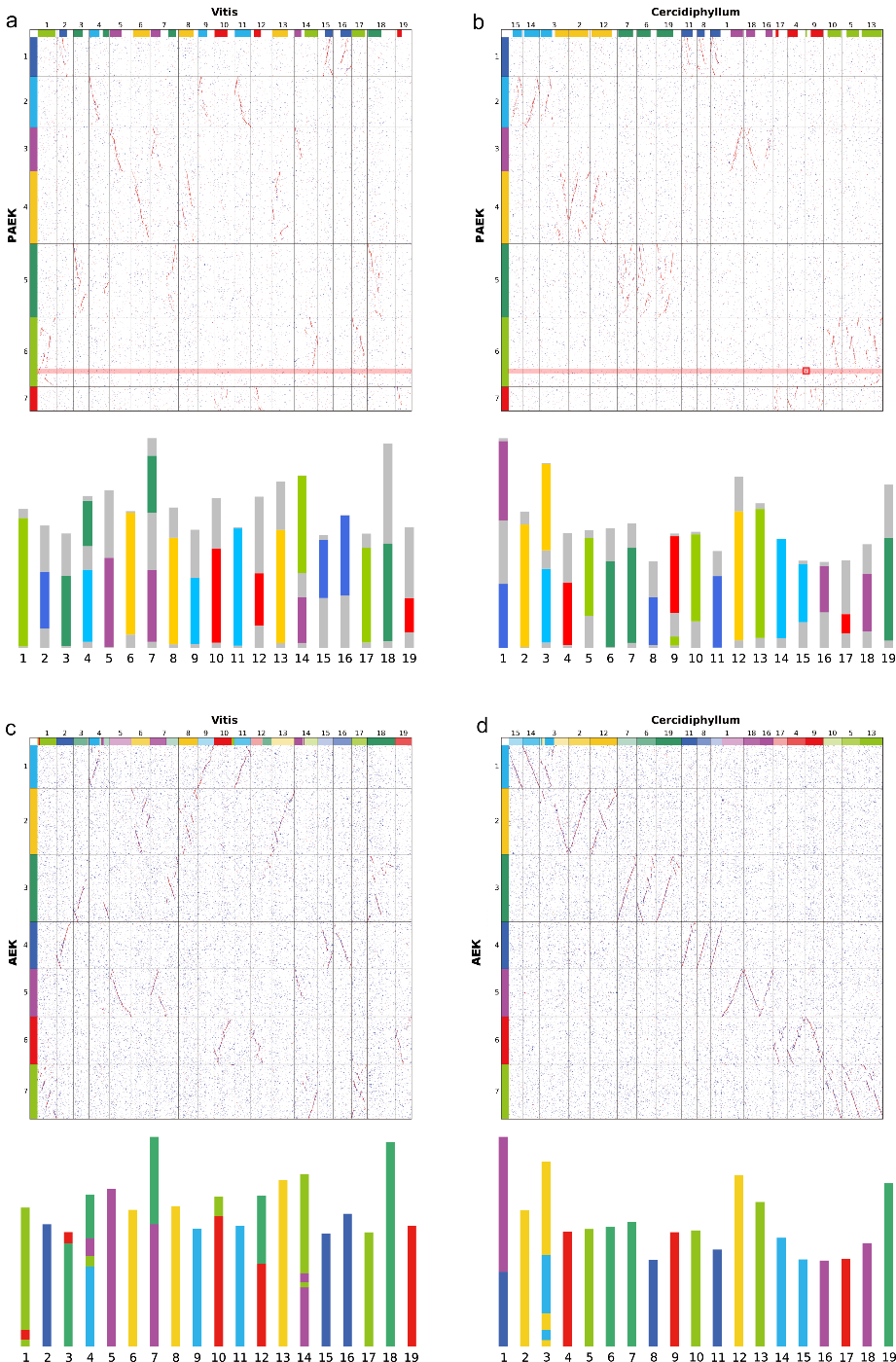


## Fig. S27. Karyotype projection of *Vitis* and *Cercidiphyllum* based on PAEK and AEK. (a) dot plot of *Vitis* to PAEK and its inferred karyotype; (b) dot plot of *Cercidiphyllum* to PAEK and its inferred karyotype; (c) dot plot of *Vitis* to AEK and its inferred karyotype; (d) dot plot of *Cercidiphyllum* to AEK and its inferred karyotype. Dot plot of *Cercidiphyllum* also shows the signal of translocation while only not be detected in the dot plot of *Vitis.* And it is obvious that the collinearity and projections done by AEK in (c) and (d) are more complete.

**
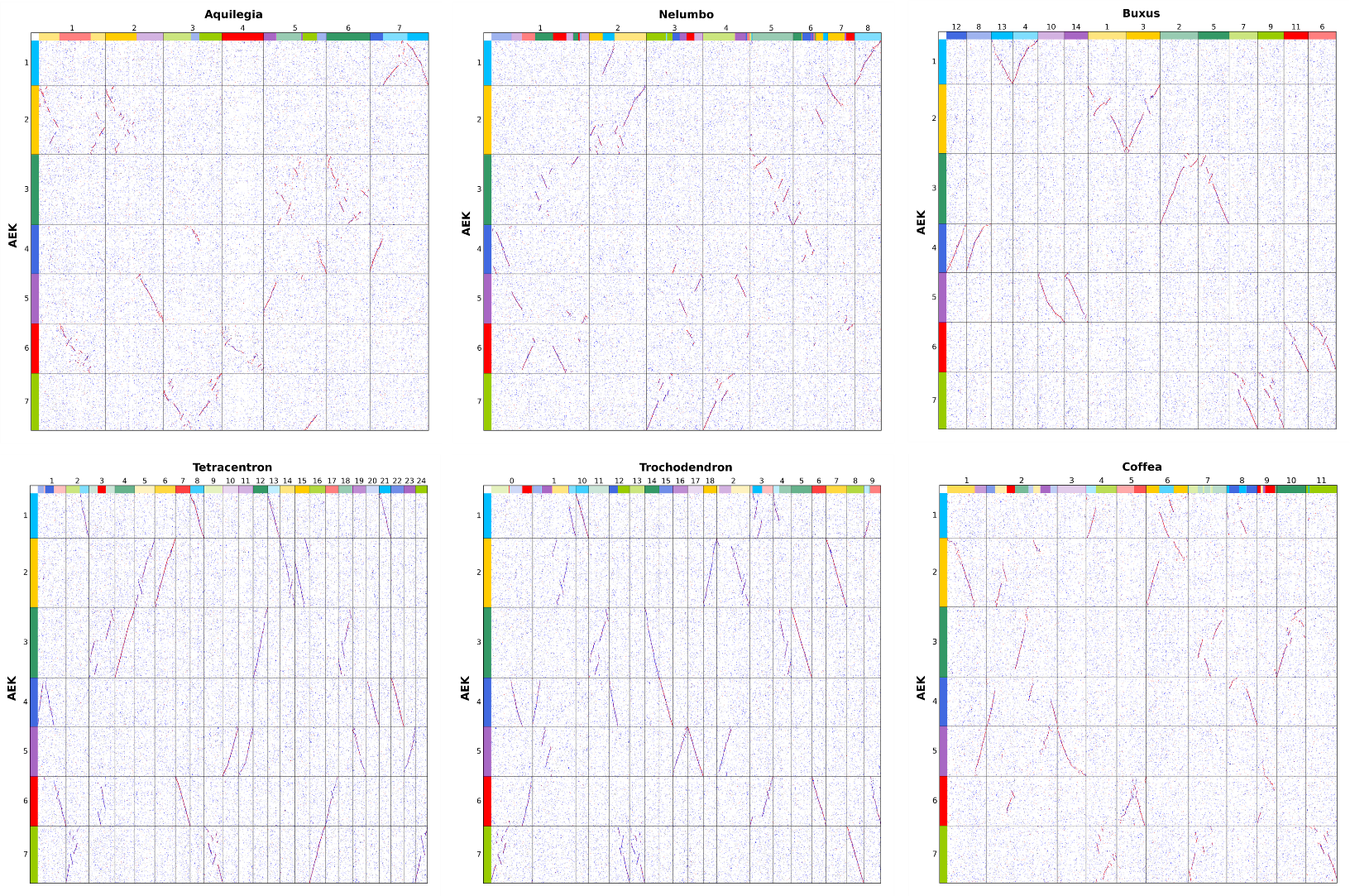
**

## Fig. S28. Karyotype projection of *Aquilegia, Nelumbo, Buxus austro-yunnanensis, Tetracentron, Trochodendron* and *Coffea* genome based on AEK.

##
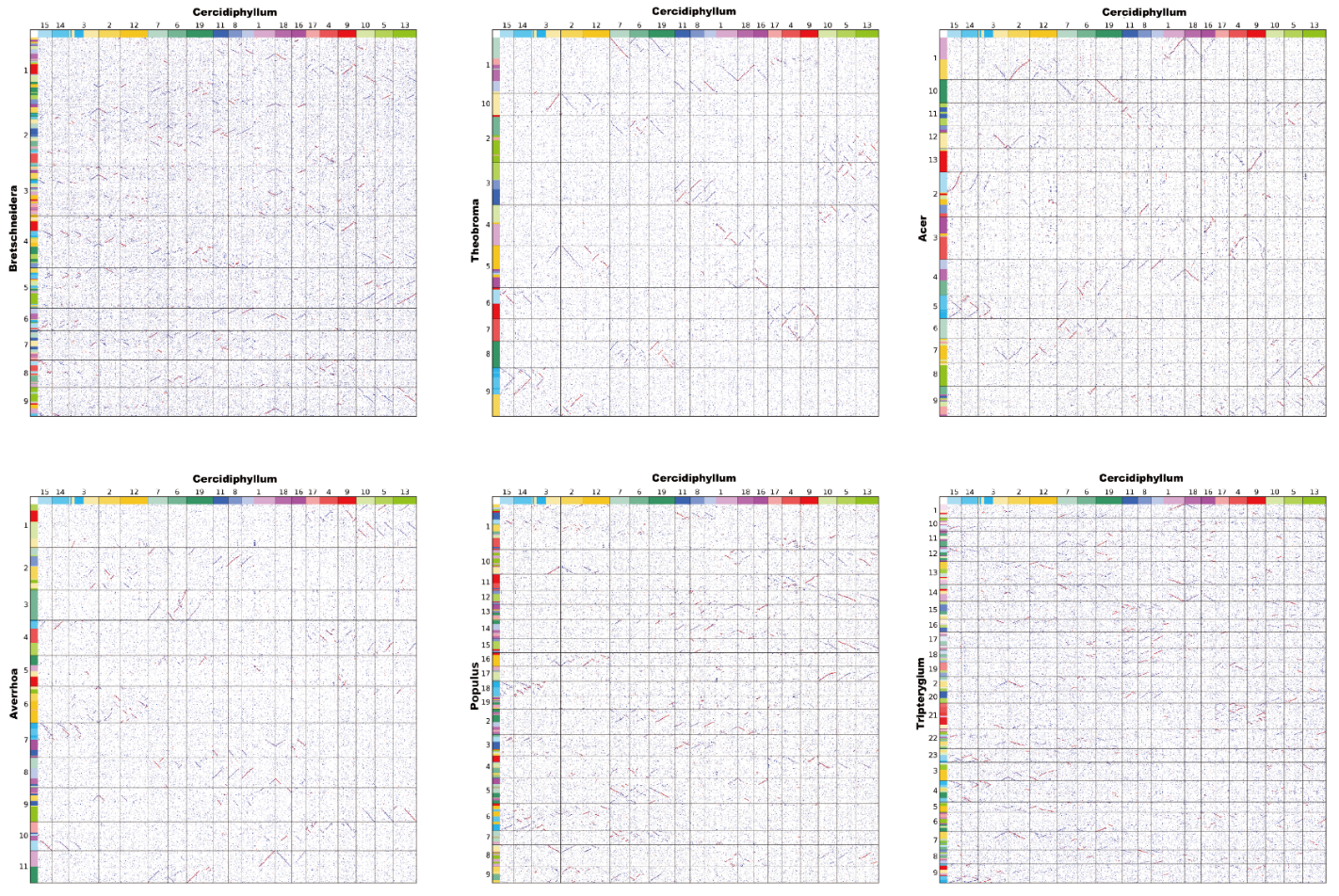
Fig. S29. Collinear gene dot plots and karyotype projection between *Cercidiphyllum* and *Bretschneidera, Theobroma, Acer, Averrhoa, Populus, Tripterygium*.

##
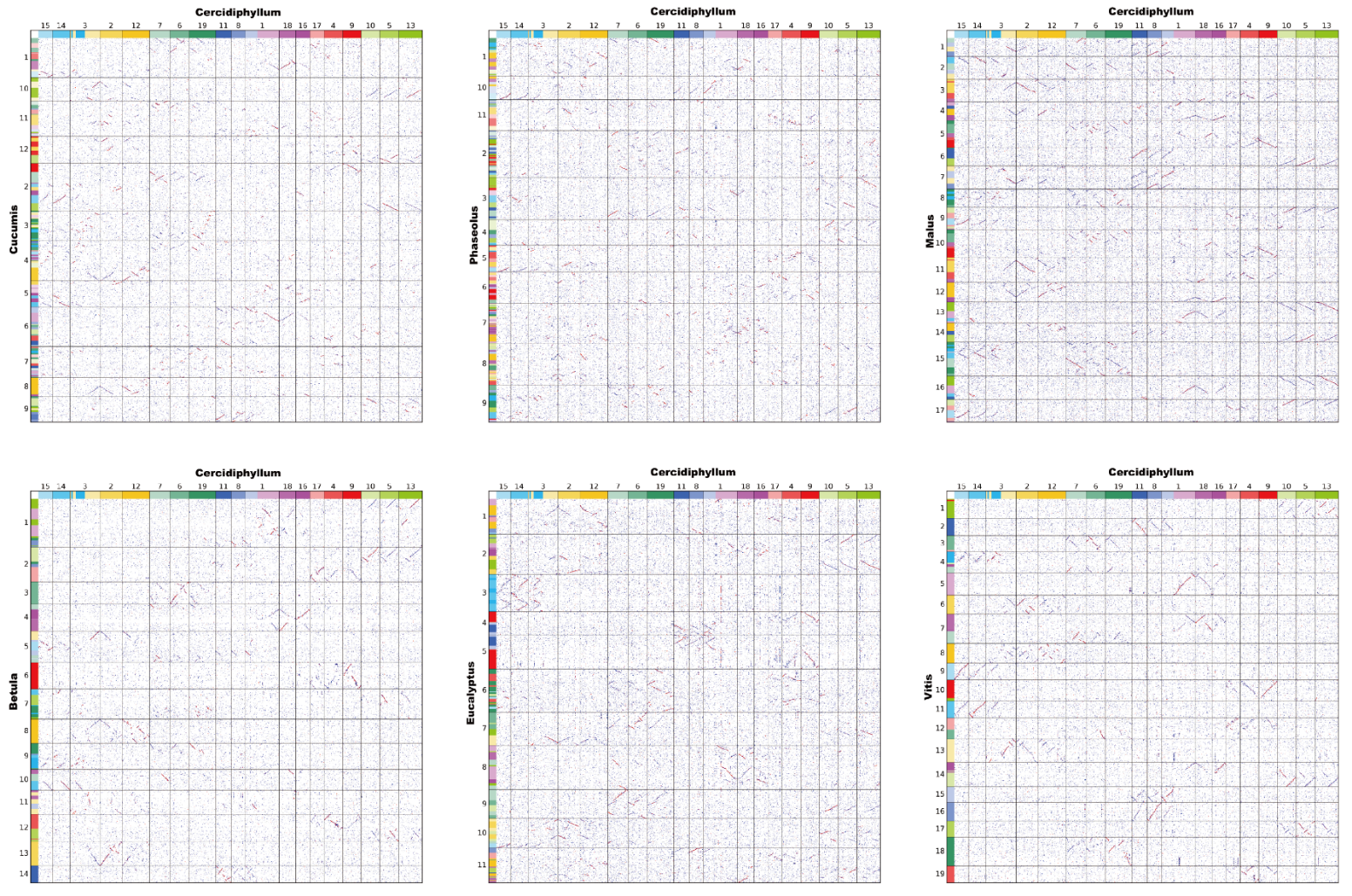
Fig. S30. Collinear gene dot plots and karyotype projection between *Cercidiphyllum* and *Cucumis, Phaseolus, Malus, Betula, Eucalyptus, Vitis*.

##
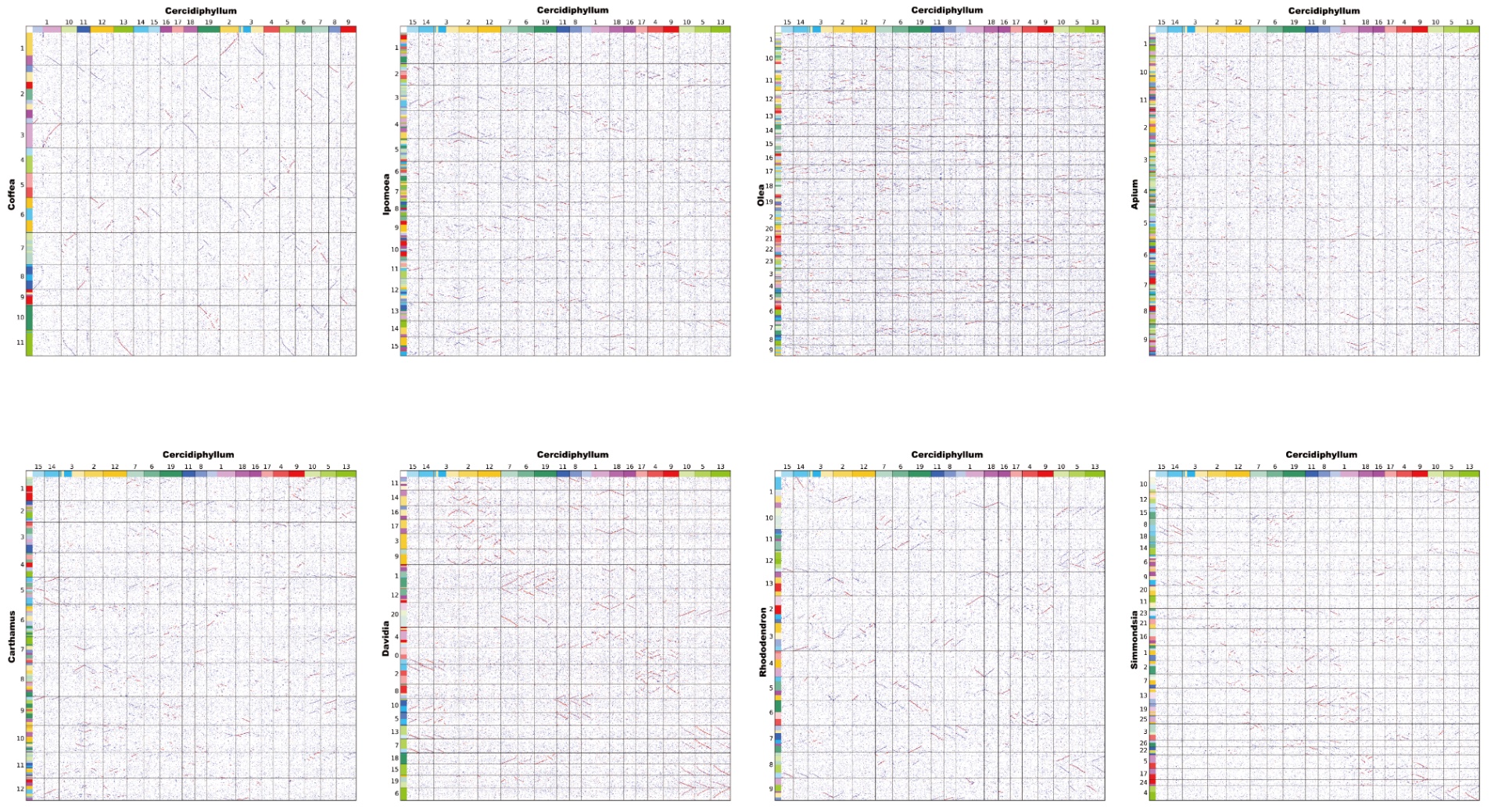
Fig. S31. Collinear gene dot plots and karyotype projection between *Cercidiphyllum* and *Coffea, Ipomoea, Olea, Apium, Carthamus, Davidia, Rhododendron, Simmondsia*.

**
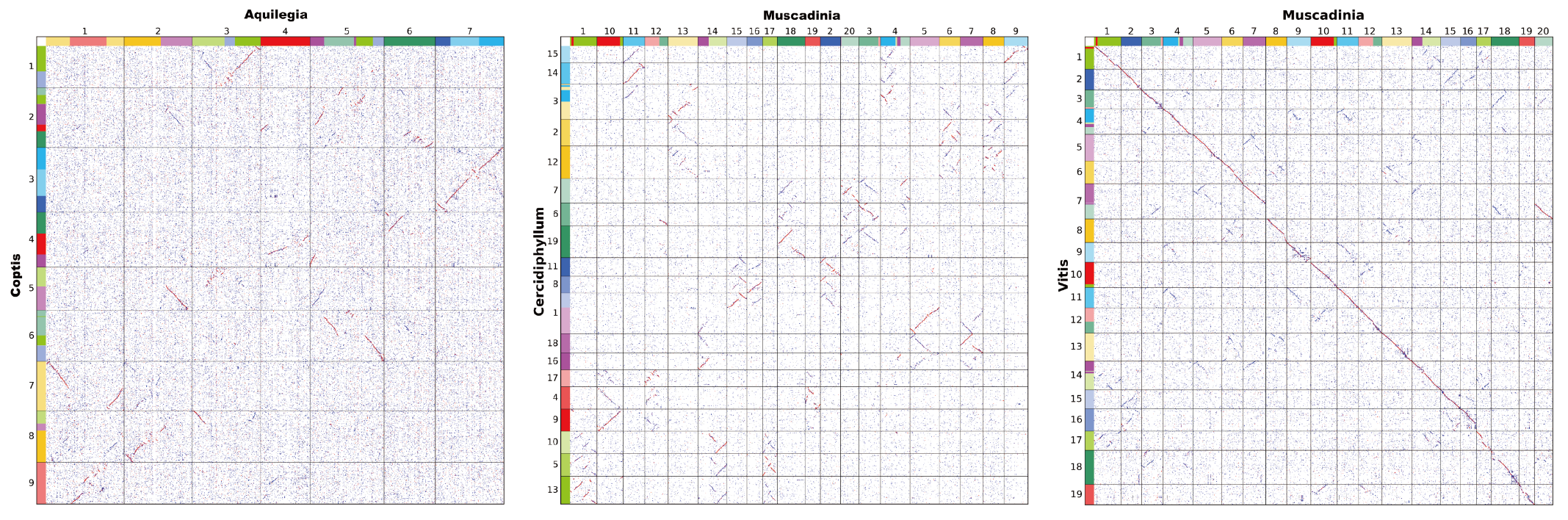
**

## Fig. S32. Collinear gene dot plots and karyotype projection between *Coptis* and *Aquilegia*, *Cercidiphyllum* and *Muscadinia*, *Vitis* and *Muscadinia*.


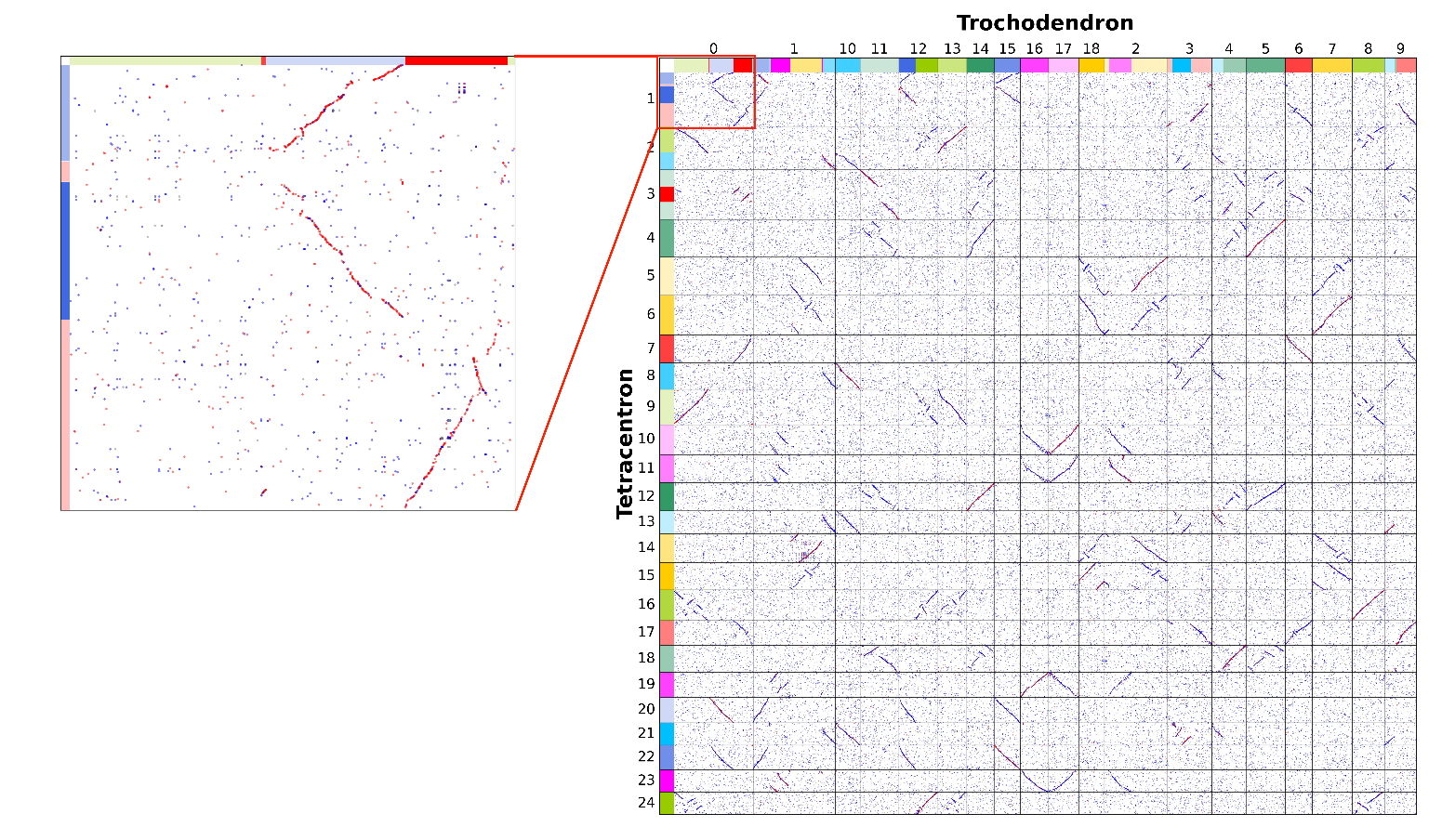


## Fig. S33. Collinear gene dot plots and karyotype projection between *Tetracentron* and *Trochodendron*. The zoomed part indicated that there is no sharing event in *Trochodendron* chr 0 and *Tetracentron* chr 1, because the collinearity near the fusion points is discontinuous, the saturation of color (red and deep blue) also showed that they are the totally different fusions of homologous chromosomes in these two species respectively.


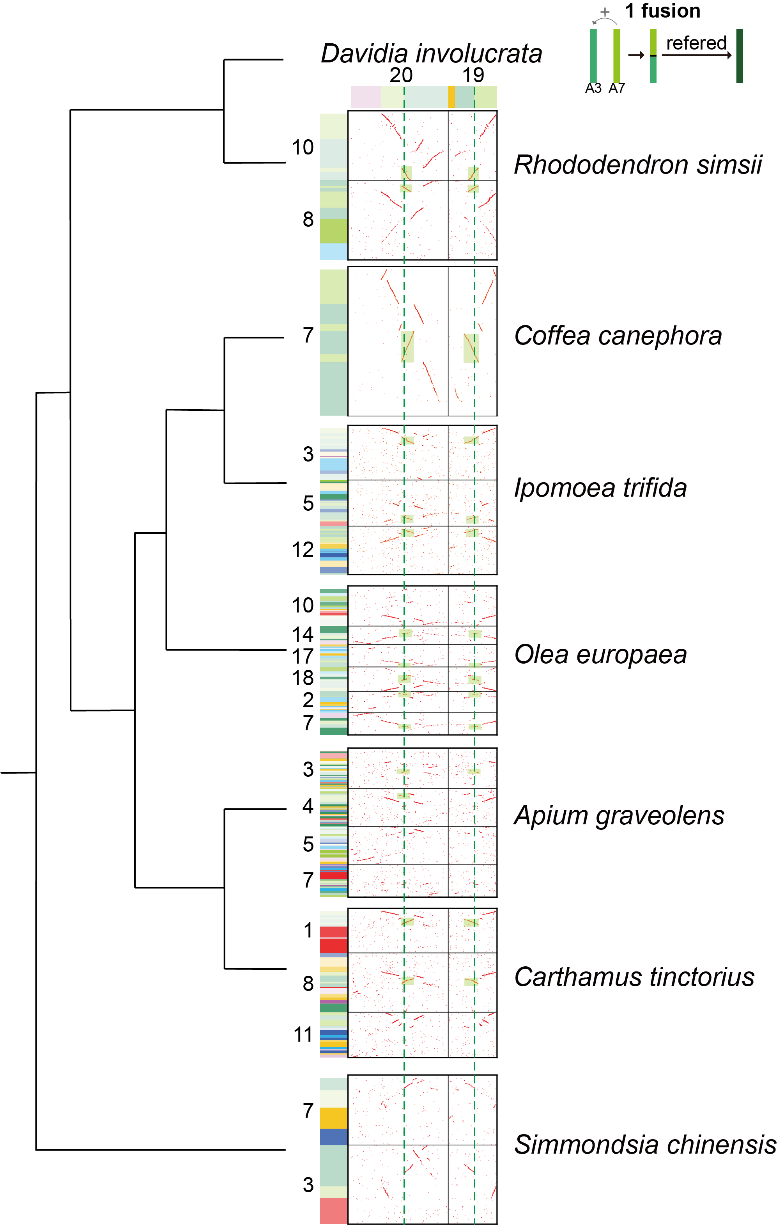


## Fig. S34. Synteny comparison among asterids species with *Davidia* as reference. Two chromosomes colored by deep green and grass green joined end to end to form a new chromosome colored by olive drab. The dash lines underlined the boundary of the chromosome regions that belong to different ancient chromosome (A3 and A7, only noted several boundaries that have collinearity passing through). Although after long-time evolution there may be several changes of karyotype, such as inversion and insertion, so it may not be shown simply as the connection of A3 and A7. However, if the collinearity passed through any dash line continuously, then it means that the variation of ancient chromosome was happened before the speciation, so we regard the related two species sharing this fusion. As all species except *Simmondsia* having this feature, this fusion is shared among asterids with a high probability.


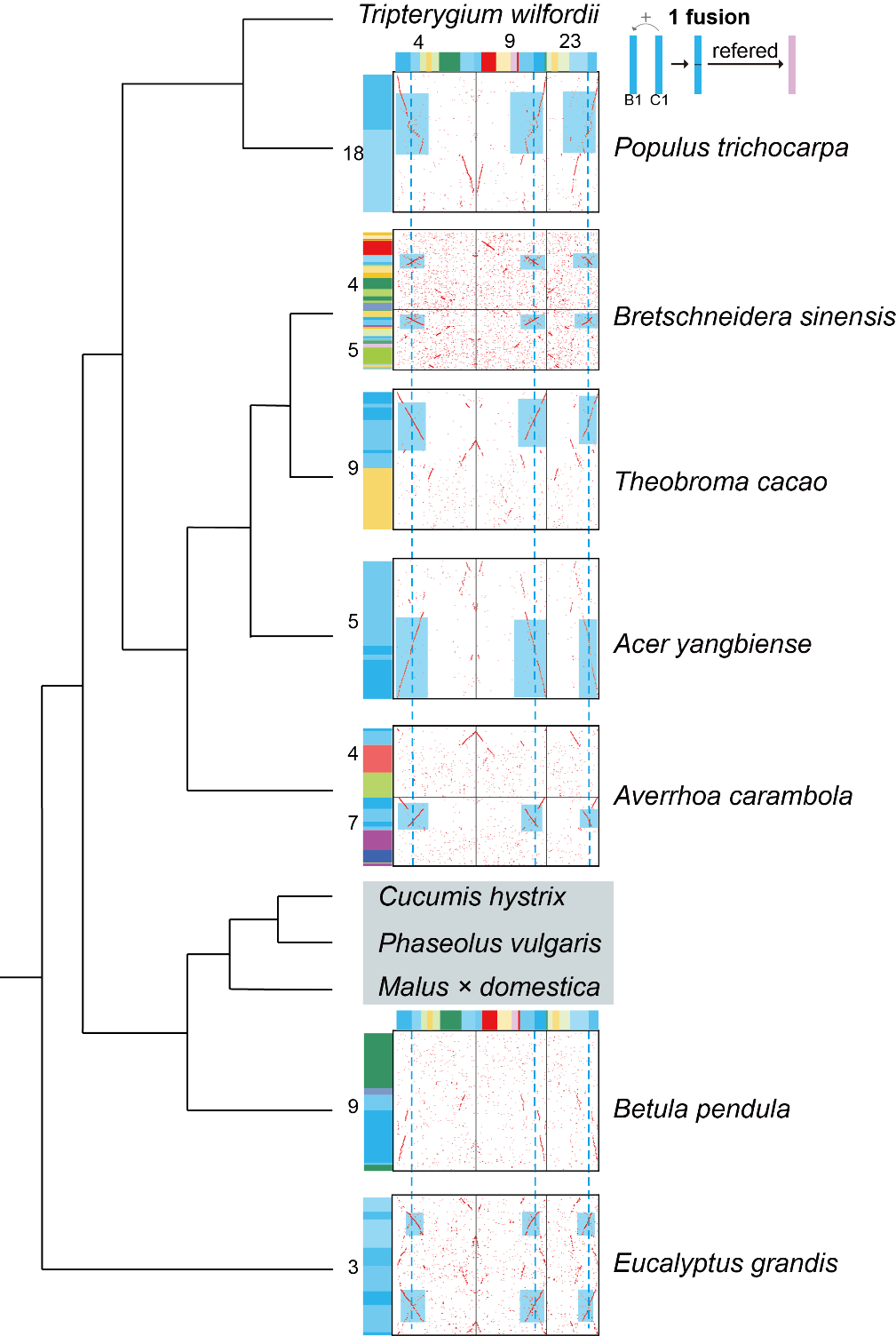


## Fig. S35. Synteny comparison among rosids species with *Tripterygium* as reference. Two homologous chromosomes colored by blue joined end to end to form a new chromosome colored by pink. The saturation of color was adjusted in order to distinguish homologous chromosomes.

## Table S1. The detail sequencing information of *Buxus austro-yunnanesis*.

| **Platform** | **Library type** | **Number of reads** | **Total Bases (Gb)** | **Mean read**  **length (bp)** | **Read N50**  **length (bp)** |
| --- | --- | --- | --- | --- | --- |
| HiSeq2000 | Paired | 212,514,778 | 31.88 | 150 | - |
| ONT PromethION | Single | 3,848,195 | 75.19 | 19,540 | 27,343 |
| Hi-C | Paired | 535,899,574 | 80.38 | 150 | - |

## Table S2. Summary of genome assembly.

|  | **Size (Mb)** | **Number** |
| --- | --- | --- |
| **N90** | 4.57 | 35 |
| **N50** | 18.92 | 12 |
| **Longest** | 46.37 | - |
| **Total Contigs** | 637.31 | 112 |
| **Total number (≥10kb)** | **-** | 112 |
| **Total number (≥100kb)** | **-** | 104 |
| **Contig number (>= 1Mb)** | **-** | 53 |
| **GC content (%)** | **-** | 38.16 |

N50 refer to the size above which 50% of the total length of the sequence assembly can be found.

## Table S3. Contig N50 values of the early-diverging eudicots.

| **Species** | **N50 (mb)** |
| --- | --- |
| *A. coerulea* | 3.1 |
| *N.nucifera* | 3.4 |
| *T.sinense* | 21.2 |
| *T.aralioides* | 0.691 |
| *B.sinica* | 0.164 |
| *B.austro-yunnanesis* | 18.92 |

## Table S4. Summary of chromosome level assembly.

| **Chromosome** | **Length** | **# of contigs** | **GC content** |
| --- | --- | --- | --- |
| **Chr01** | 58,254,268 | 4 | 37.73% |
| **Chr02** | 55,038,786 | 6 | 37.90% |
| **Chr03** | 53,411,867 | 5 | 38.07% |
| **Chr04** | 47,371,842 | 4 | 38.15% |
| **Chr05** | 46,365,631 | 1 | 38.36% |
| **Chr06** | 44,119,909 | 5 | 37.93% |
| **Chr07** | 43,877,054 | 9 | 38.42% |
| **Chr08** | 43,603,720 | 6 | 38.29% |
| **Chr09** | 43,426,168 | 4 | 38.29% |
| **Chr10** | 39,022,411 | 2 | 37.91% |
| **Chr11** | 37,566,837 | 3 | 38.23% |
| **Chr12** | 36,494,383 | 5 | 38.50% |
| **Chr13** | 35,466,724 | 6 | 37.85% |
| **Chr14** | 35,162,719 | 12 | 38.19% |
| **Total** | 619,182,319 | 72 | 38.12% |

## Table S5. Statistic of repetitive elements in the assembled genome.

| **Type** | **Length(bp)** | **percentage of** **repetitive elements (%)** | **percentage of genome (%)** |
| --- | --- | --- | --- |
| DNA | 37,539,117 | 8.94 | 5.89 |
| CMC-EnSpm | 4,364,264 | 1.04 | 0.69 |
| MuDR | 5,917,237 | 1.41 | 0.93 |
| PIF-Harbinger | 1,968,345 | 0.47 | 0.31 |
| hAT-Ac | 11,357,287 | 2.71 | 1.78 |
| hAT-Tip100 | 1,010,283 | 0.24 | 0.16 |
| other | 13,445,246 | 3.20 | 2.11 |
| LINE | 29,458,663 | 7.02 | 4.62 |
| L1 | 23,160,099 | 5.52 | 3.63 |
| L2 | 929,696 | 0.22 | 0.15 |
| other | 5,373,403 | 1.28 | 0.84 |
| LTR | 216,708,100 | 51.63 | 34.00 |
| Copia | 38,182,467 | 9.10 | 5.99 |
| Gypsy | 173,887,675 | 41.43 | 27.28 |
| other | 7,524,022 | 1.79 | 1.18 |
| SINE | 1,818,307 | 0.43 | 0.29 |
| Low_complexity | 1,135,403 | 0.27 | 0.18 |
| Satellite | 913,681 | 0.22 | 0.14 |
| Simple_repeat | 4,802,678 | 1.14 | 0.75 |
| Small_RNA | 363,126 | 0.09 | 0.06 |
| Unclassified_ARTEFACT | 59 | 0.00 | 0.00 |
| Unclassified_Other/Composite | 131 | 0.00 | 0.00 |
| Unclassified_RC | 46,355 | 0.01 | 0.01 |
| Unclassified_RC/Helitron | 5,479,227 | 1.31 | 0.86 |
| Unclassified_Retroposon | 1,034 | 0.00 | 0.00 |
| Unclassified_Retroposon/L1 | 45 | 0.00 | 0.00 |
| Unclassified_Unknown | 144,814,914 | 34.50 | 22.72 |
| Total | 419,758,314 | 100 | 65.86 |

## Table S6. Comparison of gene space of *Buxus austro-yunnanensis* with other genomes.

| **Species** | **Total Genes** | **Average Gene**  **Length (bp)** | **Average CDS Length (bp)** | **Average Exons per Gene** | **Average Exon**  **Length (bp)** | **Average Intron Length (bp)** |
| --- | --- | --- | --- | --- | --- | --- |
| *Aquilegia coerulea* | 43,550 | 4,356.54 | 1,242.35 | 5.53 | 224.65 | 484.65 |
| *Buxus austro-yunnanensis* | 25,542 | 6,027.69 | 1,188.06 | 5.19 | 229.10 | 1,156.22 |
| *Nelumbo nucifera* | 23,989 | 11,923.14 | 1,345.59 | 5.53 | 243.54 | 2,056.28 |
| *Trochodendron aralioides* | 35,328 | 11,326.35 | 1,183.03 | 5.09 | 232.46 | 2,308.46 |
| *Tetracentron sinense* | 37,541 | 7958.03 | 1135.39 | 5.10 | 222.49 | 1662.78 |

|  | *A. coerulea* | | *N. nucifera* | | *B. austro-yunnanensis* | | *B. sinica* | | *T. aralioides* | | *T. sinense* | |
| --- | --- | --- | --- | --- | --- | --- | --- | --- | --- | --- | --- | --- |
| **Description** | **Number of genes** | **% Percentage** | **Number of genes** | **% Percentage** | **Number of genes** | **% Percentage** | **Number of genes** | **% Percentage** | **Number of genes** | **% Percentage** | **Number of genes** | **% Percentage** |
| Complete BUSCOs | 2071 | 97.7 | 2084 | 98.3 | 1946 | 91.7 | 1698 | 80.1 | 1922 | 90.6 | 1894 | 89.2 |
| Complete and single-copy BUSCOs | 1980 | 93.4 | 1847 | 87.1 | 1856 | 87.5 | 1437 | 67.8 | 1561 | 73.6 | 1613 | 76 |
| Complete and duplicated BUSCOs | 91 | 4.3 | 237 | 11.2 | 90 | 4.2 | 261 | 12.3 | 361 | 17 | 281 | 13.2 |
| Fragmented BUSCOs | 30 | 1.4 | 16 | 0.8 | 93 | 4.4 | 205 | 9.7 | 124 | 5.8 | 124 | 5.8 |
| Missing BUSCOs | 20 | 0.9 | 21 | 0.9 | 82 | 3.9 | 218 | 10.2 | 75 | 3.6 | 103 | 5 |

## Table S7. Assessment of the predicted genes by BUSCO (database: eudicotyledons_odb10).

## Table S8. Functional annotation of the predicted genes.

|  | **Database** | **Number** | **Percentage (%)** |
| --- | --- | --- | --- |
| Total |  | 25495 | 100.00 |
| Annotated | InterProscan | 23173 | 90.89 |
|  | GO | 21002 | 82.38 |
|  | NR | 22324 | 87.56 |
|  | SwissProt | 17416 | 68.31 |
|  | TrEMBL | 21955 | 86.11 |
|  | KEGG | 6323 | 24.80 |
| Unannotated |  | 1763 | 6.92 |

## Table S9. Summary of the 28 species genome that used in this study.

| **Species** | **Order** | **DOI** | **Source** |
| --- | --- | --- | --- |
| *Averrhoa carambola* | Oxalidales | 10.1038/s41438-020-0307-3 | https://ngdc.cncb.ac.cn/search/?dbId=gwh&q=GWHABKE00000000 |
| *Aquilegia coerulea* | Ranunculales | 10.7554/eLife.36426 | https://data.jgi.doe.gov/refine-download/phytozome?genome_id=322&expanded=Phytozome-322 |
| *Aristolochia fimbriata* | Piperales | 10.1038/s41477-021-00990-2 | https://ngdc.cncb.ac.cn/search/?dbId=gwh&q=Aristolochia%20fimbriata&page=1 |
| *Apium graveolens* | Apiales | 10.1111/pbi.13499 | http://celerydb.bio2db.com/ |
| *Acer yangbiense* | Sapindales | 10.1093/gigascience/giz085 | http://gigadb.org/dataset/100610 |
| *Betula pendula* | Fagales | 10.1038/ng.3862 | https://genomevolution.org/CoGe/GenomeInfo.pl?gid=35080 |
| *Bretschneidera sinensis* | Brassicales | unpublish | unpublish |
| *Buxus austro-yunnanensis* | Buxales | This work | This work |
| *Coffea canephora* | Gentianales | ensembl | http://ftp.ebi.ac.uk/ensemblgenomes/pub/release-52/plants/fasta/coffea_canephora/cds/ |
| *Cucumis hystrix* | Cucurbitales | 10.1038/s41438-021-00475-5 | https://figshare.com/articles/dataset/Genome_assembly_of_Cucumis_hystrix/13377671 |
| *Cercidiphyllum japonicum* | Saxifragales | 10.1111/nph.16798 | from corresponding author |
| *Carthamus tinctorius* | Asterales | 10.1111/pbi.13586 | https://safflower.scuec.edu.cn/download.html |
| *Davidia involucrata* | Cornales | 10.1111/1755-0998.13138 | https://ngdc.cncb.ac.cn/search/?dbId=gwh&q=Davidia%20involucrata&page=1 |
| *Eucalyptus grandis* | Myrtales | NCBI | https://ftp.ncbi.nlm.nih.gov/genomes/all/GCF/016/545/825/GCF_016545825.1_ASM1654582v1/ |
| *Ipomoea trifida* | Solanales | 10.1038/s41467-018-06983-8 | http://sweetpotato.uga.edu/gt4sp_download.shtml |
| *Malus x domestica* | Rosales | 10.1038/s41467-019-09518-x | https://github.com/moold/Genome-data-of-Hanfu-apple |
| *Nelumbo nucifera* | Proteales | 10.1186/gb-2013-14-5-r41 | https://ftp.ncbi.nlm.nih.gov/genomes/all/GCF/000/365/185/GCF_000365185.1_Chinese_Lotus_1.1/ |
| *Olea europaea* | Lamiales | 10.1038/s41438-021-00498-y | https://ngdc.cncb.ac.cn/search/?dbId=gwh&q=Olea%20europaea&page=1 |
| *Populus trichocarpa* | Malpighiales | JGI | https://data.jgi.doe.gov/refine-download/phytozome?genome_id=533&expanded=Phytozome-533 |
| *Phaseolus vulgaris* | Fabales | JGI | https://data.jgi.doe.gov/refine-download/phytozome?genome_id=442&expanded=Phytozome-442 |
| *Rhododendron simsii* | Ericales | 10.1038/s41467-020-18771-4 | https://bioinformatics.psb.ugent.be/plaza/versions/plaza_v5_dicots/download/download |
| *Simmondsia chinensis* | Caryophyllales | 10.1126/sciadv.aay3240 | https://ngdc.cncb.ac.cn/search/?dbId=gwh&q=GWHAASQ00000000 |
| *Trochodendron aralioides* | Trochodendrales | 10.1093/gigascience/giz136 | https://ftp.cngb.org/pub/gigadb/pub/10.5524/100001_101000/100657/Analyses/Structure/ |
| *Theobroma cacao* | Malvales | 10.1186/s12864-017-4120-9 | http://ftp.ebi.ac.uk/ensemblgenomes/pub/release-52/plants/gff3/theobroma_cacao_criollo/ |
| *Tetracentron sinense* | Trochodendrales | 10.1111/1755-0998.13334 | https://figshare.com/articles/dataset/Tetracentron_sinense_genome/12415928 |
| *Tripterygium wilfordii* | Celastrales | 10.1038/s41467-020-14776-1 | https://ftp.ncbi.nlm.nih.gov/genomes/all/GCF/013/401/445/GCF_013401445.1_ASM1340144v1/ |
| *Vitis vinifera* | Vitales | Ensembl | http://ftp.ebi.ac.uk/ensemblgenomes/pub/release-52/plants/fasta/vitis_vinifera |
| *Liriodendron chinense* | Magnoliales | 10.1038/s41477-018-0323-6 | https://ftp.ncbi.nlm.nih.gov/genomes/all/GCA/003/013/855/GCA_003013855.2_NJFU_Lchi_2.0/ |

## Table S10. Average total introgression proportion per species pair in QuIBL analysis.

| **Pair** | **Mean total hybridization proportion** |
| --- | --- |
| Aco_Byu | 0.171547255 |
| Aco_Cja | 0.198966987 |
| Aco_Din | 0.192854514 |
| Aco_Nnu | 0.183965458 |
| Aco_Sch | 0.180049406 |
| Aco_Tar | 0.197387357 |
| Aco_Vvi | 0.196292394 |
| Byu_Cja | 0.183151833 |
| Byu_Din | 0.185206374 |
| Byu_Sch | 0.182227838 |
| Byu_Vvi | 0.187091666 |
| Cja_Din | 0.12973407 |
| Cja_Nnu | 0.079191773 |
| Cja_Sch | 0.113414194 |
| Cja_Tar | 0.218624962 |
| Din_Nnu | 0.088397517 |
| Din_Tar | 0.219747939 |
| Nnu_Sch | 0.078608889 |
| Nnu_Tar | 0.109710291 |
| Nnu_Vvi | 0.08705101 |
| Sch_Tar | 0.207882201 |
| Sch_Vvi | 0.157198917 |
| Tar_Vvi | 0.218051923 |
| All | 0.163754555 |

## Table S11. The QuIBL analysis result. Only the significant results were used for the statistics in Table S10. Three letter abbreviation including: Afi for *Aristolochia*, Aco for *Aquilegia*, Nnu for *Nelumbo*, Byu for *Buxus*, Tar for *Trochodendron*, Sch for *Simmondsia*, Din for *Davidia*, Cja for *Cercidiphyllum* and Vvi for *Vitis*.

| triplet | outgroup | C1 | C2 | mixprop1 | mixprop2 | lambda2Dist | lambda1Dist | BIC2Dist | BIC1Dist | count | BICdiff | Intro_Prop | true_outgroup | Significant | IsMostCommon | Pair |
| --- | --- | --- | --- | --- | --- | --- | --- | --- | --- | --- | --- | --- | --- | --- | --- | --- |
| Aco_Nnu_Byu | Aco | 0 | 0.922004 | 0.191117 | 0.808883 | 0.017045 | 0.025017 | -3261.957 | -3095.83 | 577 | -166.1275 | 0.386362 | Aco | TRUE | TRUE | Byu_Nnu |
| Aco_Nnu_Byu | Nnu | 0 | 0.796667 | 0.335804 | 0.664196 | 0.017852 | 0.023742 | -1744.371 | -1704.324 | 312 | -40.04675 | 0.171547 | Aco | TRUE | TRUE | Aco_Byu |
| Aco_Nnu_Byu | Byu | 0 | 1.2099 | 0.231229 | 0.768771 | 0.015146 | 0.02408 | -1805.86 | -1733.653 | 319 | -72.20764 | 0.203012 | Aco | TRUE | TRUE | Aco_Nnu |
| Aco_Nnu_Tar | Aco | 0 | 0.930458 | 0.204589 | 0.795411 | 0.016728 | 0.024451 | -3265.532 | -3105.969 | 574 | -159.5625 | 0.377952 | Aco | TRUE | TRUE | Nnu_Tar |
| Aco_Nnu_Tar | Nnu | 0 | 0.753749 | 0.261784 | 0.738216 | 0.016464 | 0.022093 | -1873.767 | -1811.103 | 323 | -62.66489 | 0.197387 | Aco | TRUE | TRUE | Aco_Tar |
| Aco_Nnu_Tar | Tar | 0 | 1.33686 | 0.254629 | 0.745371 | 0.013499 | 0.022162 | -1807.085 | -1741.69 | 311 | -65.39458 | 0.191896 | Aco | TRUE | TRUE | Aco_Nnu |
| Aco_Nnu_Din | Aco | 0 | 1.194321 | 0.160422 | 0.839578 | 0.014232 | 0.023251 | -2974.139 | -2804.888 | 509 | -169.2511 | 0.353763 | Aco | TRUE | TRUE | Din_Nnu |
| Aco_Nnu_Din | Nnu | 0 | 0.610686 | 0.300396 | 0.699604 | 0.020998 | 0.026428 | -1801.101 | -1747.98 | 333 | -53.12082 | 0.192855 | Aco | TRUE | TRUE | Aco_Din |
| Aco_Nnu_Din | Din | 0 | 0.455627 | 0.415333 | 0.584667 | 0.029839 | 0.034506 | -1760.034 | -1726.456 | 366 | -33.57754 | 0.177142 | Aco | TRUE | TRUE | Aco_Nnu |
| Aco_Nnu_Vvi | Aco | 0 | 1.183217 | 0.159801 | 0.840199 | 0.014324 | 0.023347 | -2970.808 | -2800.696 | 509 | -170.1112 | 0.354024 | Aco | TRUE | TRUE | Nnu_Vvi |
| Aco_Nnu_Vvi | Nnu | 0 | 0.621253 | 0.292175 | 0.707825 | 0.020641 | 0.026137 | -1821.825 | -1765.933 | 335 | -55.89247 | 0.196292 | Aco | TRUE | TRUE | Aco_Vvi |
| Aco_Nnu_Vvi | Vvi | 0 | 0.510788 | 0.39929 | 0.60071 | 0.027643 | 0.032722 | -1794.816 | -1755.641 | 364 | -39.17482 | 0.181009 | Aco | TRUE | TRUE | Aco_Nnu |
| Aco_Nnu_Cja | Aco | 0 | 1.18739 | 0.161908 | 0.838092 | 0.014315 | 0.023318 | -2982.04 | -2812.975 | 511 | -169.0651 | 0.354524 | Aco | TRUE | TRUE | Cja_Nnu |
| Aco_Nnu_Cja | Nnu | 0 | 0.632047 | 0.282531 | 0.717469 | 0.020376 | 0.02599 | -1828.222 | -1769.706 | 335 | -58.51548 | 0.198967 | Aco | TRUE | TRUE | Aco_Cja |
| Aco_Nnu_Cja | Cja | 0 | 0.554939 | 0.389501 | 0.610499 | 0.026687 | 0.032065 | -1800.804 | -1760.658 | 362 | -40.14606 | 0.182948 | Aco | TRUE | TRUE | Aco_Nnu |
| Aco_Nnu_Sch | Aco | 0 | 1.194426 | 0.162047 | 0.837953 | 0.014247 | 0.023267 | -2966.555 | -2798.645 | 508 | -167.91 | 0.352384 | Aco | TRUE | TRUE | Nnu_Sch |
| Aco_Nnu_Sch | Nnu | 0 | 0.541346 | 0.342901 | 0.657099 | 0.023021 | 0.027925 | -1743.2 | -1700.991 | 331 | -42.20828 | 0.180049 | Aco | TRUE | TRUE | Aco_Sch |
| Aco_Nnu_Sch | Sch | 0 | 0.366919 | 0.450715 | 0.549285 | 0.033169 | 0.037054 | -1714.863 | -1688.086 | 369 | -26.77703 | 0.167787 | Aco | TRUE | TRUE | Aco_Nnu |
| Aco_Byu_Tar | Aco | 0 | 1.698978 | 0.190635 | 0.809365 | 0.016652 | 0.03132 | -4702.846 | -4422.562 | 899 | -280.2844 | 0.602334 | Aco | TRUE | TRUE | Byu_Tar |
| Aco_Byu_Tar | Byu | 0 | 1.53897 | 0.374815 | 0.625185 | 0.012785 | 0.020701 | -970.0256 | -961.7375 | 168 | -8.288133 | 0.086946 | Aco | FALSE | TRUE | Aco_Tar |
| Aco_Byu_Tar | Tar | 0 | 1.876764 | 0.574752 | 0.425248 | 0.013255 | 0.020995 | -788.8222 | -802.5525 | 141 | 13.73025 | 0.049636 | Aco | FALSE | TRUE | Aco_Byu |
| Aco_Byu_Din | Aco | 0 | 1.605014 | 0.152732 | 0.847268 | 0.01628 | 0.030517 | -4297.938 | -3991.377 | 803 | -306.5608 | 0.563209 | Aco | TRUE | TRUE | Byu_Din |
| Aco_Byu_Din | Byu | 0 | 1.011008 | 0.4849 | 0.5151 | 0.020618 | 0.027581 | -948.2577 | -948.1366 | 184 | -0.121088 | 0.078459 | Aco | FALSE | TRUE | Aco_Din |
| Aco_Byu_Din | Din | 0 | 0.792733 | 0.658173 | 0.341827 | 0.033214 | 0.039048 | -974.4434 | -985.993 | 221 | 11.5496 | 0.062536 | Aco | FALSE | TRUE | Aco_Byu |
| Aco_Byu_Vvi | Aco | 0 | 1.606953 | 0.151681 | 0.848319 | 0.016254 | 0.03051 | -4304.251 | -3996.726 | 804 | -307.5247 | 0.56461 | Aco | TRUE | TRUE | Byu_Vvi |
| Aco_Byu_Vvi | Byu | 0 | 1.06277 | 0.453142 | 0.546858 | 0.019687 | 0.02715 | -962.4358 | -959.1341 | 185 | -3.301775 | 0.083749 | Aco | FALSE | TRUE | Aco_Vvi |
| Aco_Byu_Vvi | Vvi | 0 | 0.91463 | 0.608658 | 0.391342 | 0.029114 | 0.036091 | -1003.129 | -1011.525 | 219 | 8.395736 | 0.070947 | Aco | FALSE | TRUE | Aco_Byu |
| Aco_Byu_Cja | Aco | 0 | 1.656576 | 0.160446 | 0.839554 | 0.016097 | 0.030505 | -4301.452 | -4001.969 | 805 | -299.483 | 0.559471 | Aco | TRUE | TRUE | Byu_Cja |
| Aco_Byu_Cja | Byu | 0 | 1.057158 | 0.464477 | 0.535523 | 0.019718 | 0.02699 | -969.2924 | -966.5516 | 186 | -2.740832 | 0.082456 | Aco | FALSE | TRUE | Aco_Cja |
| Aco_Byu_Cja | Cja | 0 | 0.960559 | 0.582335 | 0.417665 | 0.027698 | 0.035239 | -1005.855 | -1012.61 | 217 | 6.754974 | 0.075028 | Aco | FALSE | TRUE | Aco_Byu |
| Aco_Byu_Sch | Aco | 0 | 1.579097 | 0.153976 | 0.846024 | 0.016547 | 0.030731 | -4272.415 | -3970.259 | 801 | -302.1565 | 0.560981 | Aco | TRUE | TRUE | Byu_Sch |
| Aco_Byu_Sch | Byu | 0 | 0.753043 | 0.520091 | 0.479909 | 0.024731 | 0.030262 | -917.4061 | -918.9895 | 185 | 1.583479 | 0.073496 | Aco | FALSE | TRUE | Aco_Sch |
| Aco_Byu_Sch | Sch | 0 | 0.322395 | 0.621149 | 0.378851 | 0.041057 | 0.043938 | -935.1869 | -938.0914 | 222 | 2.904481 | 0.069623 | Aco | FALSE | TRUE | Aco_Byu |
| Aco_Tar_Din | Aco | 0 | 1.880984 | 0.153888 | 0.846112 | 0.014877 | 0.029948 | -4358.957 | -4046.714 | 808 | -312.2429 | 0.565942 | Aco | TRUE | TRUE | Din_Tar |
| Aco_Tar_Din | Tar | 0 | 1.009336 | 0.48235 | 0.51765 | 0.019033 | 0.025583 | -944.9607 | -943.8583 | 178 | -1.102359 | 0.076276 | Aco | FALSE | TRUE | Aco_Din |
| Aco_Tar_Din | Din | 0 | 4.123797 | 0.82488 | 0.17512 | 0.025185 | 0.038855 | -973.5743 | -992.6676 | 222 | 19.09324 | 0.032183 | Aco | FALSE | TRUE | Aco_Tar |
| Aco_Tar_Vvi | Aco | 0 | 1.843389 | 0.150709 | 0.849291 | 0.015042 | 0.030002 | -4365.101 | -4048.801 | 809 | -316.3004 | 0.568772 | Aco | TRUE | TRUE | Tar_Vvi |
| Aco_Tar_Vvi | Tar | 0 | 1.189454 | 0.464311 | 0.535689 | 0.017525 | 0.024982 | -958.6801 | -957.6916 | 179 | -0.988492 | 0.079378 | Aco | FALSE | TRUE | Aco_Vvi |
| Aco_Tar_Vvi | Vvi | 0 | 3.707762 | 0.833893 | 0.166107 | 0.023508 | 0.035774 | -996.1176 | -1020.04 | 220 | 23.9224 | 0.030251 | Aco | FALSE | TRUE | Aco_Tar |
| Aco_Tar_Cja | Aco | 0 | 1.891823 | 0.158479 | 0.841521 | 0.014919 | 0.030008 | -4361.116 | -4053.481 | 810 | -307.6349 | 0.564265 | Aco | TRUE | TRUE | Cja_Tar |
| Aco_Tar_Cja | Tar | 0 | 1.118848 | 0.46567 | 0.53433 | 0.017708 | 0.024774 | -967.9979 | -966.0729 | 180 | -1.924988 | 0.079619 | Aco | FALSE | TRUE | Aco_Cja |
| Aco_Tar_Cja | Cja | 0 | 2.766425 | 0.789929 | 0.210071 | 0.023149 | 0.034879 | -989.7101 | -1021.772 | 218 | 32.06189 | 0.03791 | Aco | FALSE | TRUE | Aco_Tar |
| Aco_Tar_Sch | Aco | 0 | 1.813639 | 0.160288 | 0.839712 | 0.015411 | 0.030294 | -4310.819 | -4013.183 | 805 | -297.6357 | 0.559576 | Aco | TRUE | TRUE | Sch_Tar |
| Aco_Tar_Sch | Tar | 0 | 0.703028 | 0.518134 | 0.481866 | 0.023346 | 0.028291 | -907.1904 | -908.0395 | 178 | 0.849101 | 0.071003 | Aco | FALSE | TRUE | Aco_Sch |
| Aco_Tar_Sch | Sch | 0 | 0.857181 | 0.842366 | 0.157634 | 0.039354 | 0.043151 | -940.5407 | -958.959 | 225 | 18.41826 | 0.029361 | Aco | FALSE | TRUE | Aco_Tar |
| Aco_Din_Vvi | Aco | 0 | 2.643932 | 0.0055 | 0.9945 | 0.03158 | 0.090138 | -4495.543 | -3272.706 | 1166 | -1222.837 | 0.959923 | Aco | TRUE | TRUE | Din_Vvi |
| Aco_Din_Vvi | Din | 0 | 0.973705 | 0.120571 | 0.879429 | 0.030302 | 0.0466 | -108.2358 | -104.1822 | 26 | -4.053687 | 0.018928 | Aco | FALSE | TRUE | Aco_Vvi |
| Aco_Din_Vvi | Vvi | 0 | 2.564936 | 0.471353 | 0.528647 | 0.016359 | 0.033621 | -68.88107 | -73.7909 | 16 | 4.909829 | 0.007002 | Aco | FALSE | TRUE | Aco_Din |
| Aco_Din_Cja | Aco | 0 | 2.738532 | 0.007891 | 0.992109 | 0.030476 | 0.089308 | -4539.393 | -3305.585 | 1170 | -1233.808 | 0.9609 | Aco | TRUE | TRUE | Cja_Din |
| Aco_Din_Cja | Din | 0 | 0.780699 | 0.155686 | 0.844314 | 0.029598 | 0.041884 | -121.7279 | -118.3472 | 28 | -3.380669 | 0.01957 | Aco | FALSE | TRUE | Aco_Cja |
| Aco_Din_Cja | Cja | 0 | 4.208118 | 0.49584 | 0.50416 | 0.009282 | 0.02449 | -49.31374 | -51.88755 | 10 | 2.573813 | 0.004174 | Aco | FALSE | TRUE | Aco_Din |
| Aco_Din_Sch | Aco | 0 | 2.556527 | 0.011173 | 0.988827 | 0.034866 | 0.096551 | -4234.677 | -3096.37 | 1160 | -1138.306 | 0.949535 | Aco | TRUE | TRUE | Din_Sch |
| Aco_Din_Sch | Din | 0 | 0.288034 | 0.612629 | 0.387371 | 0.055622 | 0.059223 | -64.67805 | -70.0622 | 20 | 5.384149 | 0.006413 | Aco | FALSE | TRUE | Aco_Sch |
| Aco_Din_Sch | Sch | 0 | 3.441696 | 0.588874 | 0.411126 | 0.029364 | 0.063766 | -89.39374 | -94.80992 | 28 | 5.416179 | 0.009529 | Aco | FALSE | TRUE | Aco_Din |
| Aco_Vvi_Cja | Aco | 0 | 2.634863 | 0.008781 | 0.991219 | 0.032684 | 0.092817 | -4421.702 | -3229.171 | 1175 | -1192.531 | 0.964141 | Aco | TRUE | TRUE | Cja_Vvi |
| Aco_Vvi_Cja | Vvi | 0 | 3.648526 | 0.444076 | 0.555924 | 0.013177 | 0.033419 | -80.87716 | -83.45983 | 18 | 2.582666 | 0.008284 | Aco | FALSE | TRUE | Aco_Cja |
| Aco_Vvi_Cja | Cja | 0 | 2.05417 | 0.244247 | 0.755753 | 0.011363 | 0.023157 | -79.39996 | -80.25611 | 15 | 0.856146 | 0.009384 | Aco | FALSE | TRUE | Aco_Vvi |
| Aco_Vvi_Sch | Aco | 0 | 2.452676 | 0.01044 | 0.98956 | 0.034217 | 0.091456 | -4299.795 | -3208.23 | 1155 | -1091.565 | 0.946144 | Aco | TRUE | TRUE | Sch_Vvi |
| Aco_Vvi_Sch | Vvi | 0 | 0.281665 | 0.663764 | 0.336236 | 0.050172 | 0.052949 | -68.87095 | -74.54122 | 20 | 5.670271 | 0.005567 | Aco | FALSE | TRUE | Aco_Sch |
| Aco_Vvi_Sch | Sch | 0 | 3.990603 | 0.612156 | 0.387844 | 0.031215 | 0.070716 | -100.5705 | -105.3429 | 33 | 4.772445 | 0.010595 | Aco | FALSE | TRUE | Aco_Vvi |
| Aco_Cja_Sch | Aco | 0 | 2.642234 | 0.010626 | 0.989374 | 0.03128 | 0.088395 | -4448.047 | -3292.574 | 1157 | -1155.473 | 0.947604 | Aco | TRUE | TRUE | Cja_Sch |
| Aco_Cja_Sch | Cja | 0 | 10.26192 | 0.933269 | 0.066731 | 0.029819 | 0.050219 | -58.52627 | -57.03285 | 15 | -1.493413 | 0.000829 | Aco | FALSE | TRUE | Aco_Sch |
| Aco_Cja_Sch | Sch | 0 | 4.352902 | 0.639887 | 0.360113 | 0.027036 | 0.063393 | -119.5801 | -123.0219 | 36 | 3.441775 | 0.010732 | Aco | FALSE | TRUE | Aco_Cja |
| Nnu_Byu_Tar | Nnu | 0 | 1.330617 | 0.124524 | 0.875476 | 0.014231 | 0.02465 | -4949.145 | -4604.503 | 853 | -344.6427 | 0.618196 | Nnu | TRUE | TRUE | Byu_Tar |
| Nnu_Byu_Tar | Byu | 0 | 1.18482 | 0.275792 | 0.724208 | 0.011554 | 0.017742 | -1134.235 | -1104.436 | 183 | -29.79963 | 0.10971 | Nnu | TRUE | TRUE | Nnu_Tar |
| Nnu_Byu_Tar | Tar | 0 | 2.270165 | 0.472704 | 0.527296 | 0.00902 | 0.016613 | -1058.555 | -1060.419 | 172 | 1.863543 | 0.075079 | Nnu | FALSE | TRUE | Byu_Nnu |
| Nnu_Byu_Din | Nnu | 0 | 1.354193 | 0.090914 | 0.909086 | 0.01348 | 0.023968 | -4657.032 | -4286.495 | 786 | -370.5376 | 0.591508 | Nnu | TRUE | TRUE | Byu_Din |
| Nnu_Byu_Din | Byu | 0 | 1.79046 | 0.253257 | 0.746743 | 0.00921 | 0.017081 | -897.5751 | -873.003 | 143 | -24.57212 | 0.088398 | Nnu | TRUE | TRUE | Din_Nnu |
| Nnu_Byu_Din | Din | 0 | 0.524158 | 0.567686 | 0.432314 | 0.031529 | 0.035808 | -1293.876 | -1294.282 | 279 | 0.406352 | 0.099847 | Nnu | FALSE | TRUE | Byu_Nnu |
| Nnu_Byu_Vvi | Nnu | 0 | 1.352881 | 0.088093 | 0.911907 | 0.013489 | 0.024016 | -4652.319 | -4277.931 | 785 | -374.3877 | 0.592589 | Nnu | TRUE | TRUE | Byu_Vvi |
| Nnu_Byu_Vvi | Byu | 0 | 1.728417 | 0.264632 | 0.735368 | 0.009541 | 0.01729 | -891.7296 | -869.5256 | 143 | -22.20403 | 0.087051 | Nnu | TRUE | TRUE | Nnu_Vvi |
| Nnu_Byu_Vvi | Vvi | 0 | 0.90638 | 0.598966 | 0.401034 | 0.026653 | 0.033046 | -1336.684 | -1343.877 | 280 | 7.193473 | 0.092955 | Nnu | FALSE | TRUE | Byu_Nnu |
| Nnu_Byu_Cja | Nnu | 0 | 1.360401 | 0.088421 | 0.911579 | 0.013415 | 0.023952 | -4662.635 | -4287.556 | 786 | -375.0791 | 0.59313 | Nnu | TRUE | TRUE | Byu_Cja |
| Nnu_Byu_Cja | Byu | 0 | 1.770853 | 0.262147 | 0.737853 | 0.00947 | 0.01735 | -896.6928 | -874.6229 | 144 | -22.06987 | 0.087956 | Nnu | TRUE | TRUE | Cja_Nnu |
| Nnu_Byu_Cja | Cja | 0 | 0.955033 | 0.570746 | 0.429254 | 0.025295 | 0.032171 | -1344.641 | -1349.169 | 278 | 4.527415 | 0.098785 | Nnu | FALSE | TRUE | Byu_Nnu |
| Nnu_Byu_Sch | Nnu | 0 | 1.324 | 0.096299 | 0.903701 | 0.013782 | 0.02421 | -4630.286 | -4270.74 | 786 | -359.5457 | 0.588004 | Nnu | TRUE | TRUE | Byu_Sch |
| Nnu_Byu_Sch | Byu | 0 | 1.565554 | 0.280235 | 0.719765 | 0.010533 | 0.018102 | -875.8831 | -856.388 | 143 | -19.49515 | 0.085204 | Nnu | TRUE | TRUE | Nnu_Sch |
| Nnu_Byu_Sch | Sch | 0 | 0.356998 | 0.599297 | 0.400703 | 0.037055 | 0.040124 | -1228.921 | -1230.768 | 279 | 1.846855 | 0.092547 | Nnu | FALSE | TRUE | Byu_Nnu |
| Nnu_Tar_Din | Nnu | 0 | 1.467245 | 0.080556 | 0.919444 | 0.01229 | 0.022764 | -4898.449 | -4489.974 | 808 | -408.4749 | 0.614993 | Nnu | TRUE | TRUE | Din_Tar |
| Nnu_Tar_Din | Tar | 0 | 1.793157 | 0.346212 | 0.653788 | 0.007701 | 0.013928 | -841.9571 | -833.2475 | 128 | -8.70964 | 0.069276 | Nnu | FALSE | TRUE | Din_Nnu |
| Nnu_Tar_Din | Din | 0 | 0.343316 | 0.552799 | 0.447201 | 0.032814 | 0.035735 | -1269.366 | -1262.793 | 272 | -6.573094 | 0.100694 | Nnu | FALSE | TRUE | Nnu_Tar |
| Nnu_Tar_Vvi | Nnu | 0 | 1.457592 | 0.080377 | 0.919623 | 0.012371 | 0.022841 | -4892.899 | -4484.515 | 808 | -408.3839 | 0.615112 | Nnu | TRUE | TRUE | Tar_Vvi |
| Nnu_Tar_Vvi | Tar | 0 | 1.793157 | 0.346212 | 0.653788 | 0.007701 | 0.013928 | -841.9571 | -833.2475 | 128 | -8.70964 | 0.069276 | Nnu | FALSE | TRUE | Nnu_Vvi |
| Nnu_Tar_Vvi | Vvi | 0 | 0.622506 | 0.587283 | 0.412717 | 0.028501 | 0.032915 | -1305.477 | -1307.521 | 272 | 2.044047 | 0.09293 | Nnu | FALSE | TRUE | Nnu_Tar |
| Nnu_Tar_Cja | Nnu | 0 | 1.461987 | 0.080136 | 0.919864 | 0.012334 | 0.022816 | -4895.409 | -4486.254 | 808 | -409.1546 | 0.615273 | Nnu | TRUE | TRUE | Cja_Tar |
| Nnu_Tar_Cja | Tar | 0 | 1.807752 | 0.340492 | 0.659508 | 0.007638 | 0.013929 | -849.8305 | -839.7794 | 129 | -10.05115 | 0.070428 | Nnu | TRUE | TRUE | Cja_Nnu |
| Nnu_Tar_Cja | Cja | 0 | 0.652219 | 0.566484 | 0.433516 | 0.027202 | 0.03185 | -1320.567 | -1320.52 | 271 | -0.047383 | 0.097254 | Nnu | FALSE | TRUE | Nnu_Tar |
| Nnu_Tar_Sch | Nnu | 0 | 1.373084 | 0.087878 | 0.912122 | 0.012923 | 0.023189 | -4831.442 | -4443.494 | 805 | -387.9484 | 0.60783 | Nnu | TRUE | TRUE | Sch_Tar |
| Nnu_Tar_Sch | Tar | 0 | 1.380231 | 0.330826 | 0.669174 | 0.008897 | 0.014561 | -846.7772 | -834.7825 | 130 | -11.99469 | 0.072014 | Nnu | TRUE | TRUE | Nnu_Sch |
| Nnu_Tar_Sch | Sch | 0 | 0.272788 | 0.572511 | 0.427489 | 0.037401 | 0.039884 | -1210.263 | -1207.48 | 273 | -2.783465 | 0.09661 | Nnu | FALSE | TRUE | Nnu_Tar |
| Nnu_Din_Vvi | Nnu | 0 | 2.73898 | 0.003252 | 0.996748 | 0.029186 | 0.086284 | -4734.298 | -3415.202 | 1180 | -1319.096 | 0.973645 | Nnu | TRUE | TRUE | Din_Vvi |
| Nnu_Din_Vvi | Din | 0 | 0.98427 | 0.090901 | 0.909099 | 0.03014 | 0.047877 | -91.19598 | -86.63055 | 22 | -4.565439 | 0.016556 | Nnu | FALSE | TRUE | Nnu_Vvi |
| Nnu_Din_Vvi | Vvi | 0 | 1.131081 | 0.15273 | 0.84727 | 0.029192 | 0.047678 | -21.41915 | -22.72756 | 6 | 1.308405 | 0.004208 | Nnu | FALSE | TRUE | Din_Nnu |
| Nnu_Din_Cja | Nnu | 0 | 2.858511 | 0.002757 | 0.997243 | 0.028048 | 0.085806 | -4783.37 | -3428.308 | 1180 | -1355.062 | 0.974128 | Nnu | TRUE | TRUE | Cja_Din |
| Nnu_Din_Cja | Din | 0 | 1.018192 | 0.286299 | 0.713701 | 0.028907 | 0.042286 | -112.4754 | -113.5225 | 27 | 1.047126 | 0.015952 | Nnu | FALSE | TRUE | Cja_Nnu |
| Nnu_Din_Cja | Cja | 0 | 0 | 0 | 0 | 0 | 0 | 0 | 0 | 1 | 0 | 0 | Nnu | FALSE | TRUE | Din_Nnu |
| Nnu_Din_Sch | Nnu | 0 | 2.447116 | 0.006765 | 0.993235 | 0.034422 | 0.09288 | -4377.256 | -3219.33 | 1172 | -1157.926 | 0.963635 | Nnu | TRUE | TRUE | Din_Sch |
| Nnu_Din_Sch | Din | 0 | 0.996699 | 0.198848 | 0.801152 | 0.029764 | 0.044079 | -55.48212 | -56.77051 | 14 | 1.288386 | 0.009285 | Nnu | FALSE | TRUE | Nnu_Sch |
| Nnu_Din_Sch | Sch | 0 | 2.594094 | 0.493404 | 0.506596 | 0.038406 | 0.076155 | -61.0312 | -66.20829 | 22 | 5.177094 | 0.009226 | Nnu | FALSE | TRUE | Din_Nnu |
| Nnu_Vvi_Cja | Nnu | 0 | 2.826576 | 0.002476 | 0.997524 | 0.029248 | 0.089606 | -4704.329 | -3328.854 | 1181 | -1375.475 | 0.975228 | Nnu | TRUE | TRUE | Cja_Vvi |
| Nnu_Vvi_Cja | Vvi | 0 | 2.298123 | 0.256904 | 0.743096 | 0.015751 | 0.035935 | -70.93767 | -71.66076 | 16 | 0.723087 | 0.009842 | Nnu | FALSE | TRUE | Cja_Nnu |
| Nnu_Vvi_Cja | Cja | 0 | 3.266703 | 0.387973 | 0.612027 | 0.008524 | 0.021774 | -57.7208 | -59.79657 | 11 | 2.075775 | 0.005573 | Nnu | FALSE | TRUE | Nnu_Vvi |
| Nnu_Vvi_Sch | Nnu | 0 | 2.528099 | 0.007055 | 0.992945 | 0.03166 | 0.087411 | -4537.085 | -3358.696 | 1171 | -1178.389 | 0.962532 | Nnu | TRUE | TRUE | Sch_Vvi |
| Nnu_Vvi_Sch | Vvi | 0 | 2.007464 | 0.295754 | 0.704246 | 0.016448 | 0.032958 | -48.366 | -50.6779 | 11 | 2.311897 | 0.006413 | Nnu | FALSE | TRUE | Nnu_Sch |
| Nnu_Vvi_Sch | Sch | 0 | 3.21481 | 0.538769 | 0.461231 | 0.038747 | 0.084156 | -68.44354 | -73.44633 | 26 | 5.002792 | 0.009927 | Nnu | FALSE | TRUE | Nnu_Vvi |
| Nnu_Cja_Sch | Nnu | 0 | 2.730853 | 0.005263 | 0.994737 | 0.028889 | 0.084614 | -4697.087 | -3434.86 | 1171 | -1262.226 | 0.964269 | Nnu | TRUE | TRUE | Cja_Sch |
| Nnu_Cja_Sch | Cja | 0 | 3.594881 | 0.429283 | 0.570717 | 0.009368 | 0.0234 | -29.00741 | -31.26832 | 6 | 2.260902 | 0.002835 | Nnu | FALSE | TRUE | Nnu_Sch |
| Nnu_Cja_Sch | Sch | 0 | 3.369533 | 0.602563 | 0.397437 | 0.035097 | 0.074238 | -89.95411 | -95.79612 | 31 | 5.842011 | 0.010199 | Nnu | FALSE | TRUE | Cja_Nnu |
| Byu_Tar_Din | Byu | 0 | 0.742114 | 0.183214 | 0.816786 | 0.010462 | 0.014338 | -2194.694 | -2103.373 | 325 | -91.32094 | 0.219748 | Din | TRUE | TRUE | Din_Tar |
| Byu_Tar_Din | Tar | 0 | 0.957691 | 0.360773 | 0.639227 | 0.009117 | 0.012704 | -2392.03 | -2350.252 | 350 | -41.77753 | 0.185206 | Din | TRUE | TRUE | Byu_Din |
| Byu_Tar_Din | Din | 0 | 0.335573 | 0.497727 | 0.502273 | 0.023468 | 0.025829 | -2852.359 | -2825.295 | 533 | -27.06418 | 0.221615 | Din | TRUE | TRUE | Byu_Tar |
| Byu_Tar_Vvi | Byu | 0 | 0.763758 | 0.189518 | 0.810482 | 0.010519 | 0.014504 | -2183.494 | -2095.891 | 325 | -87.60232 | 0.218052 | Vvi | TRUE | TRUE | Tar_Vvi |
| Byu_Tar_Vvi | Tar | 0 | 0.975041 | 0.352416 | 0.647584 | 0.009009 | 0.012669 | -2389.165 | -2345.449 | 349 | -43.71562 | 0.187092 | Vvi | TRUE | TRUE | Byu_Vvi |
| Byu_Tar_Vvi | Vvi | 0 | 0.378297 | 0.463909 | 0.536091 | 0.021774 | 0.024328 | -2929.185 | -2894.531 | 534 | -34.65417 | 0.236981 | Vvi | TRUE | TRUE | Byu_Tar |
| Byu_Tar_Cja | Byu | 0 | 0.72631 | 0.189881 | 0.810119 | 0.010689 | 0.014531 | -2189.809 | -2101.135 | 326 | -88.67464 | 0.218625 | Cja | TRUE | TRUE | Cja_Tar |
| Byu_Tar_Cja | Tar | 0 | 1.010721 | 0.369665 | 0.630335 | 0.00898 | 0.012611 | -2401.071 | -2362.107 | 351 | -38.96418 | 0.183152 | Cja | TRUE | TRUE | Byu_Cja |
| Byu_Tar_Cja | Cja | 0 | 0.389098 | 0.447767 | 0.552233 | 0.021285 | 0.023942 | -2933.968 | -2895.25 | 531 | -38.71858 | 0.242745 | Cja | TRUE | TRUE | Byu_Tar |
| Byu_Tar_Sch | Byu | 0 | 0.649095 | 0.224933 | 0.775067 | 0.011709 | 0.015289 | -2130.793 | -2055.276 | 324 | -75.51674 | 0.207882 | Sch | TRUE | TRUE | Sch_Tar |
| Byu_Tar_Sch | Tar | 0 | 0.908377 | 0.376399 | 0.623601 | 0.009611 | 0.013087 | -2387.937 | -2349.431 | 353 | -38.50613 | 0.182228 | Sch | TRUE | TRUE | Byu_Sch |
| Byu_Tar_Sch | Sch | 0 | 0.294218 | 0.544392 | 0.455608 | 0.026169 | 0.028164 | -2739.267 | -2722.763 | 531 | -16.50398 | 0.200271 | Sch | TRUE | TRUE | Byu_Tar |
| Byu_Din_Vvi | Byu | 0 | 2.661052 | 0.002391 | 0.997609 | 0.024972 | 0.072597 | -5126.037 | -3813.079 | 1177 | -1312.958 | 0.972009 | Byu | TRUE | TRUE | Din_Vvi |
| Byu_Din_Vvi | Din | 0 | 1.3373 | 0.19104 | 0.80896 | 0.026904 | 0.046317 | -93.48608 | -92.18825 | 23 | -1.297835 | 0.015402 | Byu | FALSE | TRUE | Byu_Vvi |
| Byu_Din_Vvi | Vvi | 0 | 1.277253 | 0.443271 | 0.556729 | 0.025851 | 0.039336 | -30.23975 | -33.69037 | 8 | 3.450621 | 0.003687 | Byu | FALSE | TRUE | Byu_Din |
| Byu_Din_Cja | Byu | 0 | 2.87813 | 0.001119 | 0.998881 | 0.023266 | 0.07231 | -5221.772 | -3812.65 | 1174 | -1409.121 | 0.970766 | Byu | TRUE | TRUE | Cja_Din |
| Byu_Din_Cja | Din | 0 | 0.993027 | 0.579308 | 0.420692 | 0.029712 | 0.038297 | -121.771 | -127.851 | 29 | 6.079977 | 0.010099 | Byu | FALSE | TRUE | Byu_Cja |
| Byu_Din_Cja | Cja | 0 | 5.36272 | 0.752859 | 0.247141 | 0.007284 | 0.015317 | -28.30396 | -30.17825 | 5 | 1.874288 | 0.001023 | Byu | FALSE | TRUE | Byu_Din |
| Byu_Din_Sch | Byu | 0 | 2.271116 | 0.006653 | 0.993347 | 0.030924 | 0.079113 | -4697.119 | -3586.149 | 1169 | -1110.97 | 0.961277 | Byu | TRUE | TRUE | Din_Sch |
| Byu_Din_Sch | Din | 0 | 0.984784 | 0.469591 | 0.530409 | 0.030124 | 0.040335 | -67.96487 | -72.32488 | 17 | 4.360006 | 0.007464 | Byu | FALSE | TRUE | Byu_Sch |
| Byu_Din_Sch | Sch | 0 | 2.85855 | 0.446192 | 0.553808 | 0.034853 | 0.077288 | -61.7848 | -65.55831 | 22 | 3.773509 | 0.010086 | Byu | FALSE | TRUE | Byu_Din |
| Byu_Vvi_Cja | Byu | 0 | 2.845208 | 0.001518 | 0.998482 | 0.024663 | 0.076035 | -5101.938 | -3704.141 | 1177 | -1397.797 | 0.972859 | Byu | TRUE | TRUE | Cja_Vvi |
| Byu_Vvi_Cja | Vvi | 0 | 2.608302 | 0.470988 | 0.529012 | 0.015022 | 0.031771 | -80.33074 | -85.28076 | 18 | 4.950023 | 0.007883 | Byu | FALSE | TRUE | Byu_Cja |
| Byu_Vvi_Cja | Cja | 0 | 3.313732 | 0.42165 | 0.57835 | 0.008853 | 0.022188 | -67.74425 | -70.44812 | 13 | 2.703874 | 0.006224 | Byu | FALSE | TRUE | Byu_Vvi |
| Byu_Vvi_Sch | Byu | 0 | 2.479804 | 0.005954 | 0.994046 | 0.027122 | 0.073728 | -4911.226 | -3744.56 | 1167 | -1166.666 | 0.960308 | Byu | TRUE | TRUE | Sch_Vvi |
| Byu_Vvi_Sch | Vvi | 0 | 1.4998 | 0.3295 | 0.6705 | 0.020473 | 0.034591 | -60.71078 | -63.55773 | 14 | 2.846946 | 0.007771 | Byu | FALSE | TRUE | Byu_Sch |
| Byu_Vvi_Sch | Sch | 0 | 3.384804 | 0.529866 | 0.470134 | 0.036801 | 0.083315 | -72.07755 | -76.90094 | 27 | 4.823389 | 0.010508 | Byu | FALSE | TRUE | Byu_Vvi |
| Byu_Cja_Sch | Byu | 0 | 2.783619 | 0.00359 | 0.99641 | 0.023868 | 0.070978 | -5123.05 | -3829.982 | 1166 | -1293.068 | 0.961767 | Byu | TRUE | TRUE | Cja_Sch |
| Byu_Cja_Sch | Cja | 0 | 1.951963 | 0.545567 | 0.454433 | 0.014826 | 0.025756 | -46.21143 | -50.87931 | 10 | 4.667877 | 0.003762 | Byu | FALSE | TRUE | Byu_Sch |
| Byu_Cja_Sch | Sch | 0 | 3.800907 | 0.5864 | 0.4136 | 0.031114 | 0.07207 | -96.48301 | -100.8621 | 32 | 4.379054 | 0.010956 | Byu | FALSE | TRUE | Byu_Cja |
| Tar_Din_Vvi | Tar | 0 | 2.744237 | 0.001415 | 0.998585 | 0.024081 | 0.072523 | -5192.818 | -3808.977 | 1175 | -1383.841 | 0.971305 | Tar | TRUE | TRUE | Din_Vvi |
| Tar_Din_Vvi | Din | 0 | 1.357125 | 0.231948 | 0.768052 | 0.025823 | 0.043303 | -108.2603 | -107.9978 | 26 | -0.262566 | 0.016531 | Tar | FALSE | TRUE | Tar_Vvi |
| Tar_Din_Vvi | Vvi | 0 | 1.205736 | 0.353726 | 0.646274 | 0.027384 | 0.042346 | -25.45489 | -28.32054 | 7 | 2.865653 | 0.003745 | Tar | FALSE | TRUE | Din_Tar |
| Tar_Din_Cja | Tar | 0 | 2.92555 | 0.000711 | 0.999289 | 0.022708 | 0.072176 | -5270.91 | -3813.736 | 1173 | -1457.175 | 0.970336 | Tar | TRUE | TRUE | Cja_Din |
| Tar_Din_Cja | Din | 0 | 1.072233 | 0.424167 | 0.575833 | 0.02745 | 0.038457 | -132.7128 | -136.5752 | 31 | 3.862395 | 0.014777 | Tar | FALSE | TRUE | Cja_Tar |
| Tar_Din_Cja | Cja | 0 | 0.80565 | 0.528283 | 0.471717 | 0.011849 | 0.015277 | -21.56525 | -24.0651 | 4 | 2.499856 | 0.001562 | Tar | FALSE | TRUE | Din_Tar |
| Tar_Din_Sch | Tar | 0 | 2.337838 | 0.006859 | 0.993141 | 0.030053 | 0.079018 | -4727.956 | -3585.895 | 1168 | -1142.061 | 0.960256 | Tar | TRUE | TRUE | Din_Sch |
| Tar_Din_Sch | Din | 0 | 0.666011 | 0.69507 | 0.30493 | 0.045338 | 0.051802 | -54.41436 | -59.95776 | 16 | 5.543399 | 0.004039 | Tar | FALSE | TRUE | Sch_Tar |
| Tar_Din_Sch | Sch | 0 | 3.070134 | 0.510442 | 0.489558 | 0.032451 | 0.071804 | -70.4643 | -75.24516 | 24 | 4.780859 | 0.009726 | Tar | FALSE | TRUE | Din_Tar |
| Tar_Vvi_Cja | Tar | 0 | 2.912815 | 0.000674 | 0.999326 | 0.023881 | 0.07592 | -5168.153 | -3701.41 | 1175 | -1466.744 | 0.972027 | Tar | TRUE | TRUE | Cja_Vvi |
| Tar_Vvi_Cja | Vvi | 0 | 2.49538 | 0.482437 | 0.517563 | 0.015702 | 0.031297 | -85.59306 | -90.69597 | 19 | 5.102917 | 0.00814 | Tar | FALSE | TRUE | Cja_Tar |
| Tar_Vvi_Cja | Cja | 0 | 3.469763 | 0.479336 | 0.520664 | 0.008455 | 0.020932 | -74.26978 | -77.62283 | 14 | 3.353043 | 0.006034 | Tar | FALSE | TRUE | Tar_Vvi |
| Tar_Vvi_Sch | Tar | 0 | 2.53189 | 0.005212 | 0.994788 | 0.026567 | 0.073565 | -4940.911 | -3746.501 | 1166 | -1194.411 | 0.960201 | Tar | TRUE | TRUE | Sch_Vvi |
| Tar_Vvi_Sch | Vvi | 0 | 0.763815 | 0.729066 | 0.270934 | 0.0402 | 0.046205 | -42.20596 | -47.30687 | 12 | 5.100914 | 0.002691 | Tar | FALSE | TRUE | Sch_Tar |
| Tar_Vvi_Sch | Sch | 0 | 3.718024 | 0.586599 | 0.413401 | 0.033503 | 0.075946 | -86.05437 | -91.26279 | 30 | 5.208414 | 0.010267 | Tar | FALSE | TRUE | Tar_Vvi |
| Tar_Cja_Sch | Tar | 0 | 2.841941 | 0.004011 | 0.995989 | 0.023332 | 0.070741 | -5151.928 | -3837.79 | 1166 | -1314.138 | 0.96136 | Tar | TRUE | TRUE | Cja_Sch |
| Tar_Cja_Sch | Cja | 0 | 13.99846 | 0.874997 | 0.125003 | 0.014307 | 0.039331 | -38.31141 | -33.69238 | 8 | -4.619028 | 0.000828 | Tar | FALSE | TRUE | Sch_Tar |
| Tar_Cja_Sch | Sch | 0 | 3.922674 | 0.615804 | 0.384196 | 0.030148 | 0.068829 | -105.7975 | -110.4509 | 34 | 4.653484 | 0.010813 | Tar | FALSE | TRUE | Cja_Tar |
| Din_Vvi_Cja | Din | 0 | 1.084719 | 0.282417 | 0.717583 | 0.009276 | 0.013806 | -4223.494 | -4103.446 | 626 | -120.0478 | 0.37186 | Din | TRUE | TRUE | Cja_Vvi |
| Din_Vvi_Cja | Vvi | 0 | 0.849684 | 0.461448 | 0.538552 | 0.009302 | 0.012033 | -1997.394 | -1984.811 | 291 | -12.58309 | 0.129734 | Din | TRUE | TRUE | Cja_Din |
| Din_Vvi_Cja | Cja | 0 | 1.323444 | 0.473672 | 0.526328 | 0.008962 | 0.013122 | -1937.579 | -1934.404 | 291 | -3.174705 | 0.126789 | Din | FALSE | TRUE | Din_Vvi |
| Din_Vvi_Sch | Din | 0 | 1.658579 | 0.464475 | 0.535525 | 0.011926 | 0.019222 | -1899.321 | -1895.129 | 322 | -4.191771 | 0.142748 | Vvi | FALSE | TRUE | Sch_Vvi |
| Din_Vvi_Sch | Vvi | 0 | 0.847992 | 0.366011 | 0.633989 | 0.015795 | 0.021142 | -3423.292 | -3352.872 | 588 | -70.42004 | 0.308597 | Vvi | TRUE | TRUE | Din_Sch |
| Din_Vvi_Sch | Sch | 0 | 0.688965 | 0.500639 | 0.499361 | 0.01872 | 0.022752 | -1661.594 | -1653.03 | 298 | -8.563783 | 0.123187 | Vvi | FALSE | TRUE | Din_Vvi |
| Din_Cja_Sch | Din | 0 | 0.577638 | 0.465189 | 0.534811 | 0.012803 | 0.015234 | -1605.084 | -1592.971 | 251 | -12.1124 | 0.111124 | Cja | TRUE | TRUE | Cja_Sch |
| Din_Cja_Sch | Cja | 0 | 0.981323 | 0.414227 | 0.585773 | 0.015193 | 0.02079 | -3671.195 | -3619.612 | 631 | -51.58234 | 0.305979 | Cja | TRUE | TRUE | Din_Sch |
| Din_Cja_Sch | Sch | 0 | 0.66364 | 0.538978 | 0.461022 | 0.018372 | 0.021935 | -1836.688 | -1832.651 | 326 | -4.036943 | 0.124415 | Cja | FALSE | TRUE | Cja_Din |
| Vvi_Cja_Sch | Vvi | 0 | 0.762964 | 0.43867 | 0.56133 | 0.011247 | 0.014307 | -1624.623 | -1611.492 | 249 | -13.13062 | 0.115705 | Sch | TRUE | TRUE | Cja_Sch |
| Vvi_Cja_Sch | Cja | 0 | 0.932032 | 0.478307 | 0.521693 | 0.013898 | 0.018175 | -2195.393 | -2183.727 | 364 | -11.66637 | 0.157199 | Sch | TRUE | TRUE | Sch_Vvi |
| Vvi_Cja_Sch | Sch | 0 | 0.740263 | 0.492136 | 0.507864 | 0.016291 | 0.020219 | -3472.846 | -3445.956 | 595 | -26.88931 | 0.250148 | Sch | TRUE | TRUE | Cja_Vvi |
